# Supplementary material for: Physical and behavioral adaptations to prevent overheating of the living wings of butterflies
Source: Nat Commun. 2020 Jan 28;11:551. doi: 10.1038/s41467-020-14408-8 (PMC6987309; doi:10.1038/s41467-020-14408-8)
Supplement: Supplementary file 1 — Supplementary Information [file 41467_2020_14408_MOESM1_ESM.pdf]

Supplementary Information

Physical and Behavioral Adaptations to Prevent Overheating of the Living Wings of Butterflies

Tsai *et al.*

Table of Contents

Supplementary Note **1**: Staining of neurons in wing veins with methylene blue 1
Supplementary Note **2**: Imaging living cells in butterfly wing veins using fluorescence microscopy 8
Supplementary Note **3**: Observation of internal structures & hemolymph flow in the wings of *Vanessa cardui* 8
Supplementary Note **4**: Observation of internal structures & hemolymph flow in the wings of lycaenid butterflies..10
Supplementary Note **5**: Persistent hemolymph flow & thermo-sensing of the wing over butterflies' lifetime …...... 12
Supplementary Note **6**: Impact of overheating on the function of butterfly wings 14
Supplementary Note **7**: Effects of hemolymph flow on wing temperature 16
Supplementary Note **8**: Morphological & spectroscopic studies of wing scales of lycaenid butterflies 17
Supplementary Note **9**: Stacking photography of wing scales of lycaenid butterflies 19
Supplementary Note **10**: Infrared hyperspectral imaging of butterfly wings 27
Supplementary Note **11**: Deriving complex refractive indices of chitin 34
Supplementary Note **12**: Experimental setup to simulate the natural radiative environment of butterflies 35
Supplementary Note **13**: Determining temperature distributions over butterfly wings 39
Supplementary Note **14**: Thermodynamic studies of butterfly wings 42
Supplementary Note **15**: Theoretical model for calculating steady-state butterfly wing temperatures 45
Supplementary Note **16**: Laser-induced local heating experiments & controls 50
Supplementary Note **17**: Displacement response induced by local heating on different wing regions 52
Supplementary Note **18**: Heat avoidance & basking behaviors of *Satyrium caryaevorus* in the field 53
Supplementary Note **19**: Butterfly specimens used in this study 54
Supplementary References 56

Supplementary Note 1: Staining of neurons in wing veins with methylene blue

Adult painted lady butterflies, *Vanessa cardui*, and hickory hairstreak butterflies, *Satyrium caryaevorus*, were anesthetized with CO_2. A saturated aqueous solution of methylene blue[1] was injected into the thorax: 50-100 µl used for individual *V. cardui* and 10 µl used for individual *S. caryaevorus*. The stained wings were removed 6 hours after injection, when the butterflies had recovered from anesthesia and the injected stain had been circulated by the hemolymph into the wing veins. The scales were removed from both sides of the stained wings and stacking photography was used to take pictures of stained wing tissues (**Supplementary Figs. 1** and **2**).

a

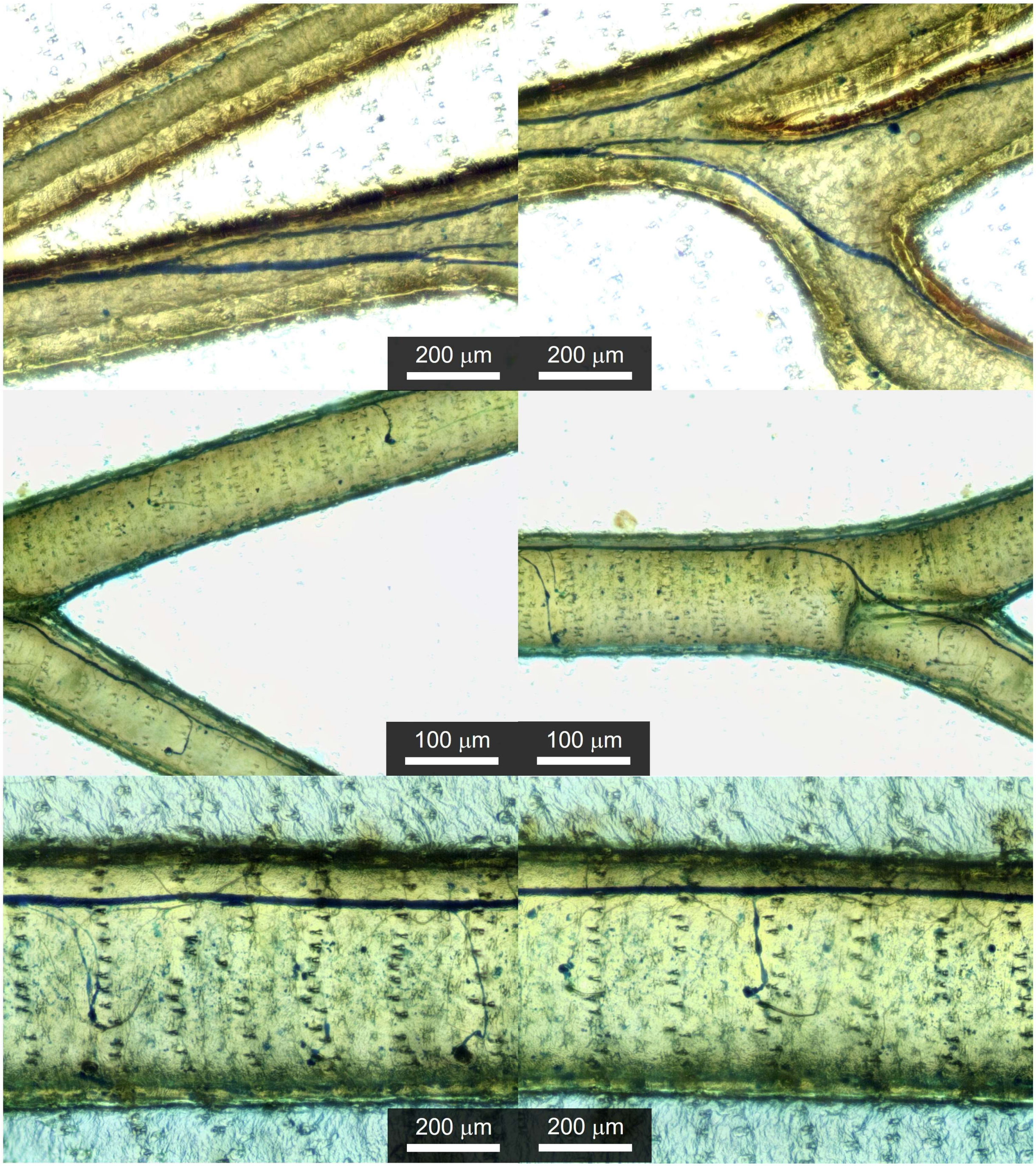

b

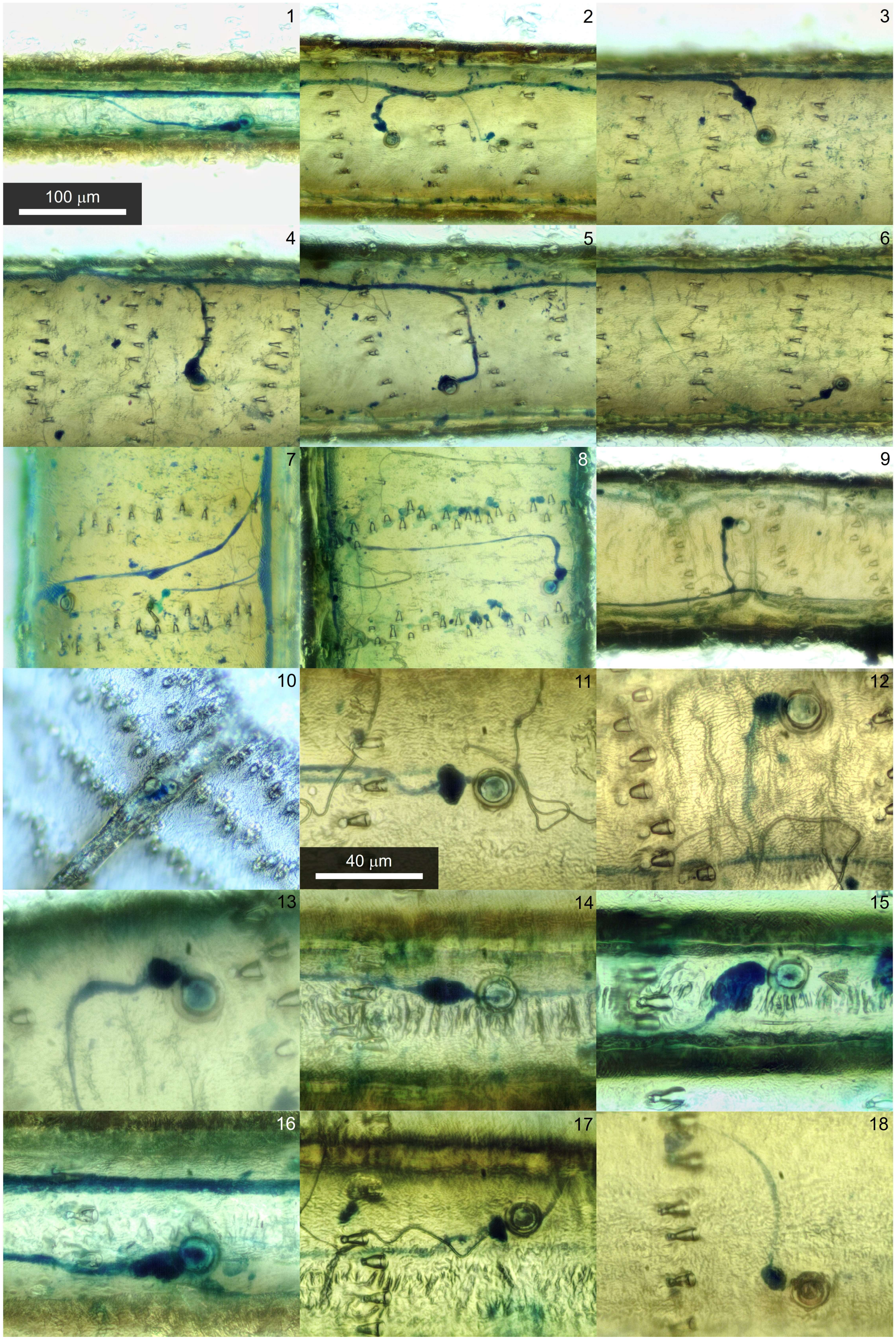

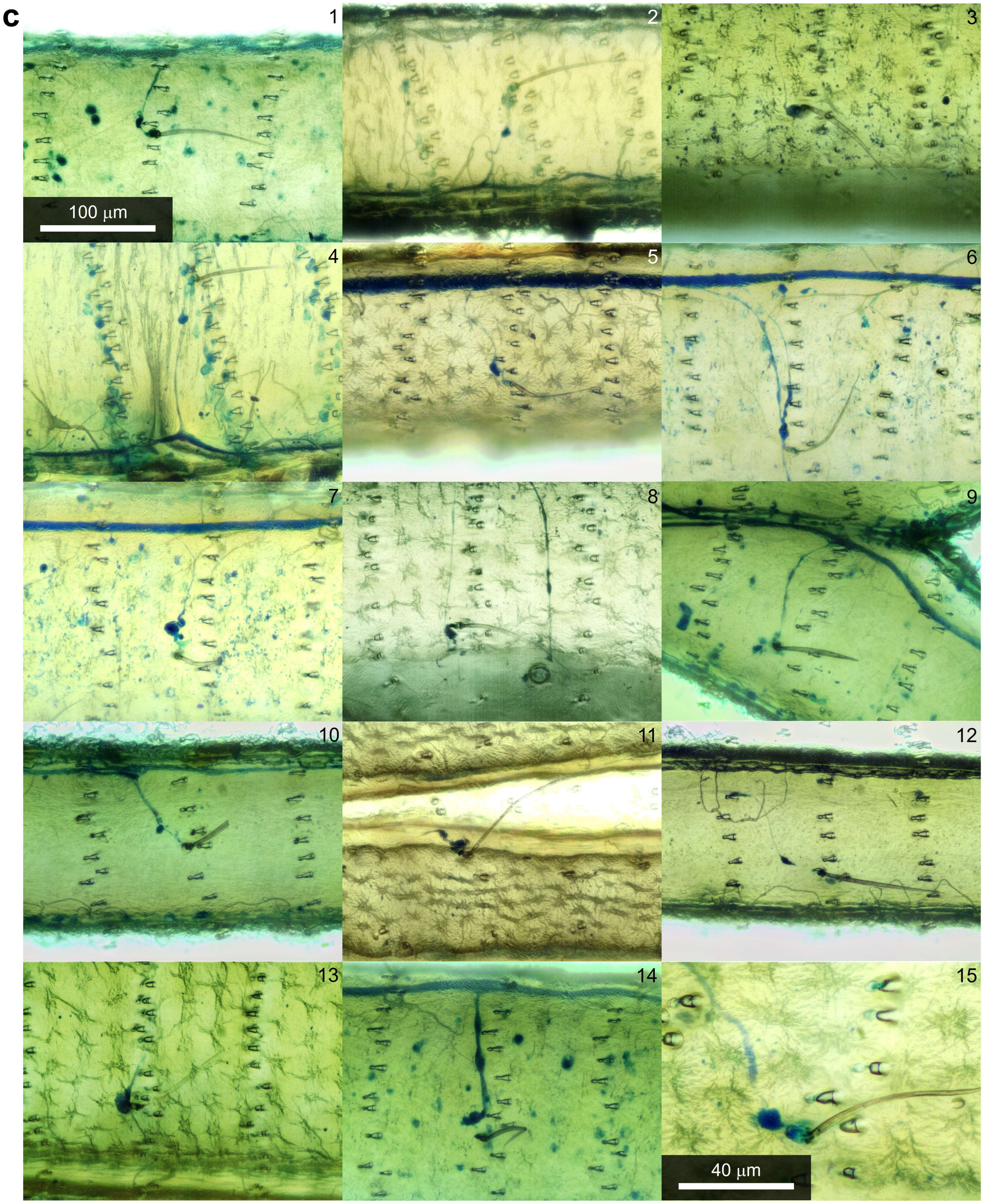
c
1
2
3
100 μm
4
5
6
7
8
9
10
11
12
13
14
15
40 μm

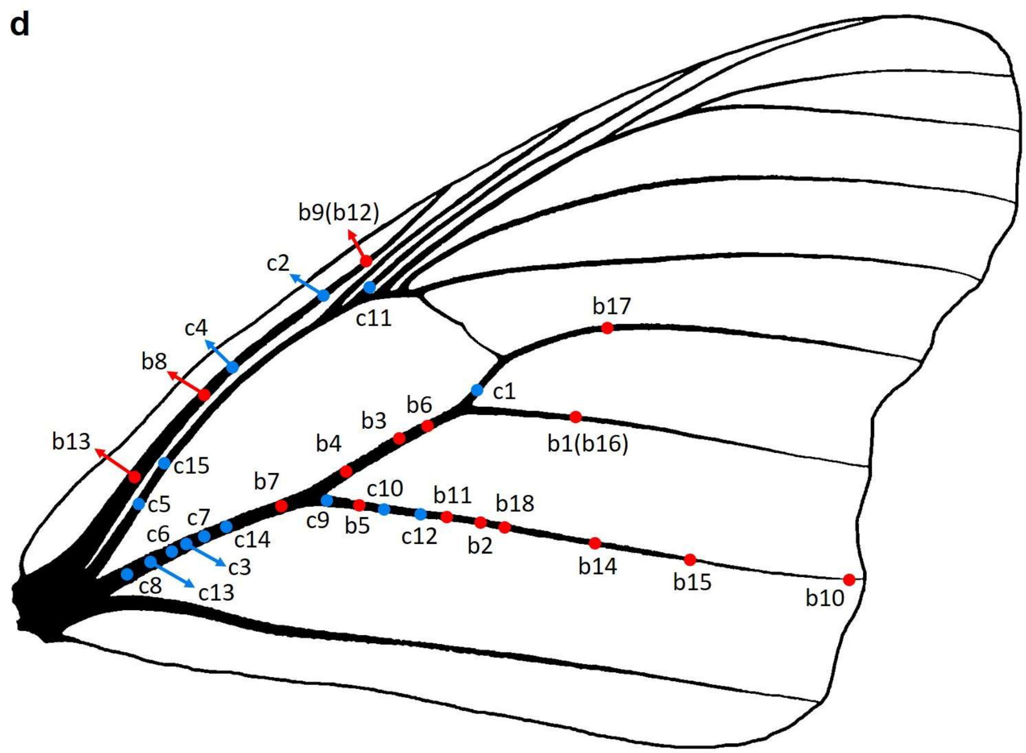

Supplementary Figure 1 | Sensory neurons in the wing veins of *Vanessa cardui* stained with methylene blue. (a) Axons distributed along the wing veins. (b) Campaniform sensilla. (c) Bristle sensilla. These photos were taken with a microscope equipped with a CCD camera. Stacking photography was used to better resolve fine structures. The first 10 photos in (b) and the first 14 photos in (c) were taken with a magnification of ×20, and the last 8 photos in (b) and the last photo in (c) were taken with a higher magnification of ×50. (d) Distribution of stained sensilla. Red and blue dots represent campaniform and bristle sensilla, respectively, and the numbers correspond to those labelled in (b) and (c).

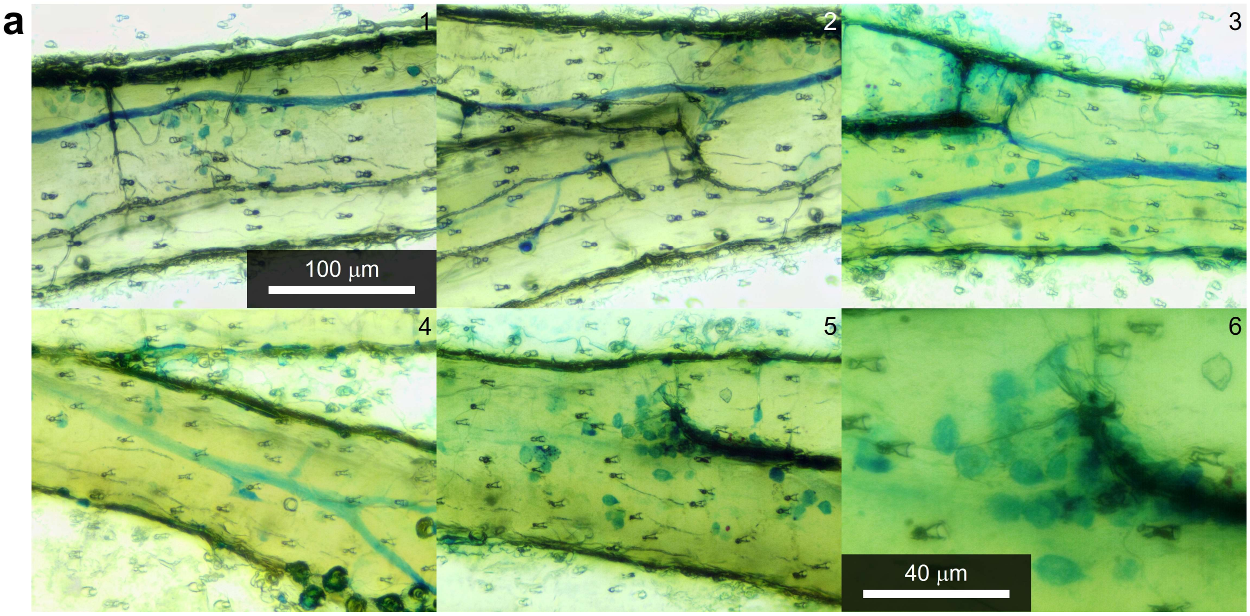

b

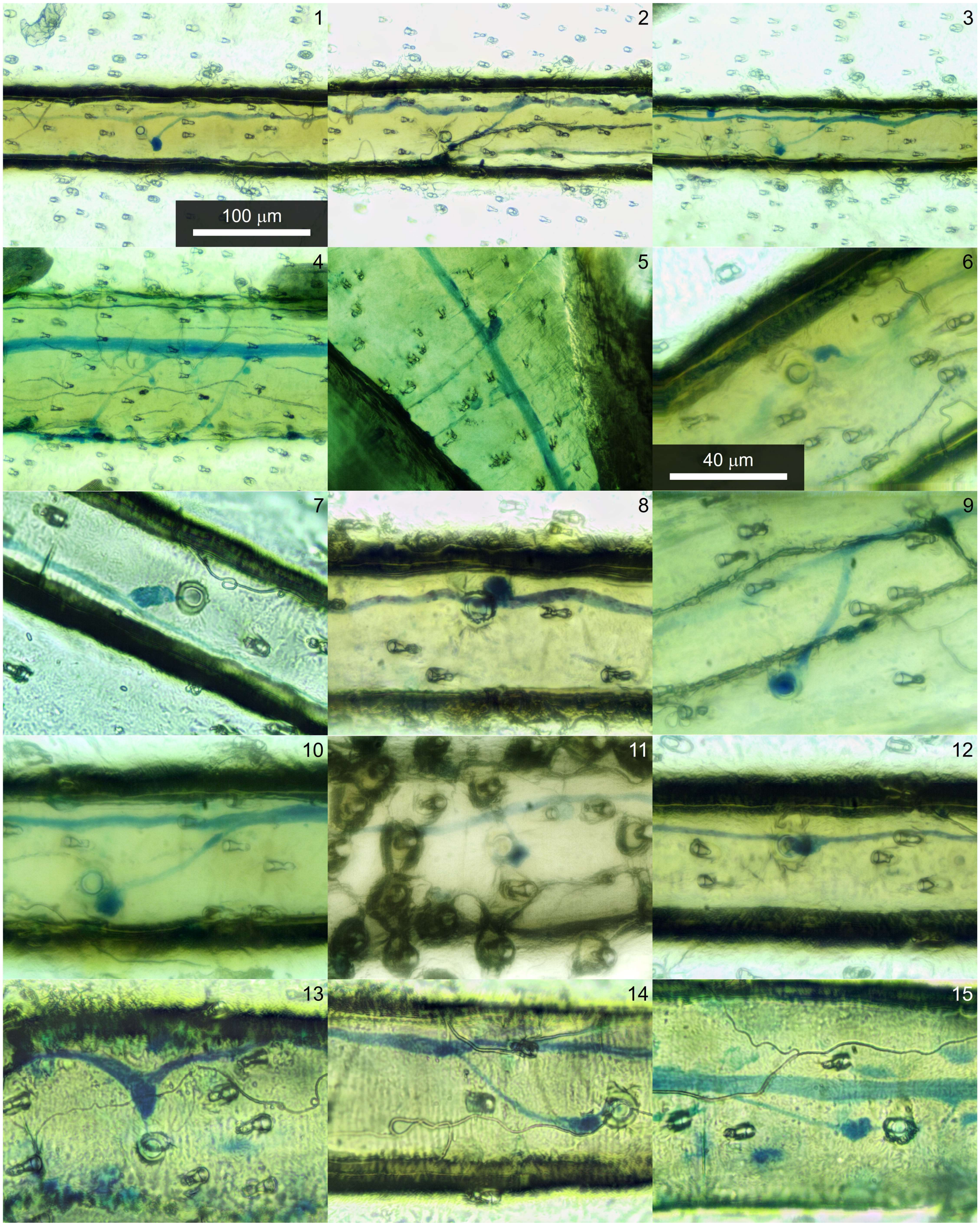

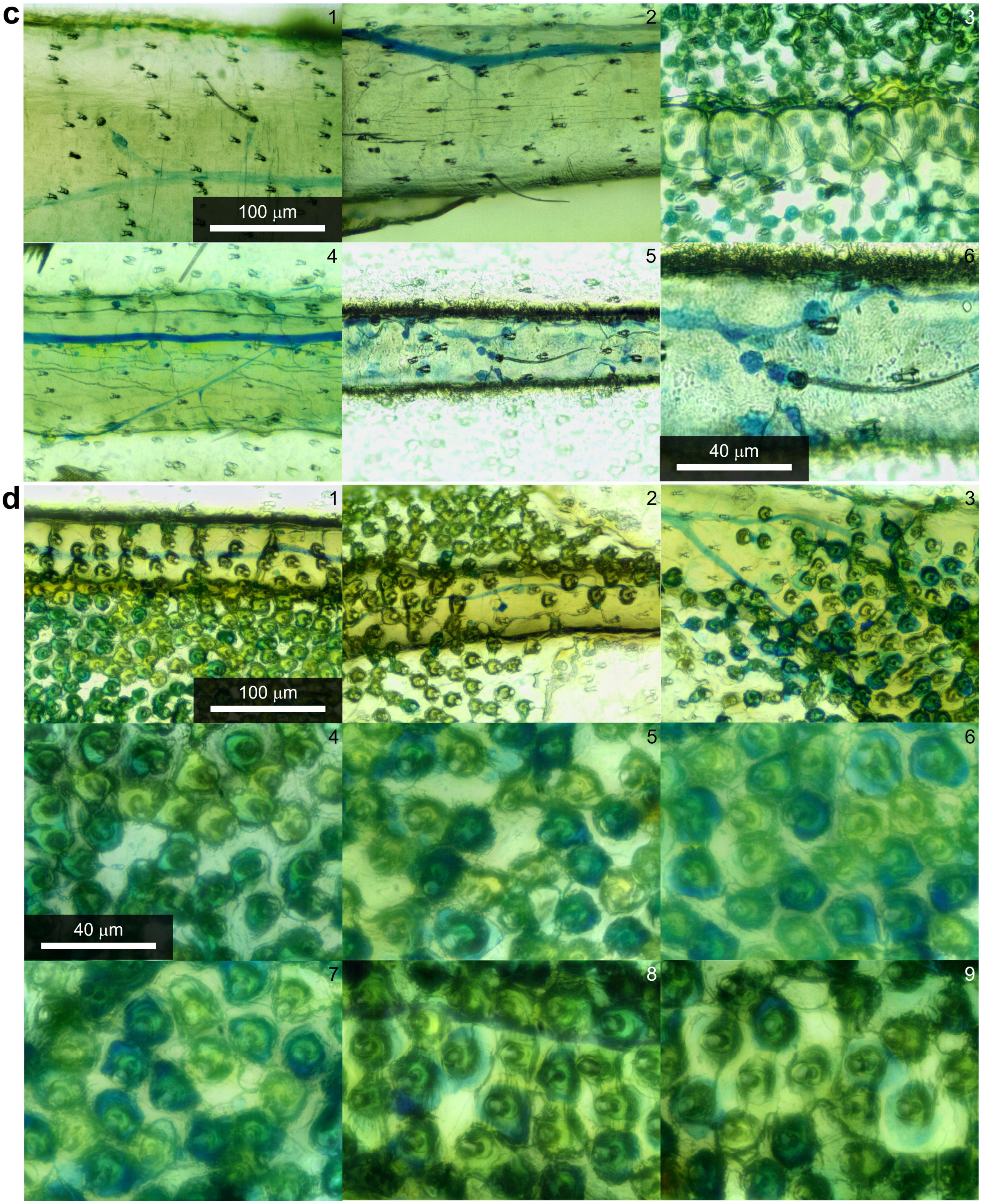

Supplementary Figure 2 | Sensory neurons in the wing veins and living cells in the scent pads of *Satyrium caryaevorus* stained with methylene blue. (a) Axons and cells distributed along the wing veins. (b) Campaniform sensilla. (c) Bristle sensilla. (d) Cells and axons in the scent pad. The first 5 photos in (a), (b), and (c), and the first 3 photos in (d) were taken with a magnification

of ×20; the last photo in (a), the last 10 photos in (b), the last photo in (c), and the last 6 photos in (d) were taken with a higher magnification of ×50.

Supplementary Note 2: Imaging living cells in butterfly wing veins using fluorescence microscopy

A freshly eclosed adult of *V. cardui* was euthanized, and small segments of wing veins excised from the individual after carefully removing the scales. The segments were stained with 0.025% calcein acetoxymethyl ester (Calcein AM, LIVE/DEAD staining kit, Life Technologies, Invitrogen) in phosphate buffered saline (PBS) solution for 30 minutes. Calcein AM is a cell membrane permeable dye commonly used to stain living cells that is converted to calcein after hydrolysis by intracellular esterases. Stained, living cells display green fluorescence (excitation/emission wavelengths = 494/517 nm). **Supplementary Fig. 3** shows fluorescence microscope images of stained living cells and corresponding optical images. These images show that the "dark streaks" observed in wing veins are active, living cells rather than non-living structural features.

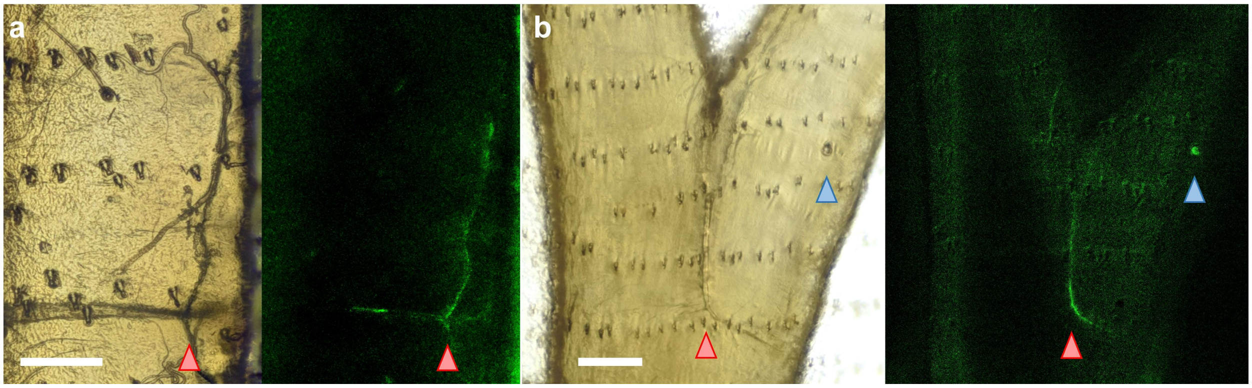

Supplementary Figure 3 | Fluorescence microscope images of stained living cells and corresponding optical images. The cells were stained using a fluorogenic dye, Calcein-AM, that selectively flouresces in living cells, and imaged using a confocal microscope. Images were taken of the subcostal (a) and median (b) veins of the right forewing of *Vanessa cardui* butterflies. Cells marked by red arrows are possibly neural innervations and the one marked by the blue arrow is possibly a campaniform sensillum. Scalar bars in (a): 40 μm, and in (b): 50 μm.

Supplementary Note 3: Observation of internal structures and hemolymph flow in the wings of *Vanessa cardui*

The painted lady butterfly, *Vanessa cardui*, was chosen for studying hemolymph flow because of its intermediate wingspan and because its wing membranes are not suffused with dark pigments and thus enable us to examine the translucent wing veins and hemolymph flow using a transmission-mode optical microscope. Adults of *V. cardui* are notable for making long migratory flights and are unusually robust for experimental manipulations. All butterflies used were purchased from Carolina Biological Supply Company, in the adult or pupal stage.

We carefully removed the scales from butterfly wings with a paint brush and mounted the butterfly with its wings open on a stage composed of glass slides, using a low-melting-point wax (**Supplementary Fig. 4a**). The mounted butterfly was then observed under an optical microscope and videos were recorded. We made several observations regarding the structure of the wing veins and the hemolymph flow within:

(1) **Wing veins contain tracheae and hemolymph channels:** The wing veins provide a mechanical framework for the wing as well as a conduit for air and hemolymph. Each of the larger veins contains a tracheal tube flanked by hemolymph channels. Dissecting wing veins and studying their cross-sections under a scanning electron microscope (SEM) revealed that (a) the tracheal tube is located at the center of the wing vein and occupies most of the volume of the vein, and (b) typically two small hemolymph channels flank the two sides of the trachea, along the points where the wing vein is connected to fused membranes (**Supplementary Fig. 4b**). The tracheal tube is able to contract as the relative volume between the tracheal and hemolymph compartments changes (**Supplementary Movie 3**). Overall, hemolymph occupies only a small portion (~10-25%) of the total volume of the wing veins; as a result, its ability to transport heat and thus aid in thermoregulation of the thorax or wings is limited (see more detailed analyses later).

(2) **Wing veins support a network of sensory neurons:** Optical microscopy reveals at least two types of mechanical sensilla, hair-shaped bristle sensilla and disc-shaped campaniform sensilla (**Fig. 1b–e** of the main text, **Supplementary Fig. 1b,c**), distributed along the wing veins, including those in the interior of the wing and along its margins. The morphology of these sensilla is consistent with those known to function in the wings of other Lepidoptera reported in the literature. The tracheal tube running through the wing vein is accompanied by a network of what appear to be closely connected neurons and axons (**Fig. 1c** of the main text, **Supplementary Fig. 1a**).

(3) **Hemolymph flow in wing veins exhibits a tidal pattern:** Semitransparent cells (hemocytes) can be observed flowing inside the hemolymph channels (**Supplementary Movie 3**). The movement of these cells was observed to monitor the direction and speed of flow. The flow of hemolymph changes directions rhythmically, with contraction of the tracheal tube correlated with the movement of hemolymph from the base of the wing to the distal end, and expansion of the tracheal tube correlated with return of hemolymph from the distal end to the base of the wing (**Fig. 1f** of the main text, **Supplementary Movie 3**). The frequency of this tidal flow of hemolymph and air in and out of the wing veins is on the order of once every few minutes at room temperature if the butterfly is not agitated (**Fig. 1g** of the main text).

We tested the cross-correlation between the rate of change in hemolymph channel width and hemolymph flow speed in the wing veins of 9 individuals, including both sexes. We found that in all individuals, these two metrics are significantly cross-correlated with zero time lag. The average R^2 of these individual significant cross-correlations across all individuals is 0.73, and a t-test of individual R^2 values concludes that these are significantly different from 0 ($t = 19.96$, $df = 8$, p-value = 4.139×10^{-8}, **Supplementary Fig. 4c**). As the change in hemolymph channel width is easier to monitor than tracking individual hemocytes, we used hemolymph channel width change rate as a proxy for hemolymph flow speed.

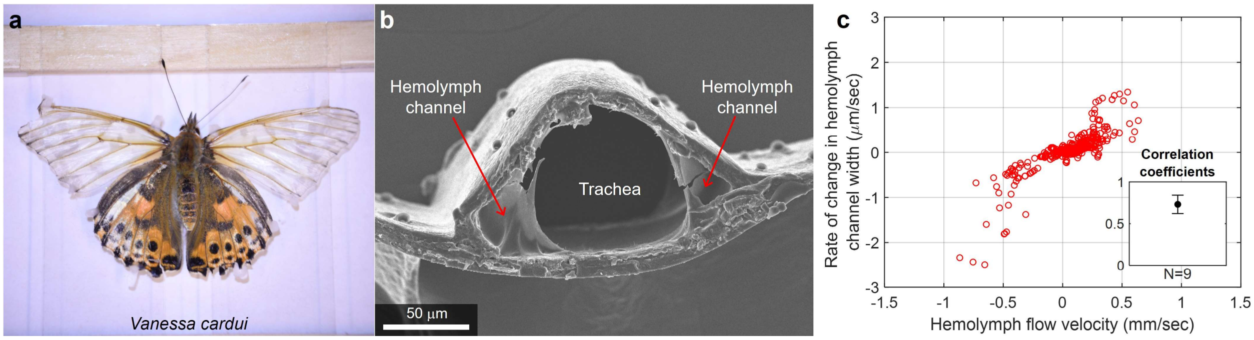

Supplementary Figure 4 | Internal structures and hemolymph flow in *Vanessa cardui* wings. (a) Photo of a living adult *V. cardui* mounted on glass slides with scales removed from both forewings to facilitate examination of hemolymph flow in wing veins. (b) SEM image of the cross-section of a wing vein of *V. cardui*, showing a trachea flanked by two channels carrying hemolymph. (c) Correlation between the hemolymph flow velocity and the rate of change in the hemolymph channel width measured at a radial vein of the forewing of an individual *V. cardui*. Inset shows the correlation coefficients averaged over 9 individuals.

Supplementary Note 4: Observation of internal structures and hemolymph flow in the wings of lycaenid butterflies

We carried out similar studies on field-caught lycaenid butterflies, including the white-M hairstreak, *Parrhasius m-album*, and the hickory hairstreak, *Satyrium caryaevorus.* We found that the wing veins of these butterflies also contains a network of sensory neurons. However, unlike *V. cardui*, the males of these butterflies possess scent pads on their forewings. We made a number of observations regarding the structure of the scent pad and the hemolymph flow in this androconial organ:

(1) **Wing veins contain tracheae and hemolymph channels:** The wing veins contain tracheal tubes and hemolymph channels (**Supplementary Fig. 5a,b**), however, the change of width of the tracheae is much less striking compared to *V. cardui*.

(2) **Wing veins support a network of sensory neurons:** The wing veins contain a network of campaniform sensilla, bristle sensilla, and axons (**Supplementary Figs. 2a,b,c** and **5a**).

(3) **Scent pads contain living cells:** The scent pads are populated with numerous round cells (**Supplementary Fig. 2d**) that are presumably involved in producing pheromones. A dense distribution of dark-colored streaks (blue arrowheads in **Supplementary Fig. 5b**) was also observed in the scent pad, which we speculate to be either nerve fibers or branches of tracheae extending into the scent pad.

(4) **Hemolymph flow in the forewings is unidirectional, but in the hindwings is bidirectional:** In the males of both lycaenid species, the flow of hemolymph in the veins surrounding the forewing discal cell forms a loop (left panel of **Supplementary Fig. 5c**): hemocytes are pumped into the wing from the base of the radius; they channel through the narrow discocellular veins, and return to the thorax via the cubitus. The hindwings show a qualitatively different pattern of hemolymph flow. The flows in the two hemolymph channels of a vein can be in opposite directions simultaneously (right panel of **Supplementary Fig. 5c**): hemocytes flow from the thorax into the wing in one channel and return from the wing back to the thorax in the other. The two hemolymph channels in a vein are connected by inter-channel passages that route hemolymph from the 'outbound' channel to the 'inbound' channel. These inter-channel passages are located in the middle of the vein or at the Y-junction between three veins.

(5) **Male butterflies possess a unique hemolymph flow pattern in scent pads:** For the males of both species, a scent pad is located at the end of the forewing discal cell. The scent pad is entirely filled with hemolymph. Judging from the observed movement of numerous hemocytes, the hemolymph flow in the scent pad is unidirectional (**Fig. 2b,d,e** of the main text, **Supplementary Movies 4–7**): It flows into the scent pad from the radial veins, and is drained into several veins at the bottom distal corner of the scent pad by a "wing heart" that beats endogenously at a rate of a few dozen times per minute (**Supplementary Movies 4** and **5**). The hemolymph flow through the scent pad is rhythmic: it switches between a high flow speed state and a low flow speed state with a frequency of approximately once per minute (**Fig. 2e** of the main text).

In the wings of lycaenid butterflies with scent pads, hemolymph flows in two environments with apparently different hydrodynamic resistances: the hydrodynamic resistance of the wing veins is relatively low so the flow speed is high, and that of the maze of channels in the scent pad is relatively high so the flow speed is low (**Supplementary Movies 4–7** show that the hemocytes appear to trickle through the scent pad). The function of the wing heart may be to add a local pressure gradient to ensure that sufficient hemolymph flows through the scent pad to bathe the cells within it (presumably pheromone-producing cells, neurons, etc.) with nutrients and ions; without the wing heart, the hemolymph might largely bypass the scent pad via the adjacent wing veins.

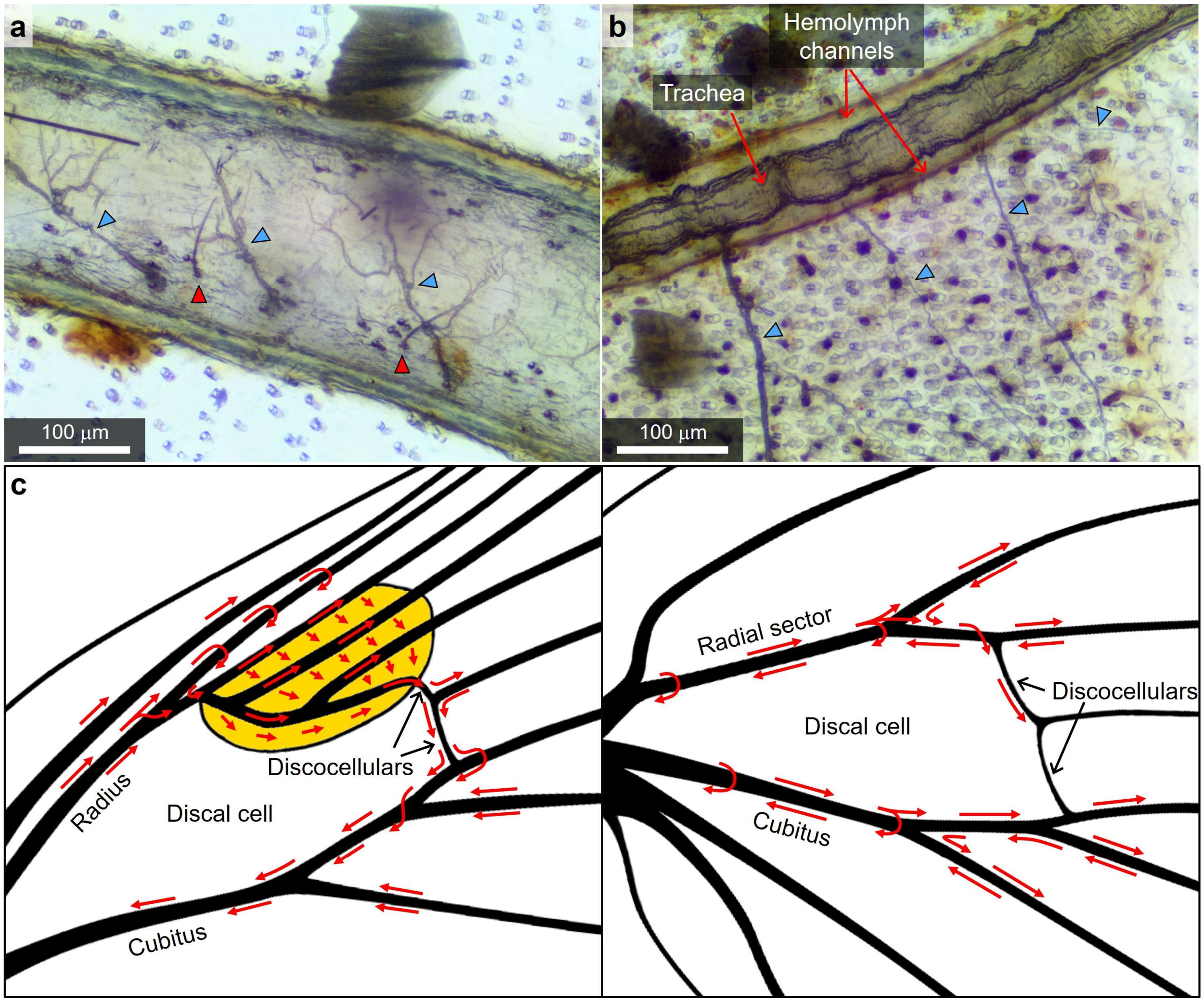

Supplementary Figure 5 | Internal structures and hemolymph flow in lycaenid butterfly wings. (a) Photo showing bristle sensilla (red arrowheads) and axons (blue arrowheads) in the wing vein of a male white-M hairstreak, *Parrhasius m-album*. (b) Photo showing what we believe to be axons (blue arrowheads) extending from a wing vein into the scent pad of a male *P. m-album*. (c) Observed pattern of hemolymph flow around the discal cells of the forewing and hindwing of a male hickory hairstreak, *Satyrium caryaevorus*. Yellow shaded region in the left panel indicates the scent pad.

Supplementary Note 5: Persistent hemolymph flow and thermo-sensing of the wing over butterflies' lifetime

We observed that for *V. cardui*, the hemolymph flow is sustained over the roughly three-week lifetime of adult butterflies, and that this activity shows no tendency of weakening (**Fig. 1g** of the main text). The hemolymph flow was monitored for individual butterflies of different ages that eclosed in the lab under controlled temperature and humidity levels. Each individual was mounted and tested only once.

The hemolymph flow speed was quantified by the dilation and contraction rates of the hemolymph channel, because the latter were found to be strongly correlated with the speed of hemocytes traveling in the hemolymph channels (**Supplementary Fig. 4c**). **Supplementary Fig.**

6a shows the average hemolymph channel dilation and contraction rates observed in 21 individuals at different ages, ranging from 3 days to more than 3 weeks after eclosion. The average dilation or contraction rate was calculated by dividing the total change of hemolymph channel width by the duration of each dilation or contraction event. One data point in **Supplementary Fig. 6a** stands for dilation or contraction rate averaged over 10 dilation or contraction events for one individual. Hemolymph flow remained steady throughout the observation period.

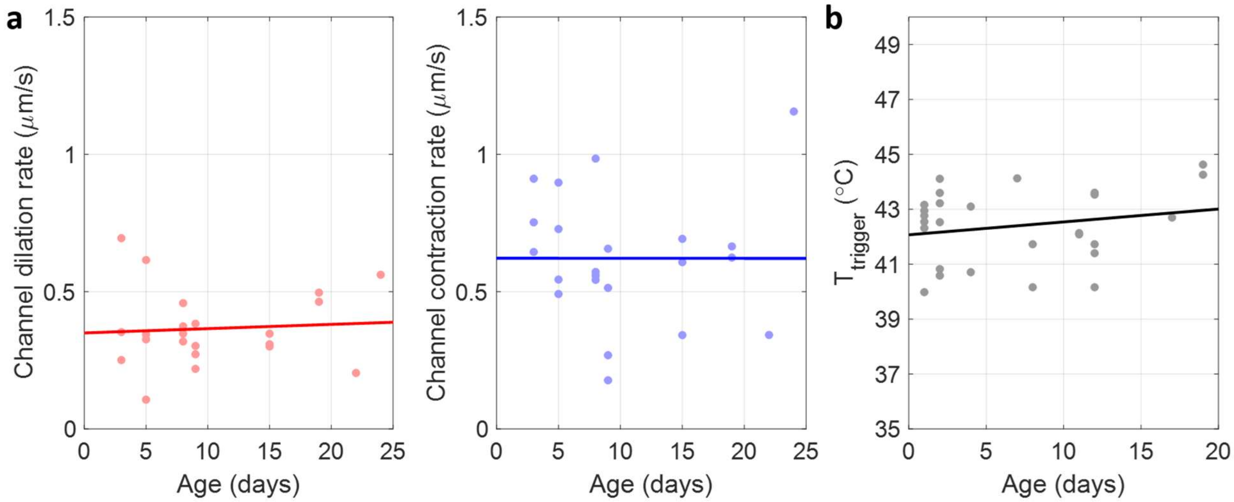

Supplementary Figure 6 | Hemolymph flow and thermal sensing do not vary with age in the wings of *Vanessa cardui*. (a) Average dilation (left) and contraction (right) rates of the hemolymph channel width as a function of age. One data point stands for dilation or contraction rate averaged over 10 dilation or contraction events for one individual. Linear fits to the data are also provided. (b) Threshold temperatures, $T_{trigger}$, triggering the displacement response measured on the forewings of *V. cardui* butterflies as a function of age. Linear fits indicate that age is not a significant predictor of any of these metrics (see text).

We calculated the Pearson's product-moment correlations between both the maximum and average contraction and dilation rates, respectively, and butterfly age. For the metric of contraction or dilation that was better correlated with age, we also analyzed its correlation to two metrics of body size (wing length and thorax length), controlling for multiple hypothesis correction using the technique of Benjamini and Hochberg[2] and found no significant relationships for any of these measures (**Supplementary Table 1**). The impact of age on the average hemolymph channel dilation and contraction rates is statistically non-significant ($r^2 = 0.0014$ and p-value = 0.8677 for the dilation rate; $r^2 = 0.000012$ and p-value = 0.9869 for the contraction rate).

We also measured the threshold temperatures ($T_{trigger}$) on the wings of *V. cardui* butterflies at different ages. We similarly analyzed its correlation to the two metrics of body size and found no significant relationship to any of these metrics (**Supplementary Table 1**). We found that the threshold temperatures persist within a small range over the course of about three weeks (**Supplementary Fig. 6b**). The resulting $r^2 = 0.0431$ and p-value = 0.2986 from a linear fit of the effect of age indicates that the effect of age on $T_{trigger}$ is not significant. Together, these results showing the continuous operation of the thermal sensory system of butterfly wings suggest that hemolymph flow is maintained over the butterfly's entire lifespan.

Supplementary Table 1 Age and body size have no effect on dilation/contraction rates of hemolymph channels and $T_{trigger}$ in *V. cardui* butterflies

Dependent variable	Independent variable	Test*	Statistic	*p*-value
Dilation rate, maximum	Age	PPMC	-0.56535	0.577827
Dilation rate, maximum	Wing length	PPMC	-0.10099	0.920518
Dilation rate, maximum	Thorax length	PPMC	2.179439	0.040828
Contraction rate, maximum	Age	PPMC	-0.45915	0.650843
Contraction rate, maximum	Wing length	PPMC	-0.37518	0.711292
Contraction rate, maximum	Thorax length	PPMC	0.504565	0.619118
$T_{trigger}$**	Age	PPMC	1.0614	0.2986
$T_{trigger}$**	Wing length	PPMC	-1.4831	0.1506
$T_{trigger}$**	Thorax length	PPMC	1.4555	0.158

*PPMC: Pearson's product-moment correlation.
**In the analysis of the impact of age and two metrics of body size (wing length and thorax length) on the threshold temperature, $T_{trigger}$, a maximal model containing all of these factors was overfit, as determined by a small adjusted and predicted R-squared value, so we employed hierarchical factor level reduction. In all of the models, these variables and their interactions are never significant, explaining relatively little of the variation in $T_{trigger}$, and have correspondingly poor adjusted and predicted R-square values. We then analyzed the correlations between these predictors and the $T_{trigger}$ individually using Pearson's product-moment correlations, in the case of age and the two metrics of body size, finding that none of these variables is significantly correlated with the $T_{trigger}$.

Although we did not perform systematic studies of hemolymph flow as a function of age in other butterflies, we conducted multiple experiments (including laser-induced local heating experiments and basking experiments) on lycaenid butterflies (including *P. m-album* and *S. caryaevorus*, all caught from the field), and these tests lasted about one week for each individual. We observed that these butterflies reacted to overheating on their wings throughout the testing period (**Figs. 6** and **7** in the main text). This continuous functioning of the thermal sensory system implies that the hemolymph flow in the wing veins of these butterflies, similar to *V. cardui*, has also been maintained over the testing period.

The butterflies used to measure the $T_{trigger}$ (**Supplementary Table 7**) were presumably at different ages when they were randomly caught from the field. We observed that, as long as their wings appear intact, the butterflies can sense overheating on their wing veins in the laser-induced local heating experiment. That wing sensory capabilities are maintained in all the adult butterflies surveyed in this study strongly suggests the hemolymph required to sustain the living tissue continues to flow throughout the adult lifespan in all of these species.

Supplementary Note 6: Impact of overheating on the function of butterfly wings
Temperature is a critical factor for living organisms. Cells grow and function optimally in a finite temperature window, and malfunction and die when the temperature rises above a certain value. The impact of heat stress on cells has been intensively studied. For example, lethal temperatures ranging from 42-56°C for a number of Mediterranean ant species have been reported[3]. Cell death can be induced by heat that denatures protein structures and decreases protein stability. Previous studies have explored the mechanism of temperature-induced protein denaturation, resulting in cellular collapse, and have reported that essential proteins in *E. coli* start to melt at 40°C[4].

We conducted a series of experiments on adult *Vanessa cardui* to investigate the impact of overheating on the function of butterfly wings. This work was necessary in order to determine whether overheating of the wing itself (and not the entire insect) could have adaptive consequences for butterflies; however, given the nature of the experiment, we made every effort to use only the minimum number of specimens necessary to measure potential effects.

A living butterfly was first contained in an arena where it could walk freely. A stimulus laser beam produced by a supercontinuum light source (λ = 400−1700 nm) with a diameter of ~3 mm was applied to the first junction of the radial veins on the forewing (**Supplementary Fig. 7a**), and the threshold temperature ($T_{trigger}$), i.e., the point at which the insect begins to turn in order to move the wing out of the beam of the laser, was recorded. The butterfly was then removed from the container and secured with its wings closed. A heating laser beam generated by the supercontinuum source with a larger diameter of ~10 mm was applied to the same location on the forewing to heat up the area uniformly (**Supplementary Fig. 7a**). During the exposure, the laser power was controlled to maintain a constant temperature, and each exposure lasted for 3 minutes. After the exposure, the butterfly was released and placed back into the container where it could walk freely again. After a few minutes to allow the individual to re-equilibrate, its $T_{trigger}$ was measured for a second time at the same location on the forewing. We repeated the above procedure, sequentially heating and measuring the $T_{trigger}$ for up to 10 treatments, or until the butterfly stopped responding to the laser beam. A total of 18 butterflies were randomly assigned to one of three different heating temperatures: 42°C, 46°C, and 52°C. These 'high temperature' treatments were selected based on temperatures that wing veins and membrane areas were commonly observed to reach under full sun conditions (**Fig. 5** of the main text).

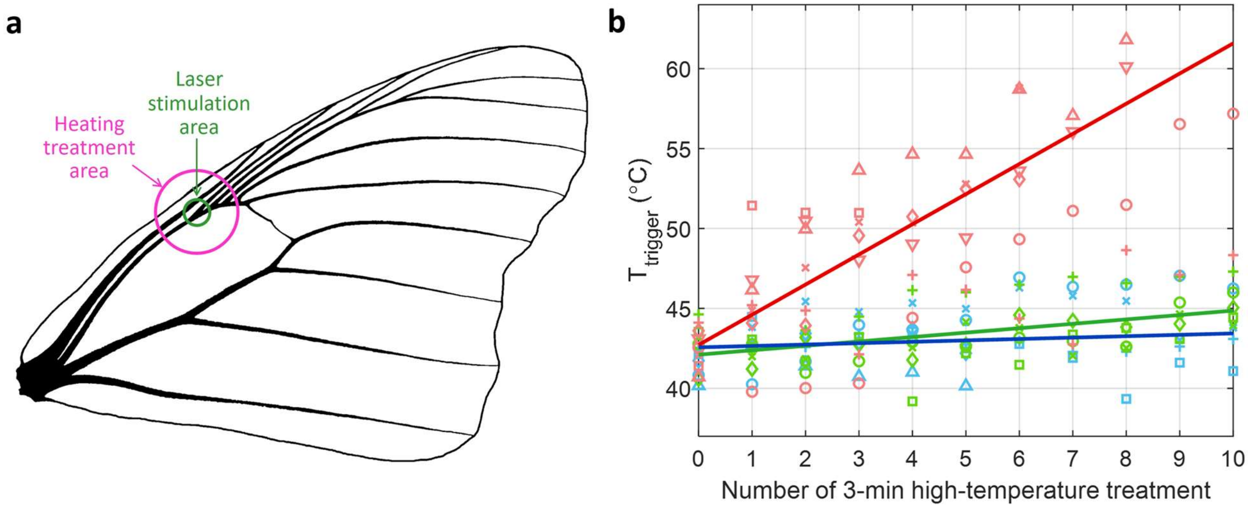

Supplementary Figure 7 | Impact of overheating on sensory function of butterfly wings. (a) Schematic showing the venation of the *Vanessa cardui* forewing. The large, pink circle indicates the size and location of the heating laser spot that heated the local region to one of the three elevated temperatures of 42°C, 46°C, and 52°C; the small, green circle indicates the size and location of the laser spot used in the laser-induced local heating experiments. (b) Threshold temperature ($T_{trigger}$) of *V. cardui* butterflies as a function of number of 3-min exposures to three heating temperatures. The data points present the averaged threshold temperature of each individual, exposed to different heating temperatures (blue: 42°C, n = 6; green: 46°C, n = 5; red: 52°C, n = 7). Different symbols represent different individuals. The solid lines provide linear fits to the measured data.

We observed that butterflies exposed to the two lower heating temperatures showed only minimal increases in $T_{trigger}$ per treatment event. To analyze the effect of treatment on $T_{trigger}$, we first fit a maximal mixed effects linear model that also accounted for the potentially confounding effects of sex and body size. Following hierarchical factor reduction, only treatment, event, and their interaction remained as significant predictors of $T_{trigger}$. To formally test the significance of the contributions of these effects to the model, we used hierarchical parametric bootstrapping (see Methods section for a detailed description of this analysis). This analysis showed that the effects of sequential treatment events, and its interaction with treatment are highly significant ($T = 24.4$, $p = 0.0002$ and $T = 9.97$, $p = 0.001$, respectively), while the effect of treatment alone (i.e., the effect of treatment on the first event) is not. The lower temperature treatments increase $T_{trigger}$ only 0.079ºC and 0.196ºC per treatment-event for the effect of repeated exposure to 42ºC and 46ºC, respectively, whereas the effect of treatment was much larger (1.719ºC per treatment-event) for those butterflies exposed to the high heating temperature of 52ºC (**Supplementary Fig. 7b**). Only two of the seven individuals tested at the high heating temperature still exhibited displacement response after 10 treatments. This experiment shows that an ecologically realistic repeated heat stress can drastically alter the functionality of thermal sensors (and likely other types of living cells) in the wing, likely due to malfunction following overheating, and indicates that strategies to prevent overheating are important for the function of butterfly wings.

Supplementary Note 7: Effects of hemolymph flow on wing temperature

We carried out the following analysis to investigate the possible contribution of hemolymph flow to the temperature distribution in butterfly wings. We assume that a 25-mm long (L) vein on a *V. cardui*'s wing is exposed to full sun (I =1000 W/m^2). Based on measurements of butterfly temperatures, we assume that the thoracic temperature, and thus the temperature of the hemolymph flowing from the thorax into the wing, is $T_t = 30°\mathrm{C}$. We further assume that the equilibrium temperatures of the vein and the adjacent membranous regions reach $T_v = 40°\mathrm{C}$ and $T_m = 50°\mathrm{C}$, respectively, and that the width of the temperature transition zone is 300 µm (i.e., a temperature gradient of $\nabla T = 0.033°\mathrm{C}/\mu\mathrm{m}$ between the vein and the membranous regions). Considering the diameter of the vein, $D = 195$ µm, the membrane thickness, $t = 11$ µm, the cross-sectional area of the hemolymph channels, $A = 1850\ \mu\mathrm{m}^2$ (**Supplementary Fig. 4b**), the peak flow speed of hemolymph from the thorax into the wing, $v = 1\ \mathrm{mm} \cdot sec^{-1}$ (**Fig. 1g** of the main text), the specific heat capacity of hemolymph, $C_v = 4.18 \times 10^{-12}\ \mathrm{J} \cdot \mu\mathrm{m}^{-3} \cdot °\mathrm{C}^{-1}$ (i.e., water at 25°C), and the thermal conductivity (k) of chitin (material composing wing membranes), $0.25\ \mathrm{W} \cdot \mathrm{m}^{-1} \cdot \mathrm{K}^{-1}$, we can estimate the heat fluxes toward the vein: The radiative power absorbed by the vein, $P = \alpha I \times L \times D = 2.925\ \mathrm{mW}$, where α~0.6 is the solar absorptivity; The heat transferred by conduction from the membranous regions (50°C) to the vein (40°C), $dQ_c/dt = kL \times t\nabla T = 2.291$ mW. However, the rate at which the wing vein can be cooled down by the outflux of cool hemolymph from the thorax to the wing is $dQ_h/dt = C_v A v(T_v - T_t) = 77.3$ µW, which is ~1.5% of the combined contribution of radiative and conductive heat transfer. Therefore, we can conclude that the effect of hemolymph flow on cooling butterfly wings is insignificant. A similar analysis reveals that the influx of warm hemolymph from the wing to the thorax has negligible effects on increasing the thoracic temperature compared to the contribution from direct absorption of sunlight.

We conducted measurements comparing the thermal emissivities of the wing of an individual *Danaus plexippus* (the monarch butterfly) while it was alive and after it was dead and dehydrated. **Supplementary Fig. 8a** shows the emissivity distributions of a region of the butterfly

wing (containing a Y-branch of veins and surrounding wing membranes) at a few mid-infrared wavelengths. A comparison of the emissivity distributions when the butterfly was alive and 3 days after the butterfly was dead and fully dehydrated shows that the change in emissivity is minimal over the tested region (**Supplementary Fig. 8a**). **Supplementary Fig. 8b** shows the spectral difference in emissivity, averaged over the tested region of 3 mm × 3 mm. The results indicate that the impact of the variation of hemolymph volume (i.e., the extreme cases of presence and absence of hemolymph) on the emissivity of butterfly wings is negligible and thus the radiative cooling properties of butterfly wings can be considered as invariant.

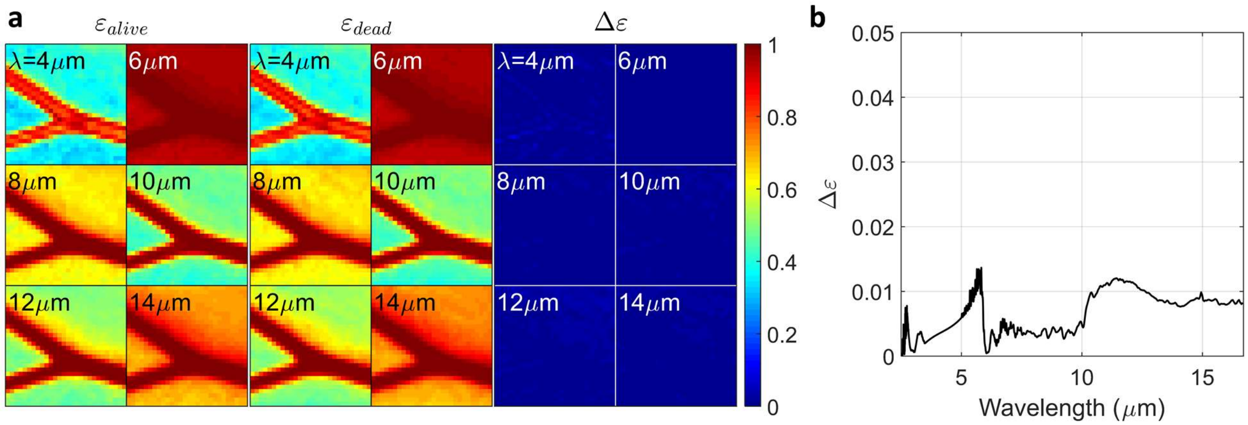

Supplementary Figure 8 | Impact of the presence of hemolymph on the thermal emissivity is negligible. (a) Left: Emissivity distributions over a wing region at a few mid-infrared wavelengths for a live *Danaus plexippus* butterfly. Middle: Emissivity distributions over the same wing region when the butterfly was dead and dehydrated. Right: Differential emissivity distributions. (b) Spectral difference in emissivity average over the region shown in (a) before and the after the butterfly was dead.

Supplementary Note 8: Morphological and spectroscopic studies of wing scales of lycaenid butterflies

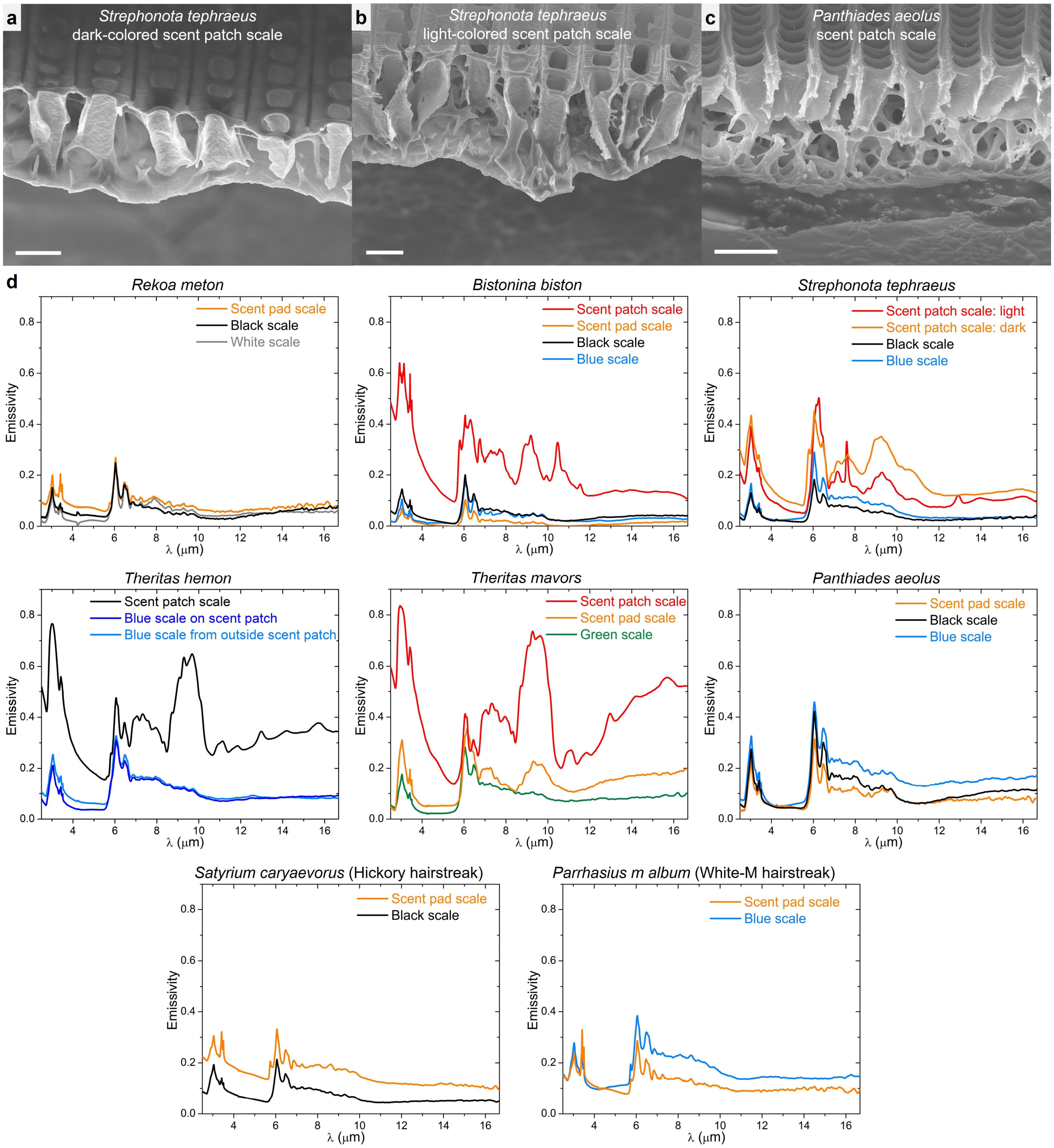

Supplementary Figure 9 | Wing scale nanostructures and measured thermal emissivity spectra of individual wing scales. (a) *Stephonota tephraeus*, dark-colored scent patch. (b) *S. tephraeus*, light-colored scent patch. (c) *Panthiades aeolus*, scent patch. Scale bars in (a)-(c): 2 μm. Notice that these scent patch scales are often associated with interior tubular structures similar to those found in the *Bistonina biston* scent patch scales (**Fig. 4d** of the main text). (d) Enhanced thermal emissivity was observed in all scent patch scales. The individual scent patch scales of *Theritas hemon* and *T. mavors* have the highest integrated thermal emissivity of ~0.38. Males of *S. tephraeus* have two scent patches and both have moderately enhanced emissivity (0.14 and 0.20

for the light and dark colored scent patch scales, respectively). All other types of scales have comparatively small thermal emissivity.

Supplementary Note 9: Stacking photography of wing scales of lycaenid butterflies

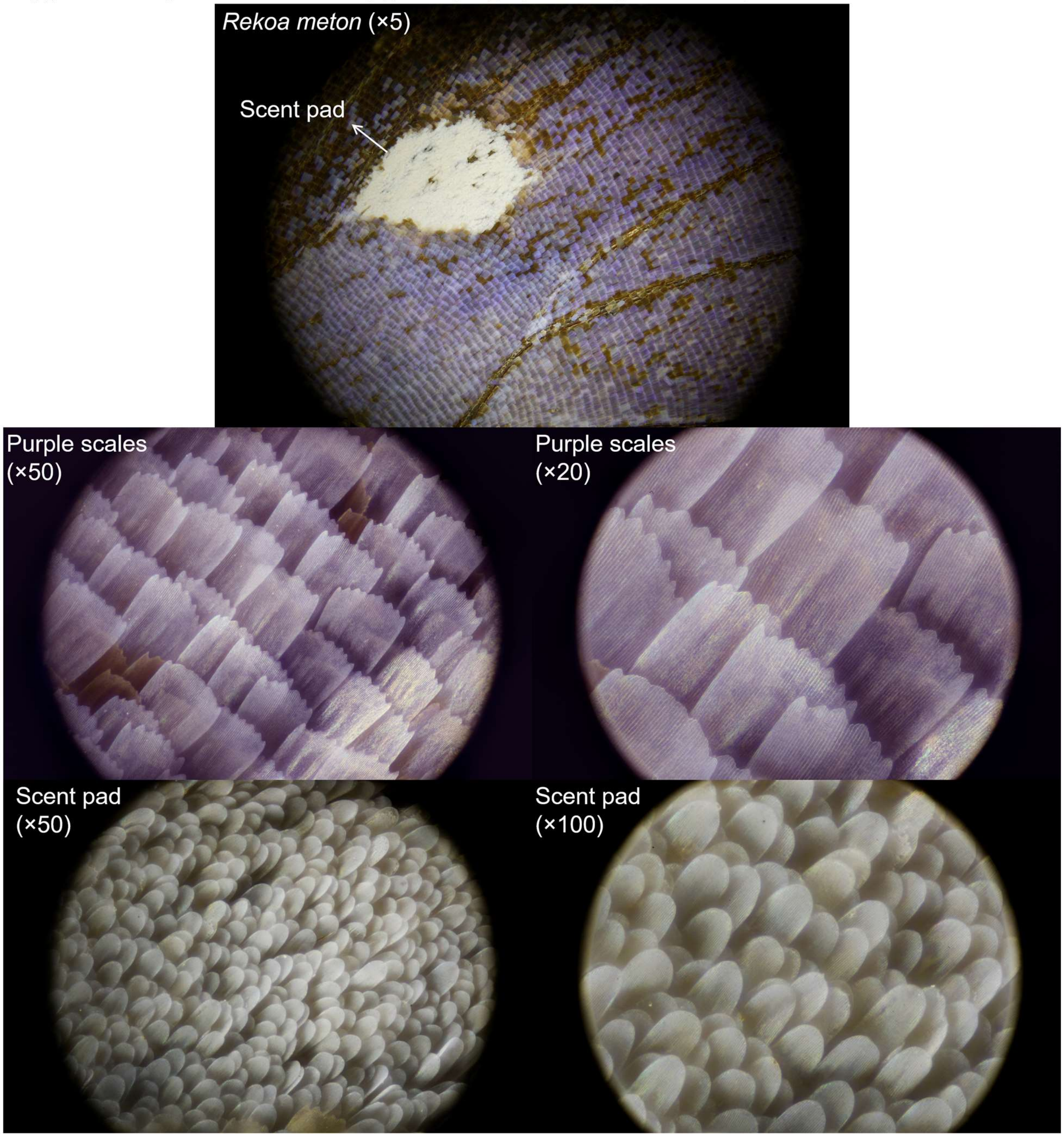

Supplementary Figure 10 | Wing scales of a male *Rekoa meton*.

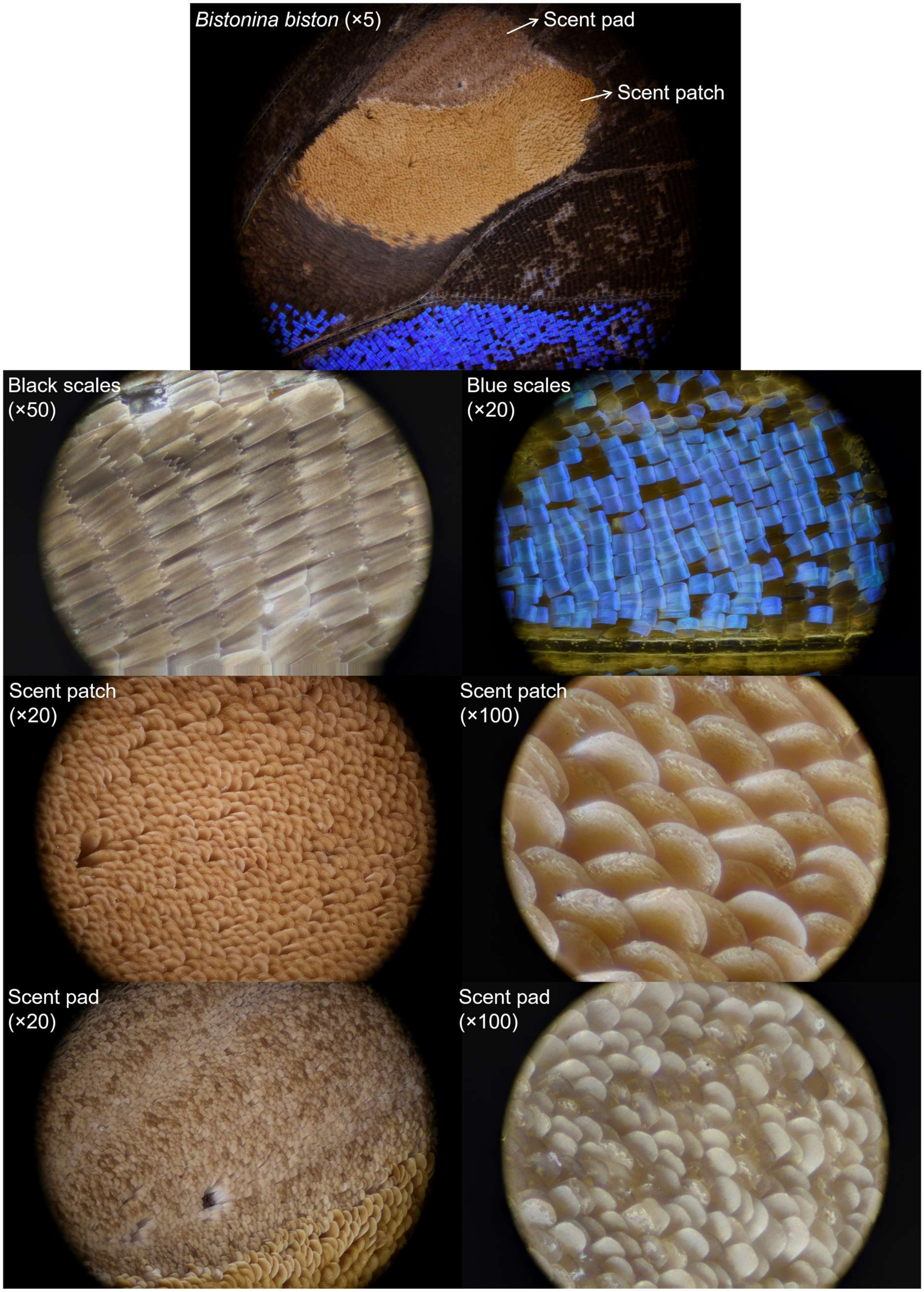

Supplementary Figure 11 | Wing scales of a male *Bistonina biston*.

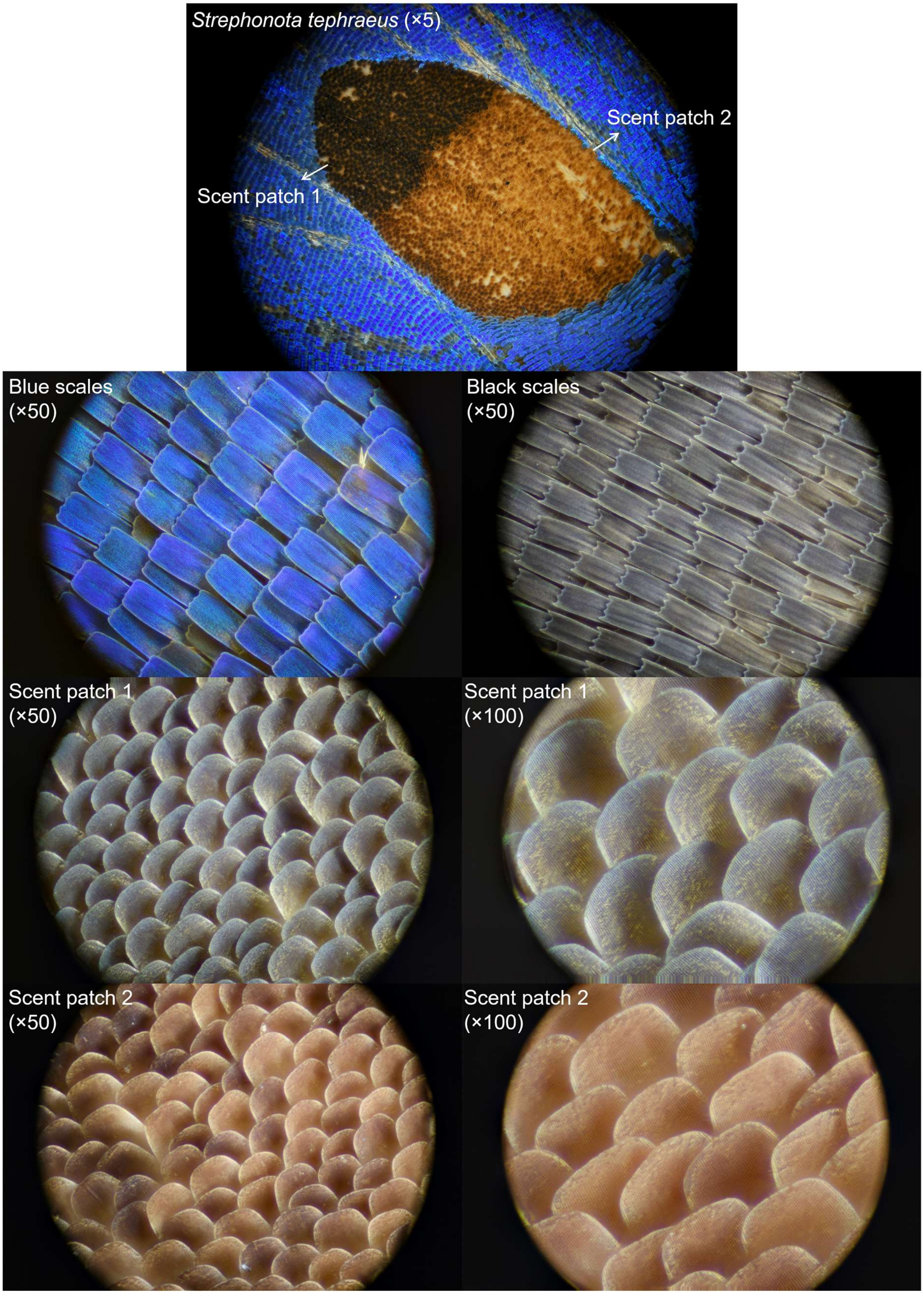

Supplementary Figure 12 | Wing scales of a male *Strephonota tephraeus*.

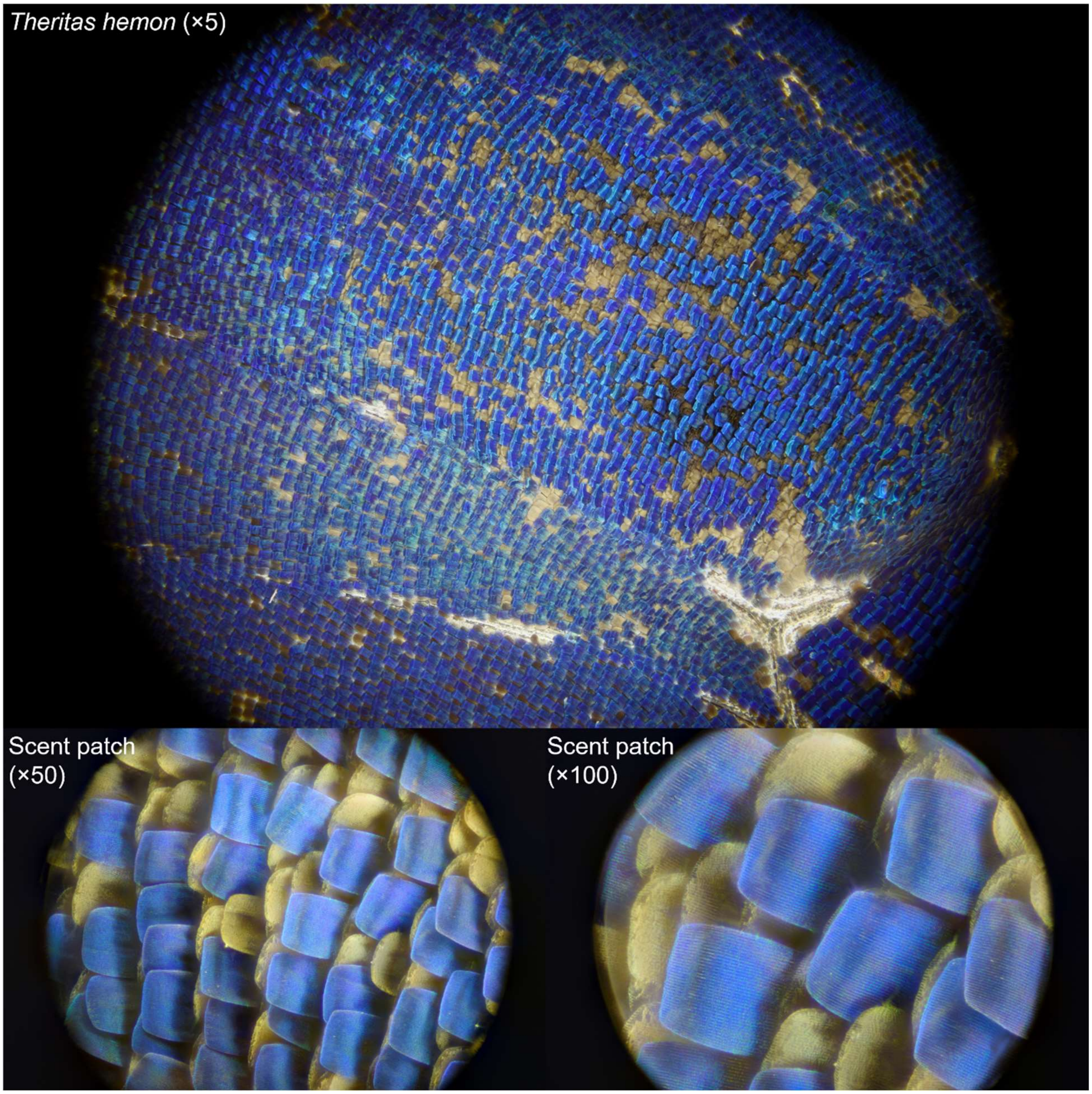

Supplementary Figure 13 | Wing scales of a male *Theritas hemon*.

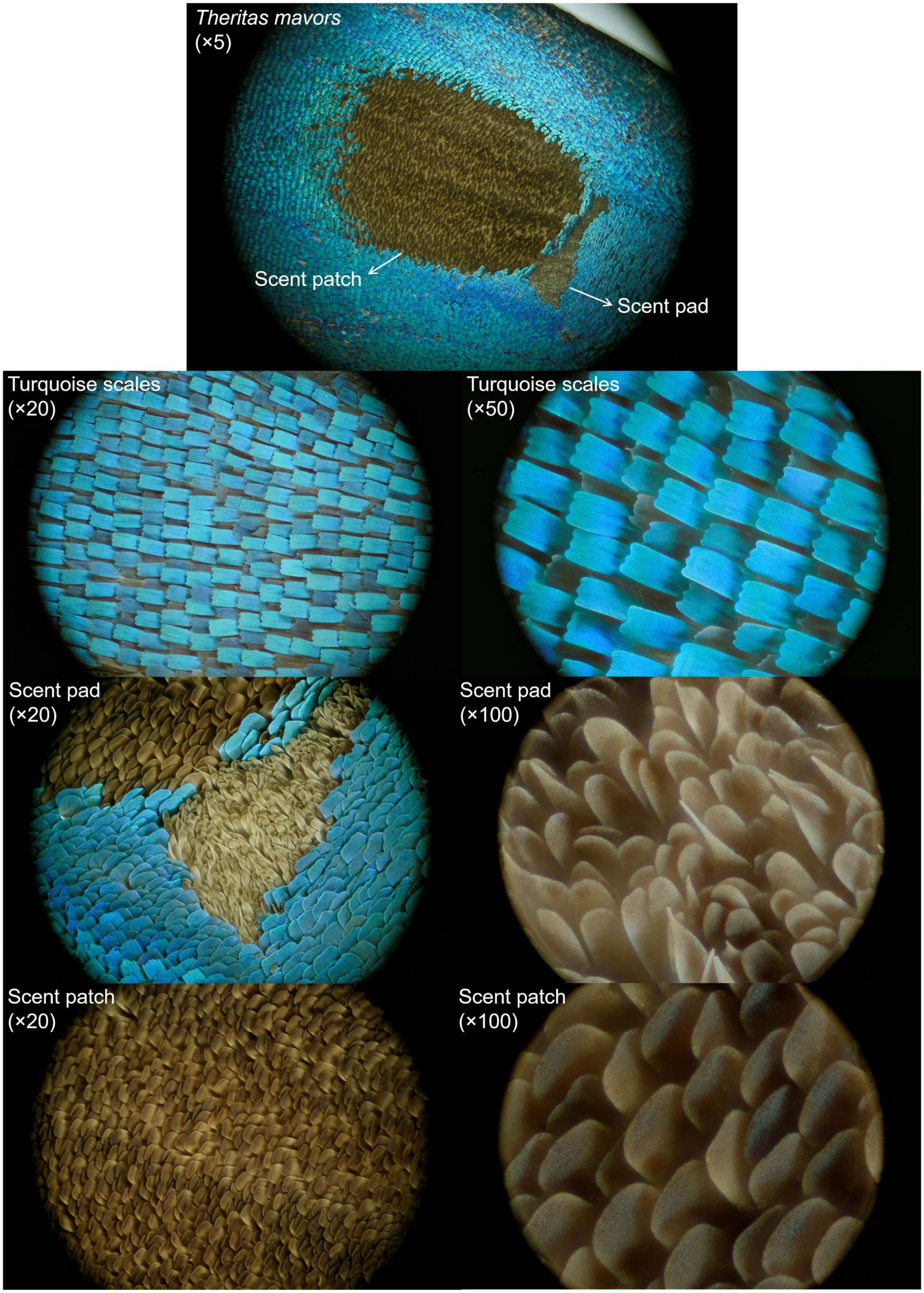

Supplementary Figure 14 | Wing scales of a male *Theritas mavors*.

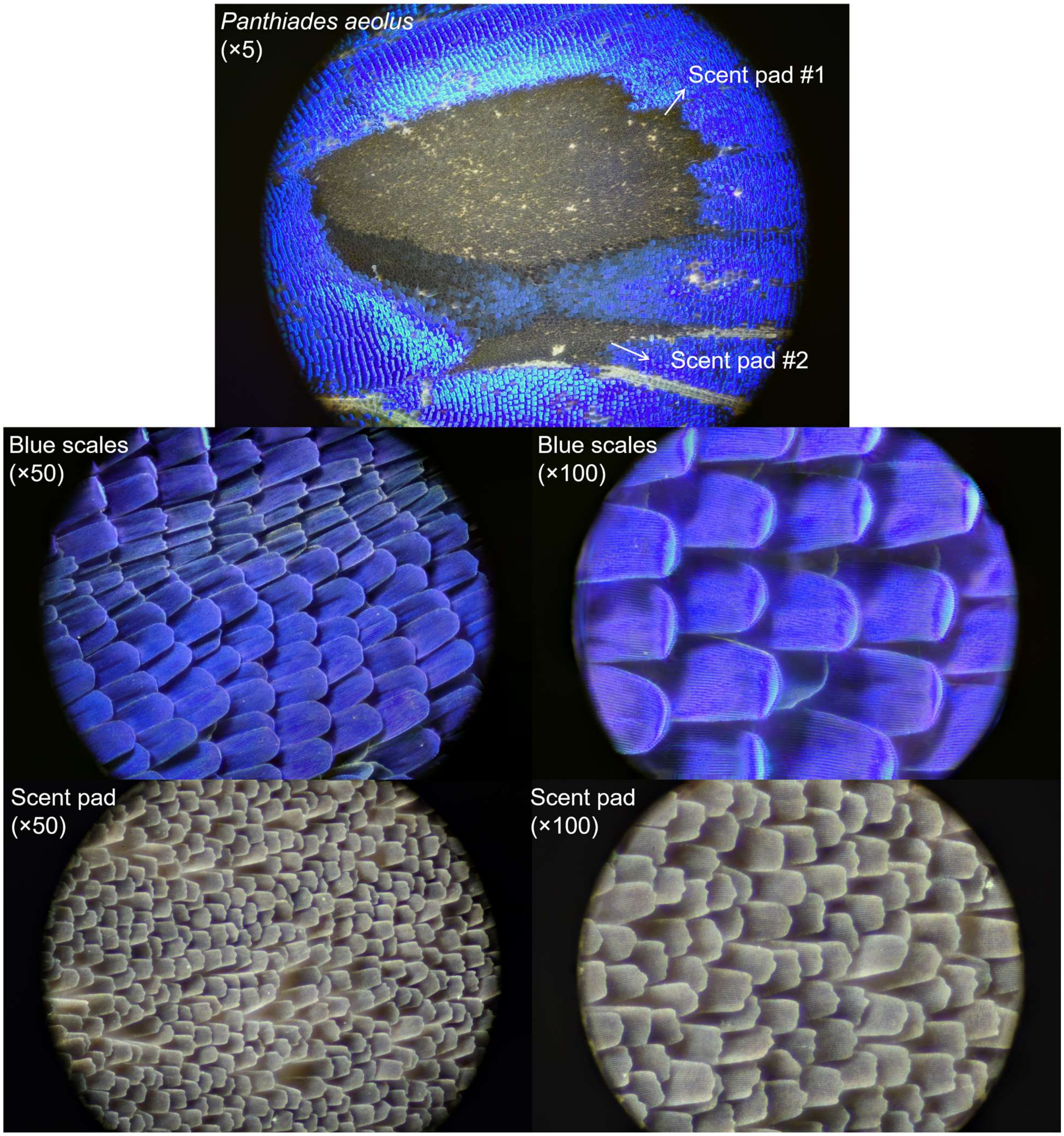

Supplementary Figure 15 | Wing scales of a male *Panthiades aeolus*.

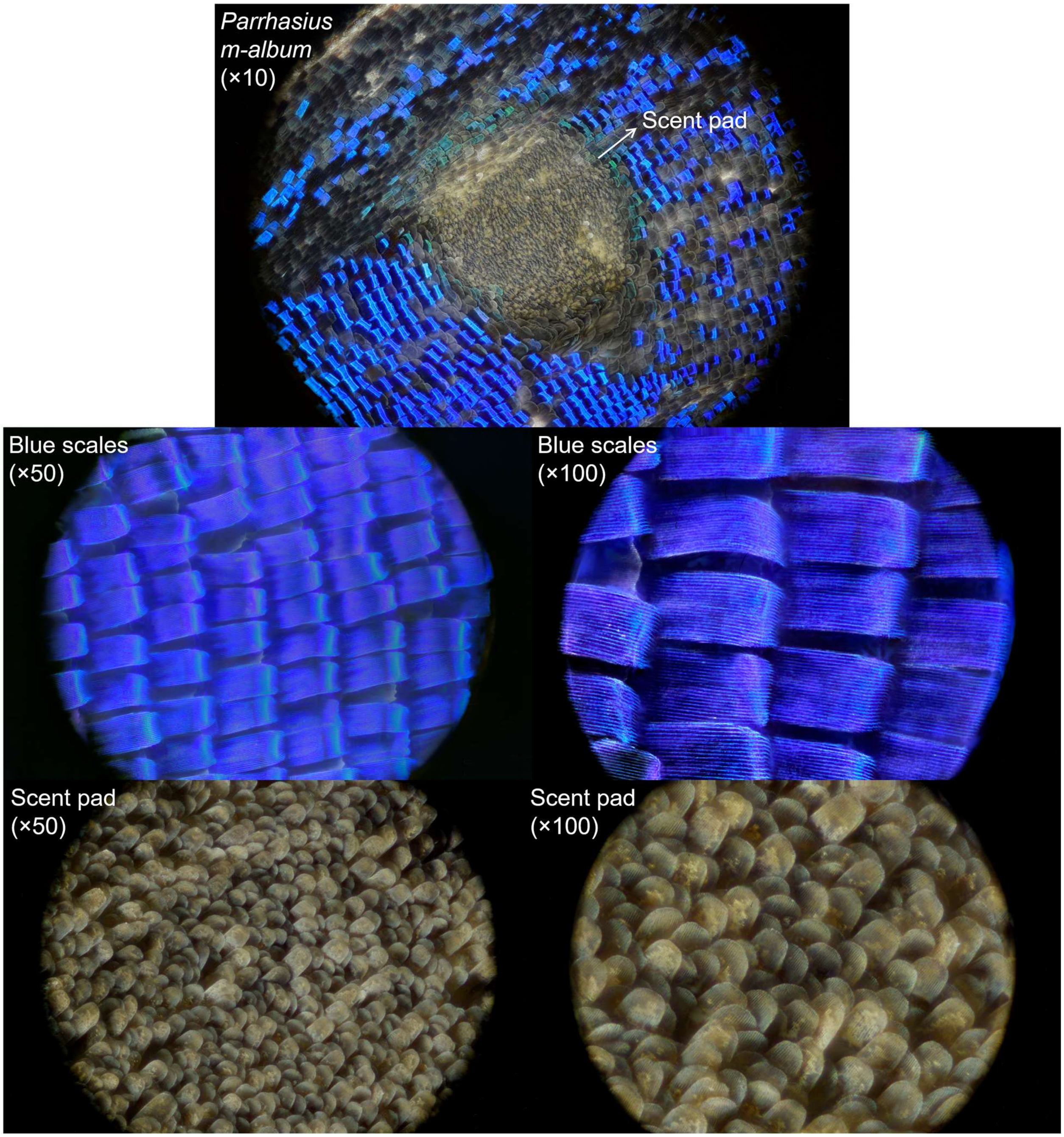

Supplementary Figure 16 | Wing scales of a male *Parrhasius m-album*.

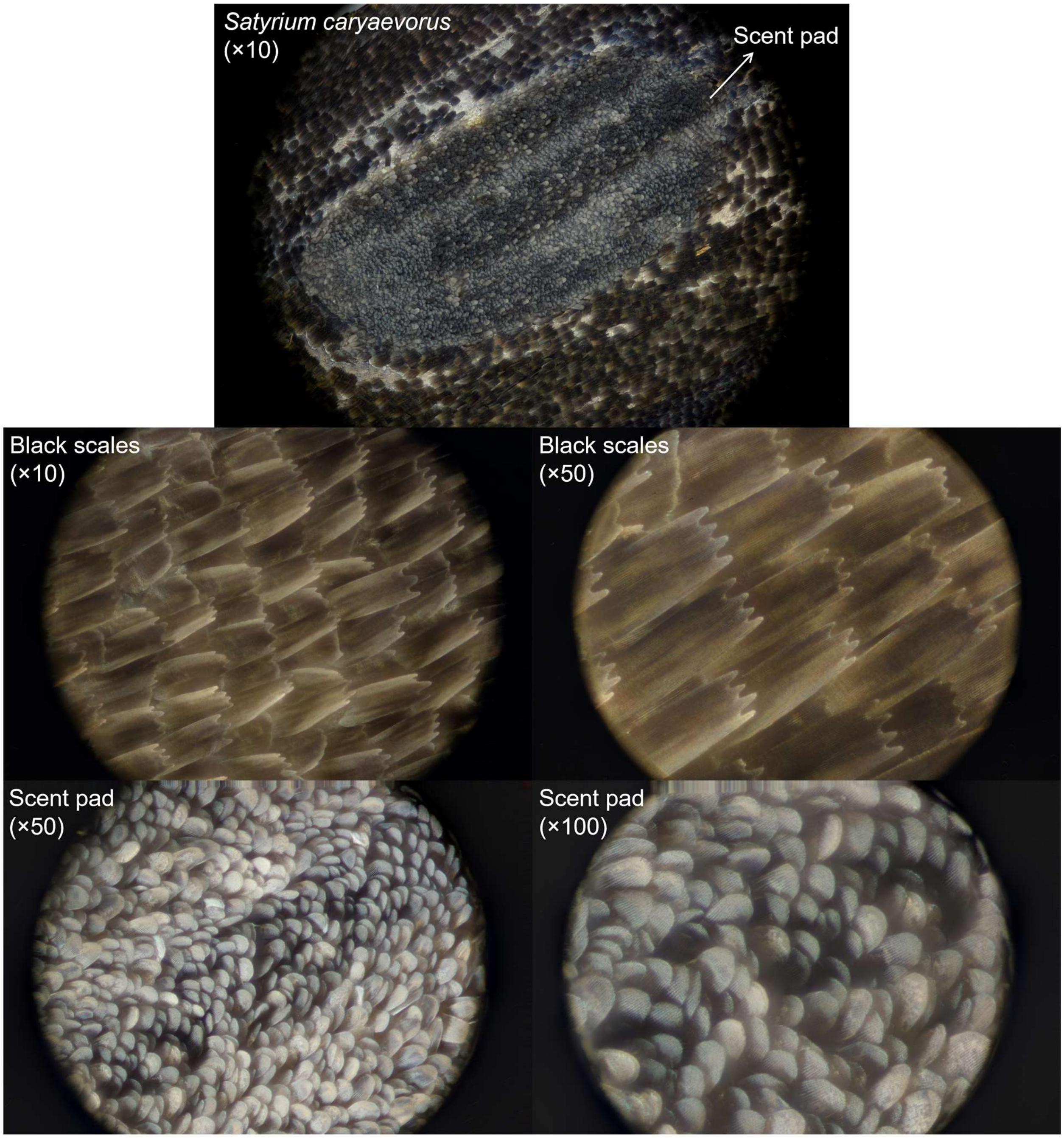

Supplementary Figure 17 | Wing scales of a male *Satyrium caryaevorus*.

Supplementary Note 10: Infrared hyperspectral imaging of butterfly wings

Measured emissivity and transmissivity maps of 7 lycaenid butterflies are shown in **Supplementary Figs. 18–31**. Enhanced thermal emissivity at scent patches, scent pads, and wing veins was observed in over 50 species of butterflies, a list of which is provided in **Supplementary Tables 6** and **7**.

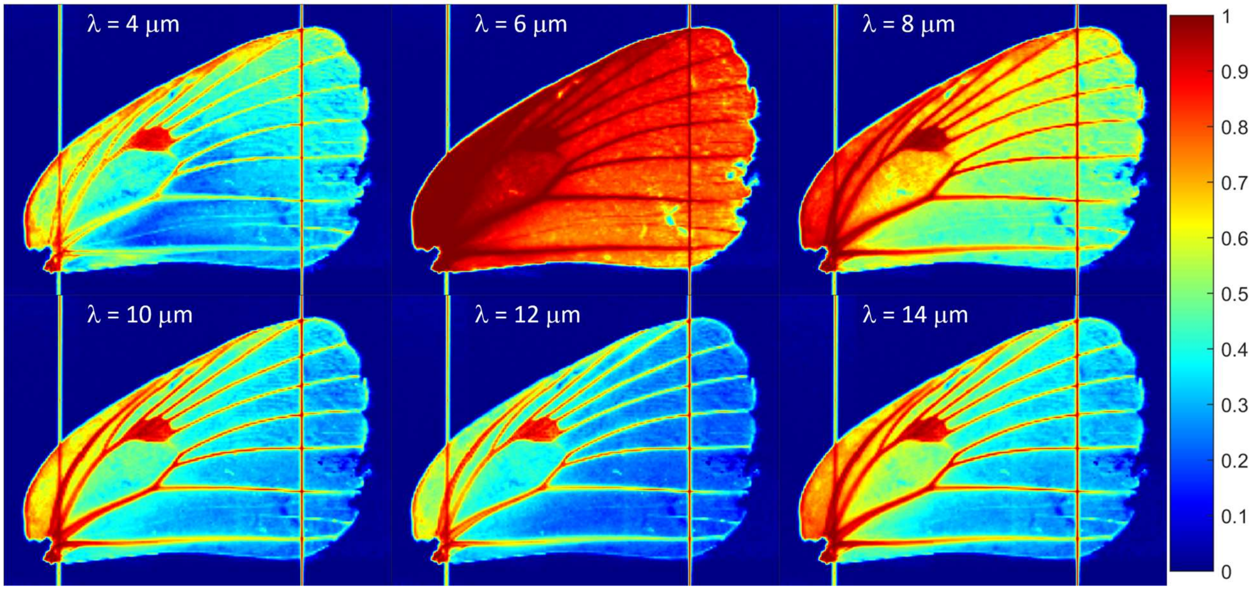

Supplementary Figure 18 | Emissivity distributions on the forewing of a male *Rekoa meton*.

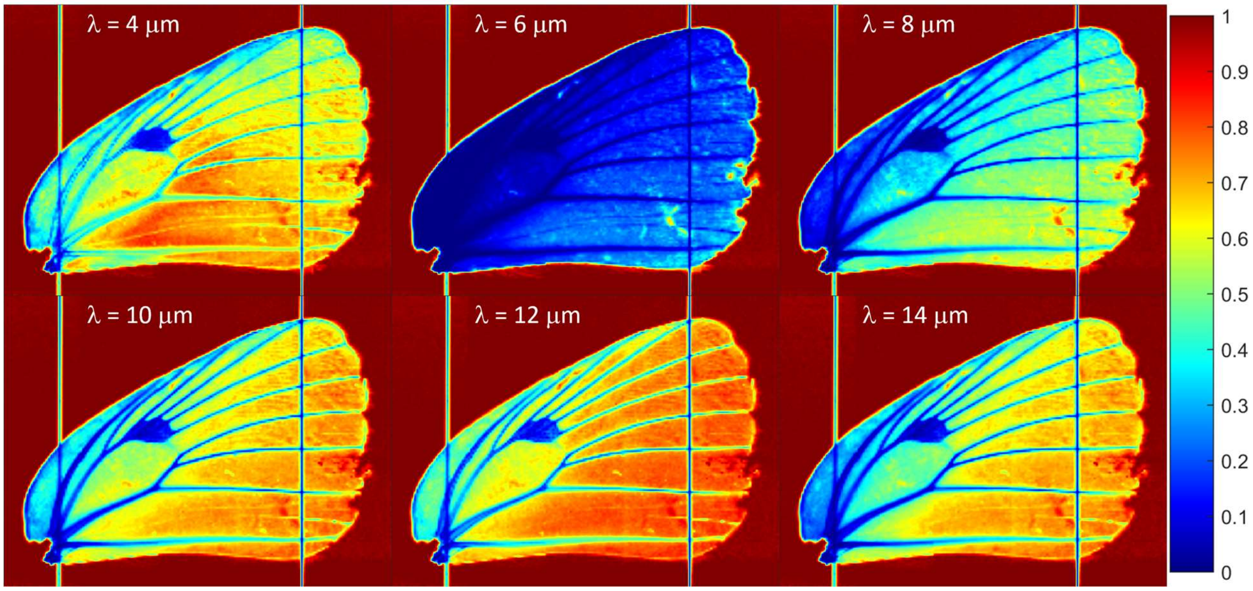

Supplementary Figure 19 | Transmissivity distributions on the forewing of a male *Rekoa meton*.

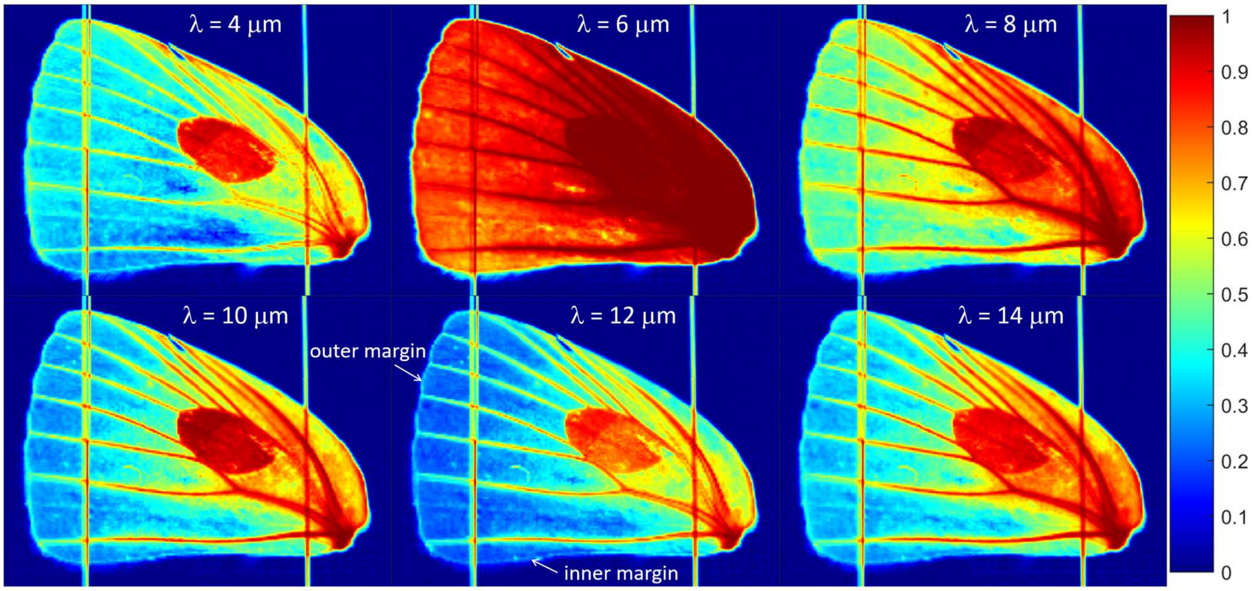

Supplementary Figure 20 | Emissivity distributions on the forewing of a male *Strephonota tephraeus*. Notice the elevated emissivity along the outer and inner margins.

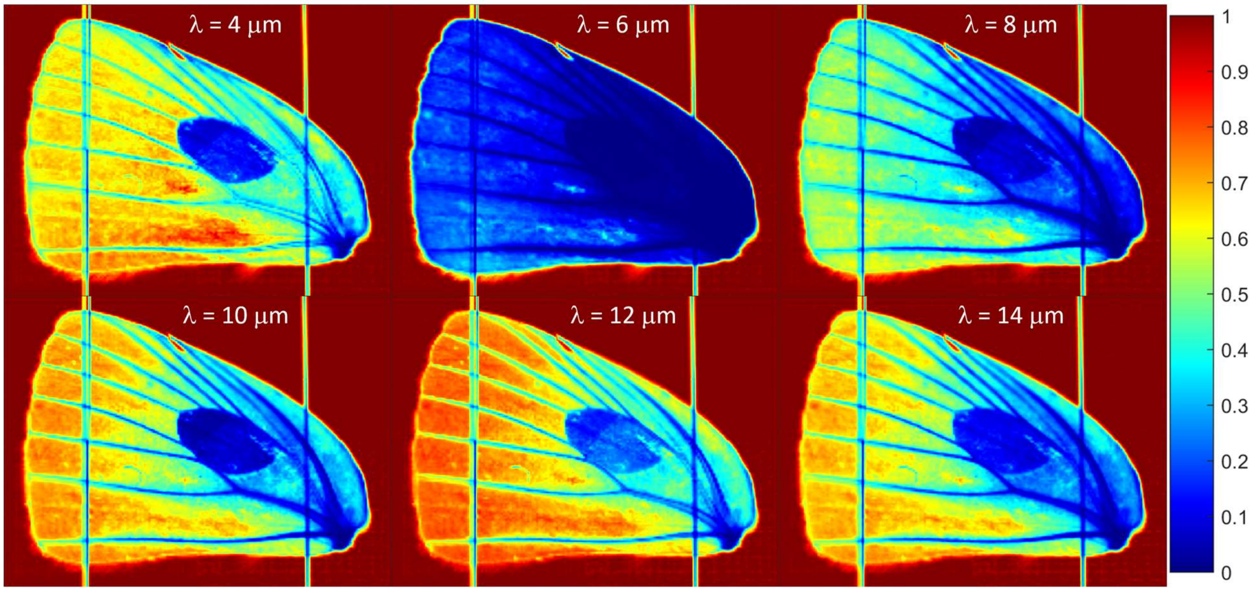

Supplementary Figure 21 | Transmissivity distributions on the forewing of a male *Strephonota tephraeus*.

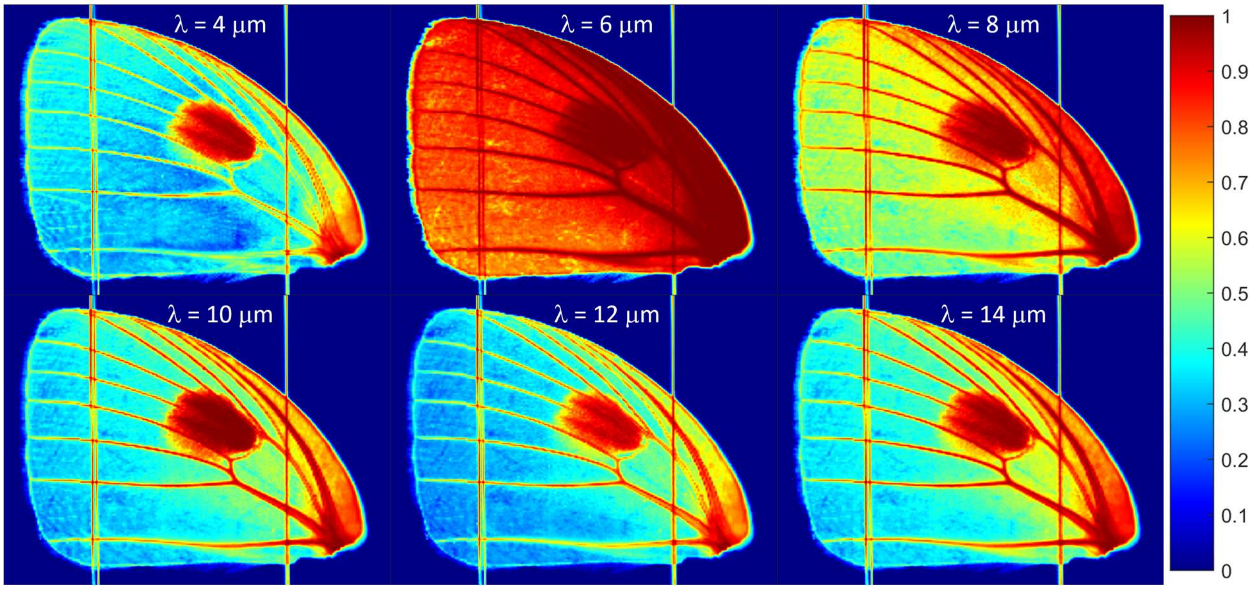

Supplementary Figure 22 | Emissivity distributions on the forewing of a male *Theritas hemon*.

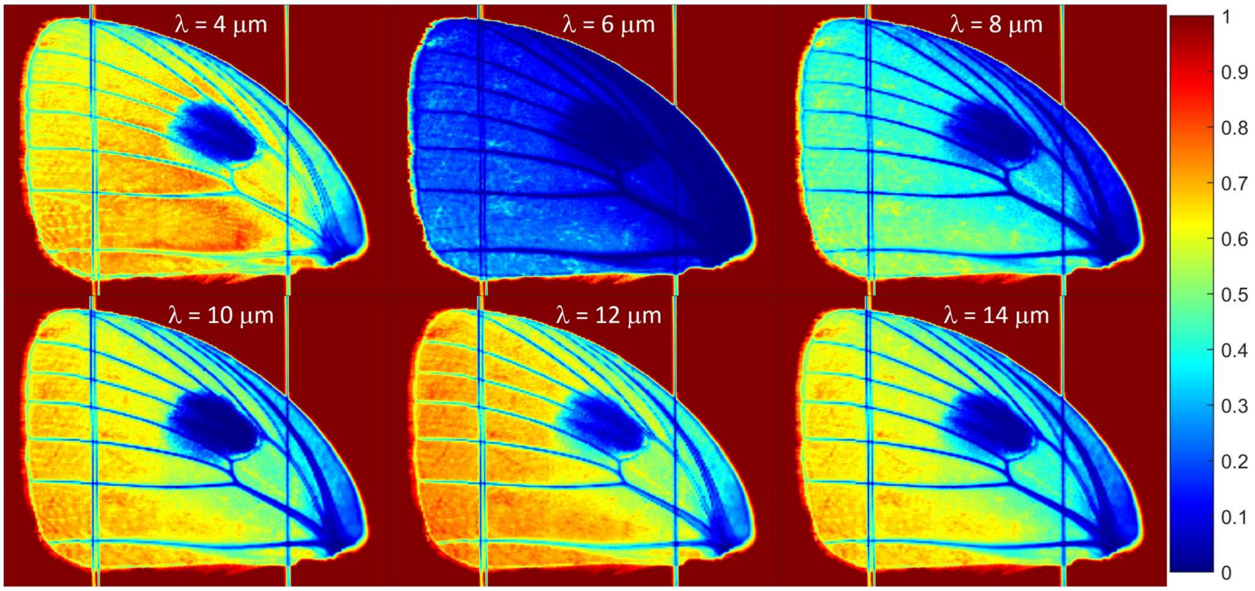

Supplementary Figure 23 | Transmissivity distributions on the forewing of a male *Theritas hemon*.

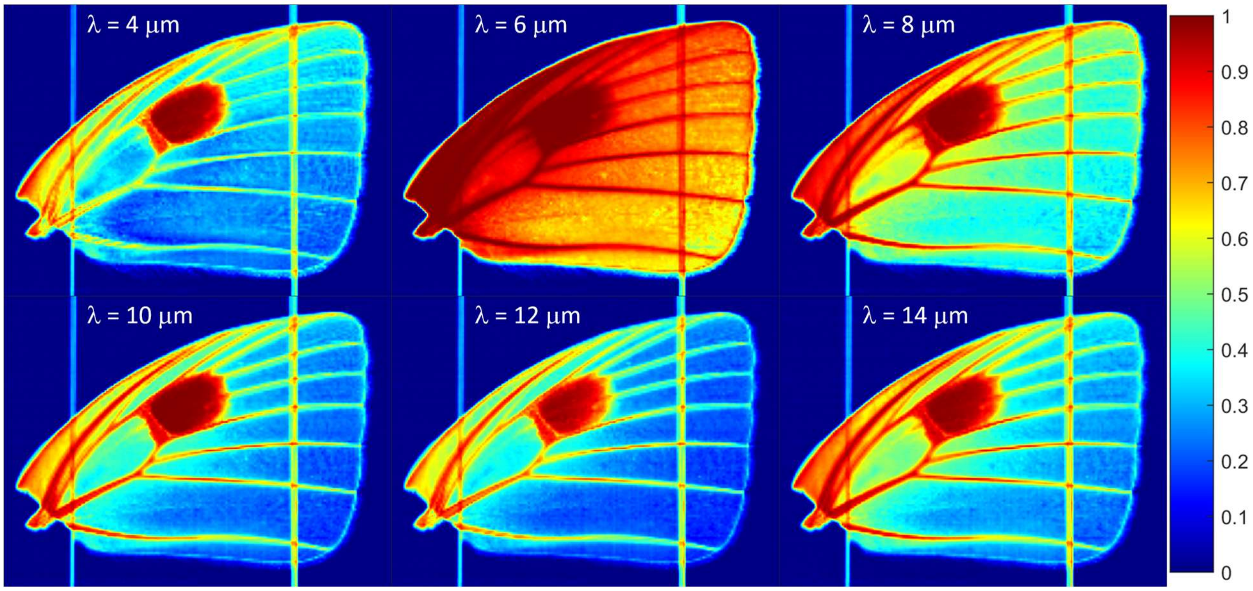

Supplementary Figure 24 | Emissivity distributions on the forewing of a male *Theritas mavors*.

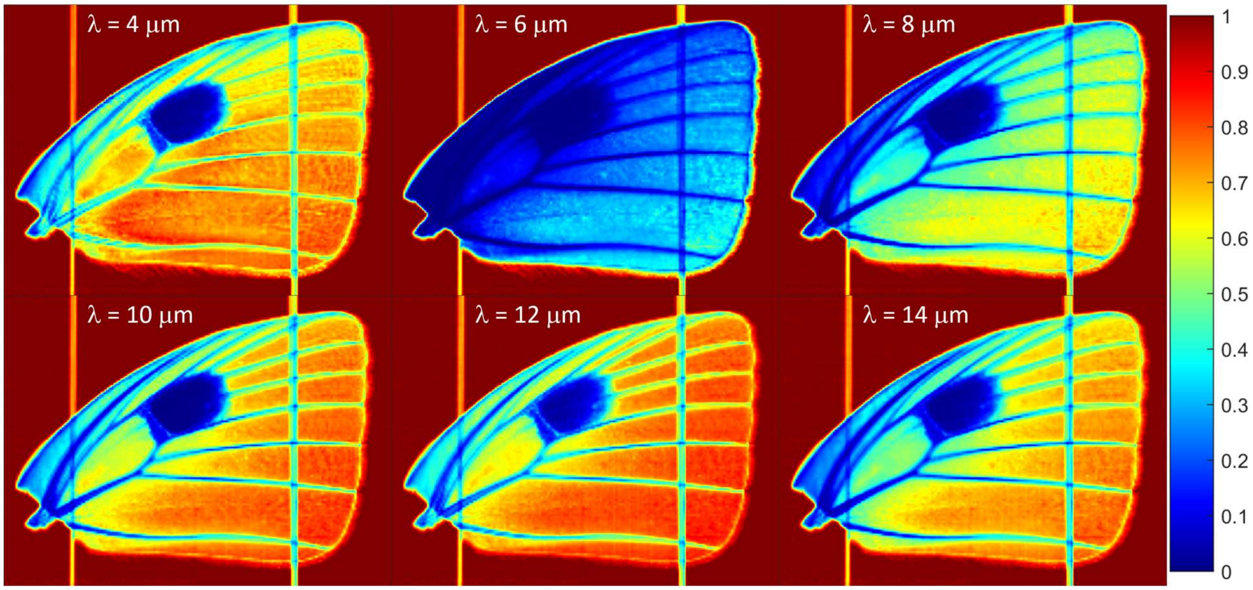

Supplementary Figure 25 | Transmissivity distributions on the forewing of a male *Theritas mavors*.

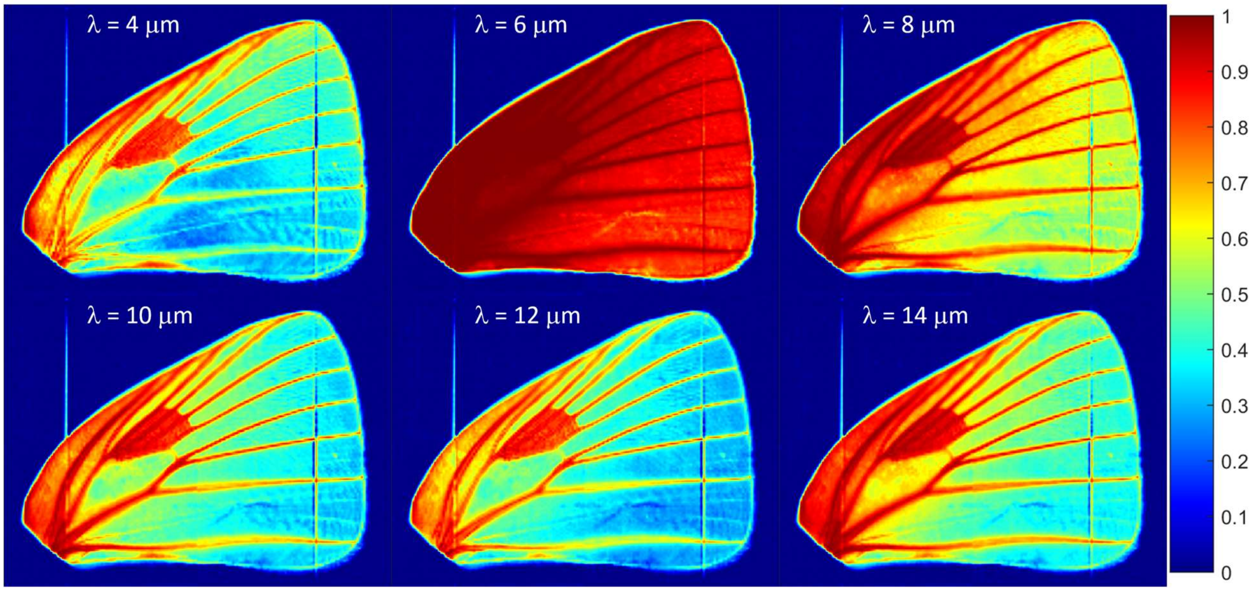

Supplementary Figure 26 | Emissivity distributions on the forewing of a male *Panthiades aeolus*.

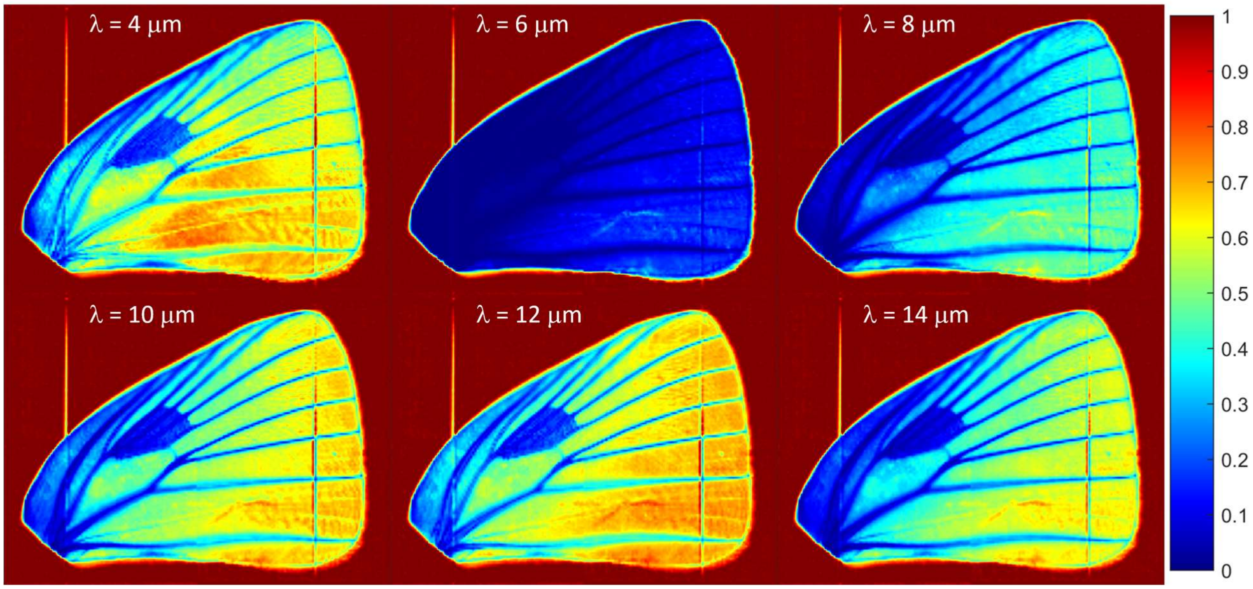

Supplementary Figure 27 | Transmissivity distributions on the forewing of a male *Panthiades aeolus*.

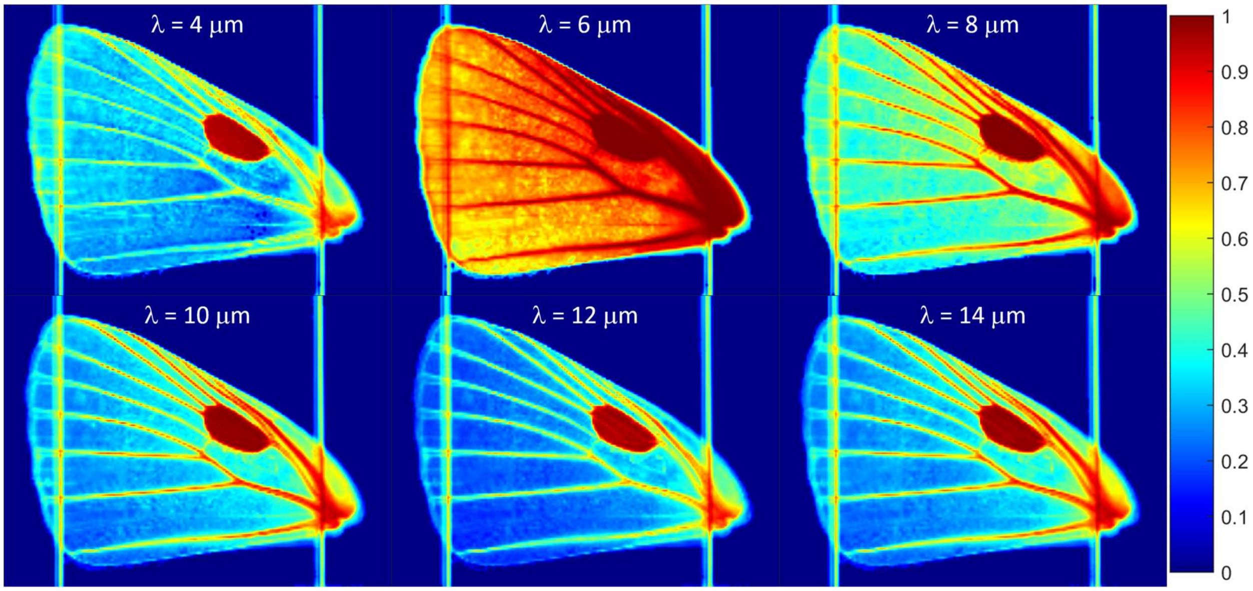

Supplementary Figure 28 | Emissivity distributions on the forewing of a male hickory hairstreak, *Satyrium caryaevorus*.

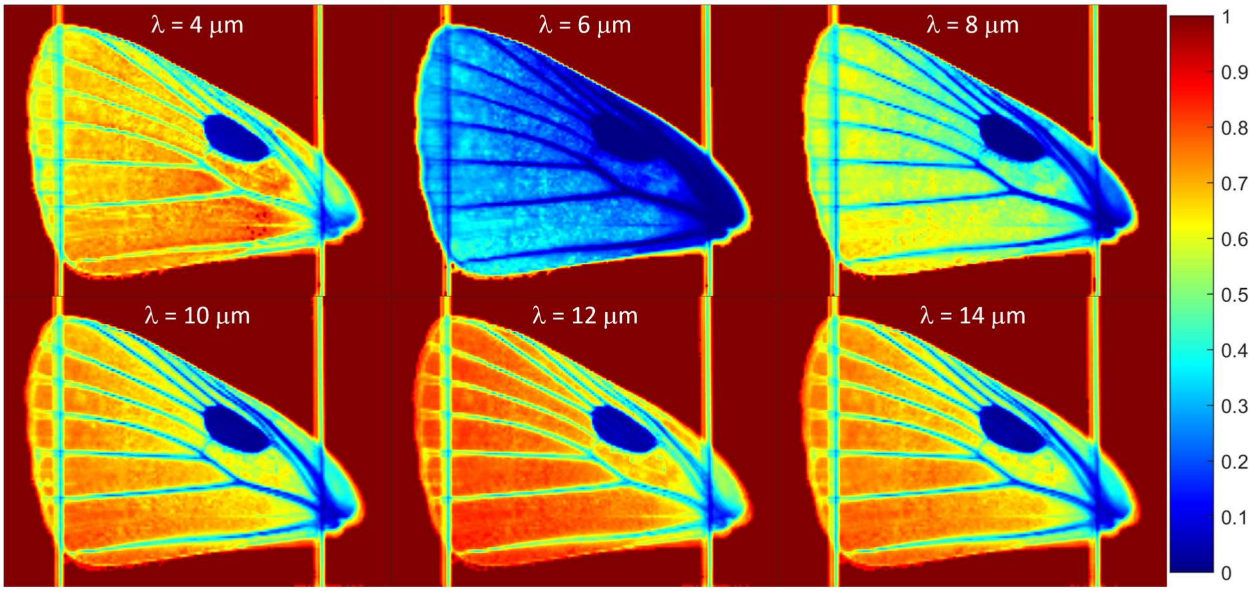

Supplementary Figure 29 | Transmissivity distributions on the forewing of a male hickory hairstreak, *Satyrium caryaevorus*.

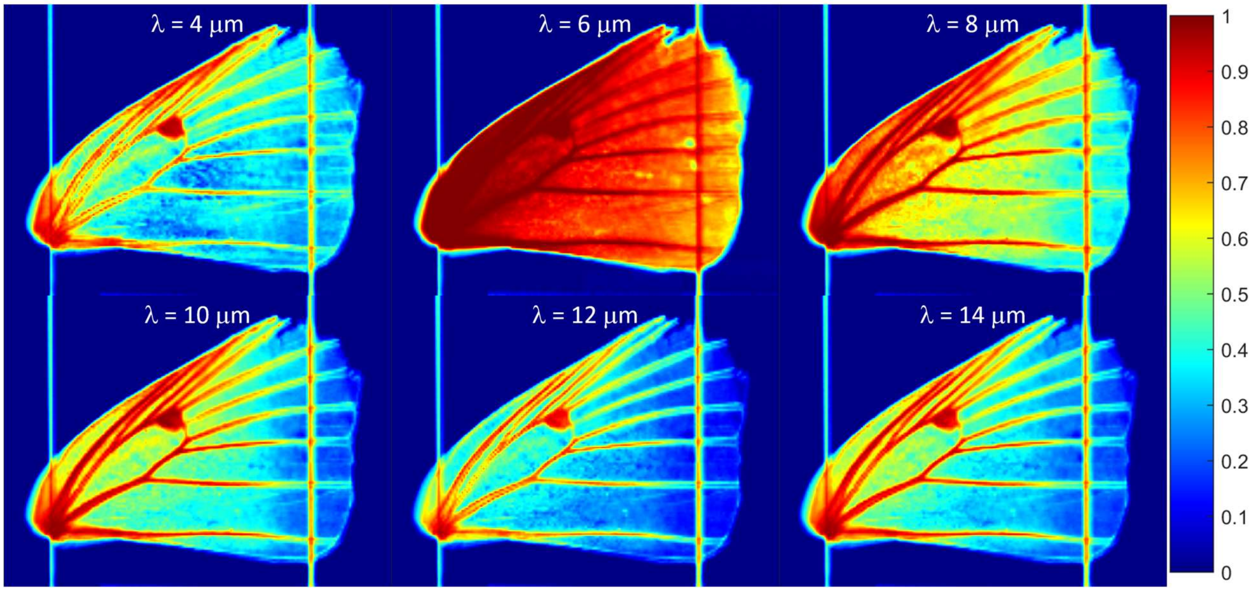

Supplementary Figure 30 | Emissivity distributions on the forewing of a male white-M hairstreak, *Parrhasius m album*.

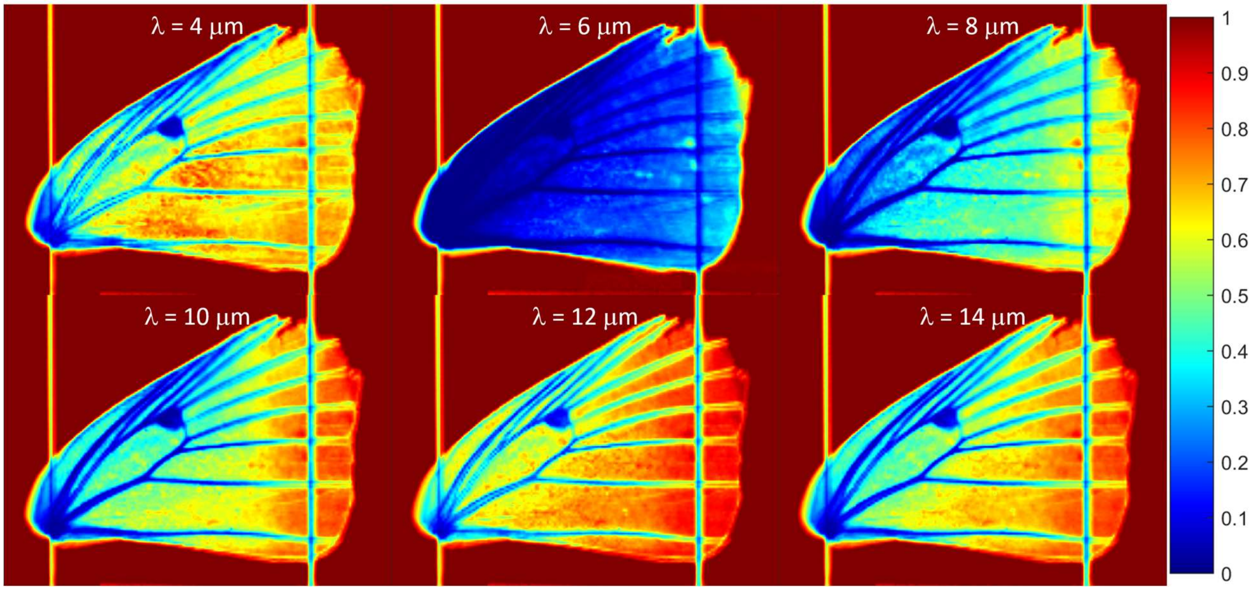

Supplementary Figure 31 | Transmissivity distributions on the forewing of a male white-M hairstreak, *Parrhasius m album*.

Supplementary Note 11: Deriving complex refractive indices of chitin

Complex refractive indices of chitin in the infrared spectral range were obtained using two methods. Solid curves in **Supplementary Fig. 32** were obtained using the Lorentz oscillator model and transfer matrix method, which considers multiple-beam interference effects (i.e., multiple reflection of light inside chitin thin films). Dashed curves in **Supplementary Fig. 32** were obtained based on the Beer-Lambert law and Kramers-Kronig relations: the red dashed curve is the extinction coefficient, *k*, directly calculated from the measured absorption spectrum using the Beer-Lambert law, and the navy blue dashed curve is the corresponding real part of the complex refractive index calculated using the Kramers-Kronig relations. Because the Beer-Lambert law only considers a single pass of light through chitin thin films, the extinction coefficient is overestimated. We used the values based on the Lorentz oscillator model and transfer matrix method (solid curves) for full-wave simulations of the interactions between infrared light and nano-structured wing scales (**Fig. 4h,i** of the main text). The parameters used in the Lorentz oscillator model are summarized in **Supplementary Table 2**.

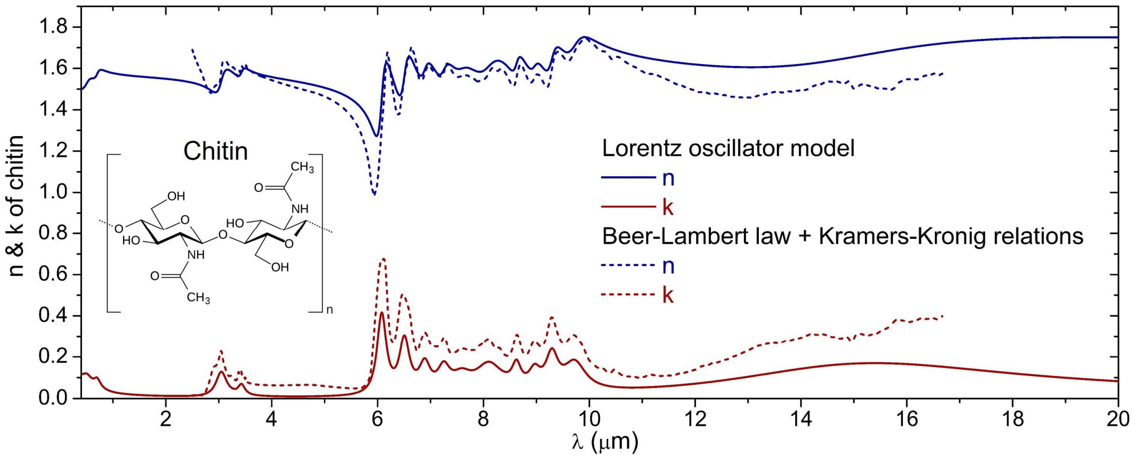

Supplementary Figure 32 | Complex refractive indices of chitin. Inset: Structural formula of the polymer.

Supplementary Table 2 Parameters used in the Lorentz oscillator model of chitin

#	Resonant frequency (cm^{-1})	Resonant wavelength (μm)	$\lambda_p = 2\pi c/\omega_p$ (μm)	γ (10^{15} Hz)	Origin
1	30303	0.33	0.8291	3.4	NA
2	19231	0.52	1.9112	1.8	NA
3	14286	0.7	4.6632	0.6	NA
4	3284	3.0451	21	0.05	O-H, N-H stretching
5	2920	3.4247	48	0.025	C-H stretching
6	1651	6.0569	37.8	0.01	Amide I (C-O stretching)
7	1547	6.4641	46.2	0.01	Amide II (N-H stretching)
8	1456	6.8681	66	0.01	C-H bending
9	1383	7.2307	74.4	0.01	CH_x deformation

10	1306	7.6570	75.6	0.015	Amide III
11	1244	8.0386	52.2	0.018	NA
12	1157	8.6430	115.8	0.005	C_3-OH
13	1115	8.9686	122.4	0.006	C_3-OH
14	1076	9.2937	87	0.006	C_6-OH
15	1034	9.6712	73.2	0.01	C-O-C bridge stretching
16	690	14.4928	36	0.055	NA

Supplementary Note 12: Experimental setup to simulate the natural radiative environment of butterflies

A schematic of the experimental setup is shown in **Supplementary Fig. 33b** and a photo of a wing specimen being tested in the setup is provided in **Supplementary Fig. 33d**. We used a xenon lamp to simulate sunlight shining from above. The butterfly wing specimens were illuminated on either the dorsal or ventral surfaces. The radiative temperature of the laboratory was maintained at 20°C, within the range of the natural terrestrial environmental temperatures. We used a temperature controllable cryostat equipped with an infrared-transparent window beneath the wing specimen to simulate the sky at different weather conditions. A clear sky with low humidity can have T_{sky} of −50°C (**Supplementary Fig. 34**). Conditions of full sun and T_{sky} well above 0°C might be likely in the following two situations: (a) when high humidity blocks the cold outer space (i.e., similar to the greenhouse effect) and (b) when the clear, cold sky is mostly blocked by foliage while a strong beam of sunlight shines between the trees onto butterflies. To create conditions of free convection, no air flow was applied.

Our experimental design (**Supplementary Fig. 33b**) has the same thermodynamic effects on butterfly wings that it would have if the positions of the cryostat and the lab space were inverted (mimicking the natural environment where the sky is above and the terrestrial environment is below, **Supplementary Fig. 33a**). This is because: (a) Our spectroscopy measurements showed that the mid-infrared absorptivity (= emissivity) of butterfly wings measured from the dorsal side is the same as that measured from the ventral side (**Supplementary Fig. 33g**); (b) Butterfly wings are sufficiently thin that when sunlight shines from one side, no temperature gradient can be established across the thickness of the wing (i.e., dorsal and ventral wing surfaces have essentially the same temperature). These two facts indicate that the radiative heat transfer between the wing and the sky is the same whether the sky is positioned above (**Supplementary Fig. 33a**) or below (**Supplementary Fig. 33b**) the wing. A similar argument can be applied to the radiative heat transfer between the wing and the terrestrial environment. Thus, we can conclude that the configurations in **Supplementary Fig. 33a** and **b** are thermodynamically equivalent.

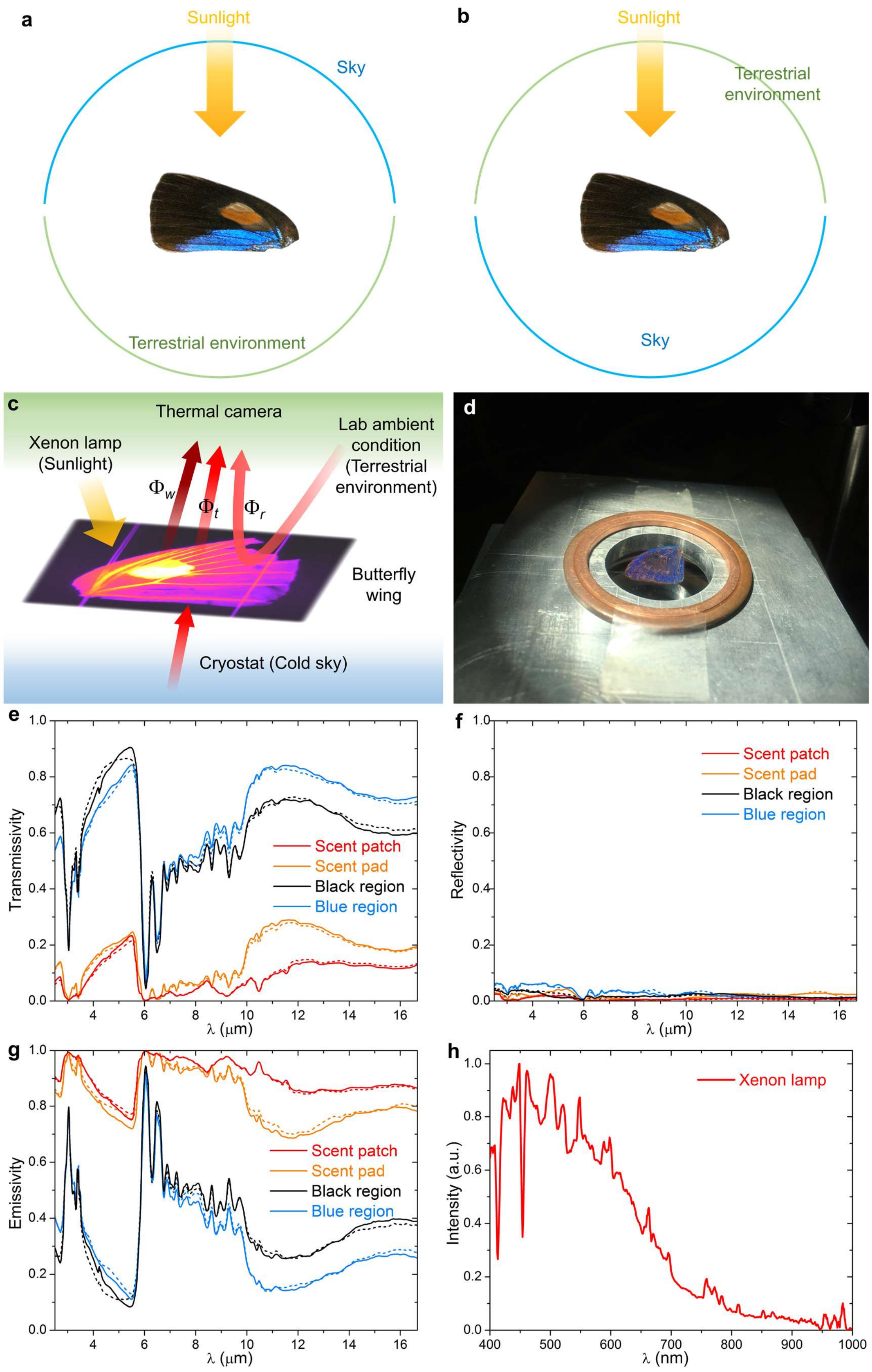
a
Sunlight
Sky
Terrestrial environment
b
Sunlight
Terrestrial environment
Sky
c
Thermal camera
Xenon lamp (Sunlight)
Lab ambient condition (Terrestrial environment)
Φ_w
Φ_t
Φ_r
Butterfly wing
Cryostat (Cold sky)
d
e
Transmissivity
λ (μm)
Scent patch
Scent pad
Black region
Blue region
f
Reflectivity
λ (μm)
Scent patch
Scent pad
Black region
Blue region
g
Emissivity
λ (μm)
Scent patch
Scent pad
Black region
Blue region
h
Intensity (a.u.)
λ (nm)
Xenon lamp

Supplementary Figure 33 | Simulating natural environmental conditions in thermodynamic experiments. (a) A butterfly wing (forewing of *Bistonina biston*, dorsal side up) in the natural environment, where the sky occupies the upper hemisphere and the terrestrial environment occupies the lower hemisphere. (b) The butterfly wing in the lab environment, where for logistic reasons, the positions of the sky and terrestrial environment are inverted (see explanation in text). (c) Schematic showing the experimental setup that simulates natural environmental conditions. Three infrared flux components (i.e., infrared radiation generated by the wing, Φ_w, transmitting through the wing, Φ_t, and reflecting from the wing, Φ_r) detected simultaneously by the thermal camera must be disentangled to determine the correct wing temperature. (d) A male *Theritas hemon* forewing in the experimental setup. It is secured in place by two pairs of thin threads to minimize thermal conduction, and is located right above the circular window of the cryostat, which simulates the sky with various radiative temperatures. (e)-(g) Mid-infrared transmission, reflection, and emissivity spectra, respectively, measured from four different regions on the forewing of *Bistonina biston*. Solid and dashed curves represent, respectively, measurements from the dorsal and ventral sides of the wing. (h) Spectrum of the xenon lamp used in the study.

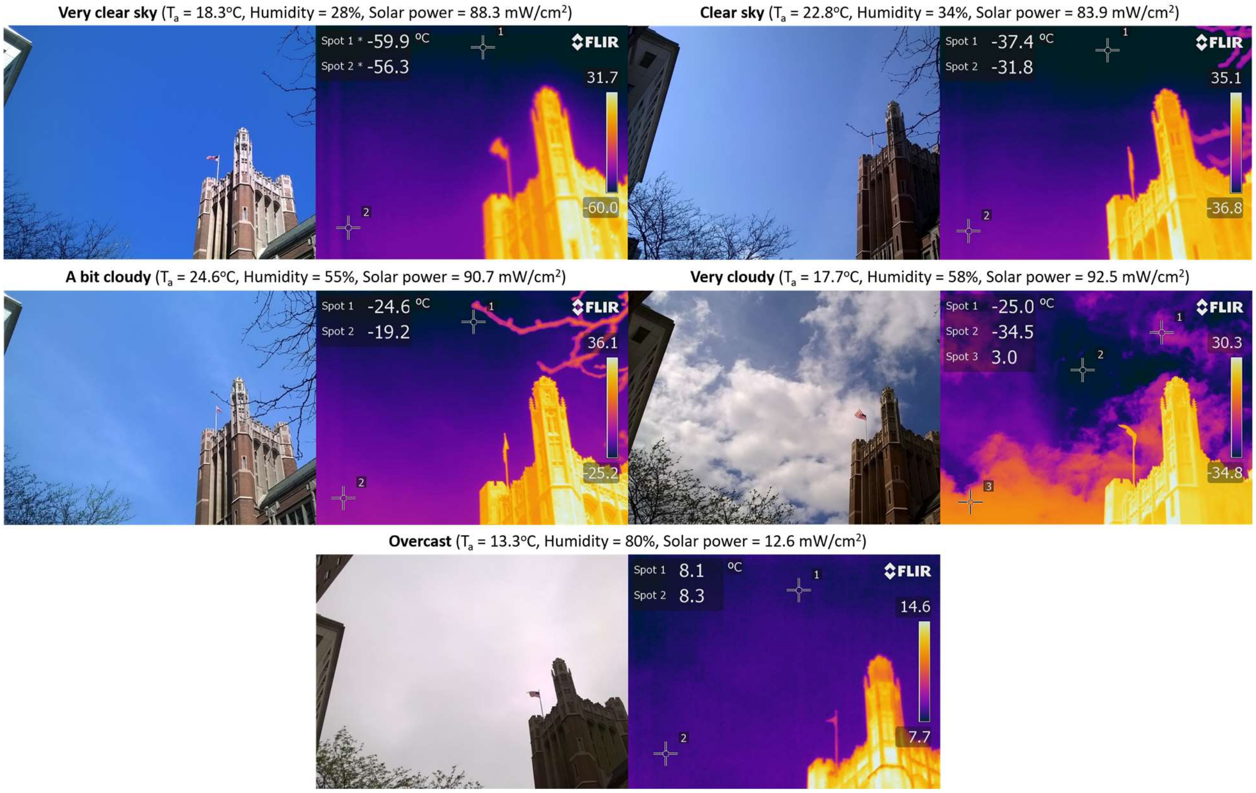

Supplementary Figure 34 | Effects of weather on sky radiative temperatures. Shown are corresponding visible and thermal camera images illustrating the correlation between the clearness of the sky (cloudlessness and low atmospheric humidity) and its ability to act as a radiative heat sink. Thermal radiation through the atmospheric transparency window, λ = 8–13 μm, is how the Earth loses heat to outer space, thereby achieving an energy balance with incoming solar energy and maintaining long-term stability of its temperature. The net radiative energy transfer between a terrestrial object and the sky is the difference between the upward thermal flux produced by the object and the downward thermal flux produced by the sky, Φ_{sky}. Therefore, larger radiative cooling effects are achieved when Φ_{sky}, or the effective radiative temperature of the sky is reduced.

Φ_{sky} has been accurately and systematically measured. For example[5], a continuous measurement over 96 days using a pyrgeometer shows that Φ_{sky} ranged between 165 and 403 W/m^2.

The sky has characteristic spectral and angular distributions of thermal radiation. While it is impossible to create such spectral and angular distributions of the radiative background in the lab, what we can do is to set the cryostat temperatures within a suitable range to simulate natural environmental conditions as well as possible. Our approach is the following: (a) we use two models (the RRTM model and the Swinbank model, to be described below) to calculate the range of downward atmospheric thermal radiance under a variety of atmospheric conditions relevant to the living environment of butterflies; (b) we theoretically calculate the thermal radiance generated by our cryostat setup using the realistic configuration of the setup at different cryostat temperatures; (c) we determine the range of cryostat temperatures so that the associated range of thermal radiance calculated in (b) is approximately the same as that determined in (a). In this way, wing temperatures determined under lab conditions can be estimated as comparable to wing temperatures under the corresponding environmental conditions.

The total thermal radiation flux from the sky in the mid-infrared can be calculated using empirical models. A modified Swinbank model shows that the total radiant flux generated by a clear sky can be estimated using the relation: $\Phi_{sky} = 8.778\times10^{-13}\times T_a^{5.852}\times RH^{0.07195}$, where T_a is the ambient temperature on the surface of the earth and RH is the relative humidity[6]. In addition, algorithms, such as the RRTM model[7] allows one to simulate the atmospheric constituent profiles and calculate integrated atmospheric transmittance taking into account the angular and wavelength dependences of the atmosphere transmittance. We calculated the thermal radiant flux generated by a clear sky (with no cloud cover), using both the empirical Swinbank model and the RRTM model, for various combinations of the ambient temperature and relative humidity (**Supplementary Fig. 35a,b**). The total amount of the downward atmospheric radiance was found to range from 207.5 to 447 W/cm^2 using the Swinbank model, and from 140 to 490 W/cm^2 using the RRTM model, for ambient temperatures varying between 15 and 35°C.

In our experiments, we use a cryostat setup to simulate the cold sky. The thermal radiation of this "artificial sky" is contributed by two components: (a) Within a solid angle around the surface normal direction (half angle θ_c =25°), the wing specimen receives thermal radiation generated by a cooled plate, which is located at the center of the cryostat and has controllable temperatures; (b) Beyond θ_c, the wing specimen receives thermal radiation generated by the non-cooled interior surfaces of the cryostat chamber wall, which is at room temperature, T_a = 20°C. This experimental configuration is qualitatively similar to that of the natural environment, where thermal radiation flux generated by the sky increases from the zenith to the horizon. The total thermal radiation received by the wing specimen from the cryostat setup is thus:

$$\Phi_{bg}=2\pi\int_0^{\theta_c} d\theta sin\theta cos\theta\int_{2.5\mu m}^{40\mu m} d\lambda L\left(T_c,\lambda\right)+2\pi\int_{\theta_c}^{\pi/2} d\theta sin\theta cos\theta\int_{2.5\mu m}^{40\mu m} d\lambda L\left(T_a,\lambda\right), \qquad \text{(Eq. 1)}$$

where

$$L\left(T,\lambda\right)=\frac{2hc^2}{\lambda^5}\frac{1}{exp\left(hc/\lambda k_B T\right)-1}. \qquad \text{(Eq. 2)}$$

The emissivities of the cooled plate of the cryostat and the inner surfaces of the cryostat chamber wall were assumed to be unity, and the spectral range we considered in the above equation is from λ = 2.5 to 40 µm, which covers 95% of the total thermal radiation generated by a black body at 20°C. Calculations using the above equations indicate that the total amount of thermal radiation

generated by the cryostat setup ranges from 289 to 415 W/cm^2 (**Supplementary Fig. 35c**), when the temperature of the cooled plate is changed from T_c = −40 to +20ºC. This range of thermal radiance is similar to that of atmospheric thermal radiance obtained above using the Swinbank model and the RRTM model. Therefore, we believe that setting the cooled plate temperatures between −40 and +20ºC simulates effectively the wide variety of environmental conditions that butterflies experience.

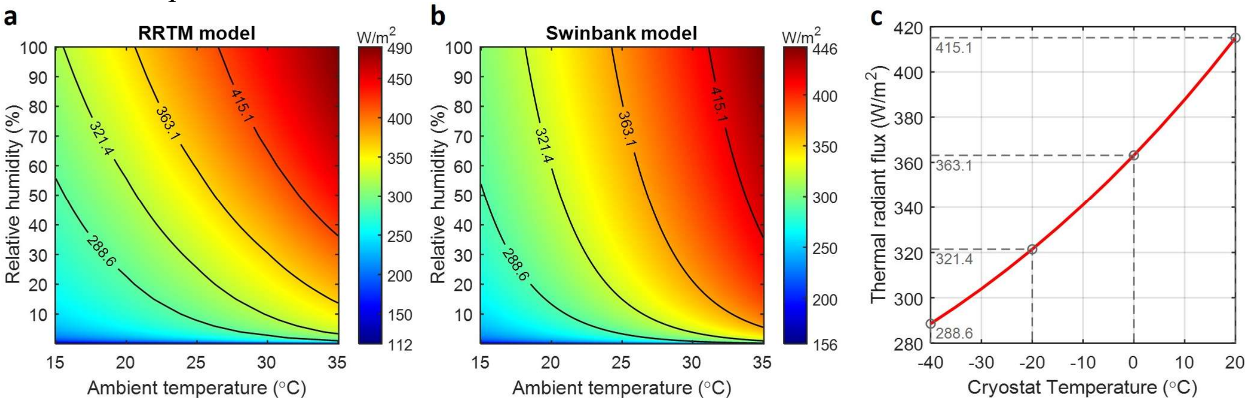

Supplementary Figure 35 | Thermal radiation of sky and cryostat setup. (a) Downwelling atmospheric thermal radiant flux calculated using the RRTM model, as a function of the air temperature and relative humidity on the earth surface. (b) Downwelling atmospheric thermal radiant flux calculated using the empirical, modified Swinbank model, as a function of the air temperature and relative humidity on the earth surface. In (a) and (b), isolines are plotted for the thermal radiant fluxes equivalant to that generated by the cryostat setup at set temperatures of −40, −20, 0, and 20ºC, and corresponding radiant flux of 288.6, 321.4, 363.1, and 415.1 W/m^2, respectively. Figures in (a) and (b) allow one to look for the weather conditions (i.e., T_a and RH) under which the sky generates the same amount of thermal radiation as the cryostat setup at different set temperatures. (c) Calculated thermal radiant flux generated by the cryostat setup as a function of the set temperature of the cooled plate. The total thermal radiant flux varies from 288.6 to 415.1 W/m^2 as the cooled plate temperature changes from −40ºC to 20ºC.

Supplementary Note 13: Determining temperature distributions over butterfly wings

Supplementary Fig. 36 illustrates the three steps employed to derive the temperature distributions over butterfly wings. We started by taking infrared irradiance (Φ_{cam}) pictures of a wing specimen using the thermal camera under experimental conditions that simulate different illumination and environmental conditions (i.e., dorsal or ventral irradiation, high or low background radiative temperature) (**Supplementary Fig. 36a**). These irradiance pictures do not directly represent temperature distributions, because wing specimens are semitransparent in the mid-infrared. We then took the emissivity, $\varepsilon(\lambda)$, transmissivity, $t(\lambda)$, and reflectivity, $r(\lambda)$, maps over the wing specimen (**Supplementary Fig. 36b**). We observed that the androconial organ and the wing veins have enhanced emissivity over the entire mid-infrared spectrum. The enhanced emissivity is either due to special nanostructured wing scales (scent patches) or the physical thickness of unfused wing membranes (scent pads and veins) (**Fig. 4e** of the main text). The measured $\varepsilon(\lambda)$, $t(\lambda)$, and $r(\lambda)$ maps enable us to determine the relative contributions of thermal radiation generated by the wing (Φ_w), transmitting through the wing (Φ_t), and reflecting from the wing (Φ_r), and thus derive the temperature distributions over the wing by solving equation $\Phi_{cam} = \Phi_w + \Phi_t + \Phi_r$ point by point over the entire wing surface.

The angle dependence of the wing emissivity, transmissivity, and reflectivity (ε_w, t_w, and r_w) is weak within the viewing angle of the thermal camera lens (i.e., 25 °) (**Supplementary Fig. 37b**), and thus we used the values of ε_w, t_w, and r_w measured at surface normal direction to derive the wing temperature, T_w (**Fig. 5** of the main text). A comparison between T_w calculated using angle dependent ε_w, t_w, and r_w and those calculated using angle independent ε_w, t_w, and r_w shows that the difference is less than 1.2°C for all wing regions (last column of **Supplementary Table 3**). This comparison indicates that treating the mid-infrared properties of butterfly wings as angle independent is sufficiently accurate to enable us to determine the wing temperature distributions.

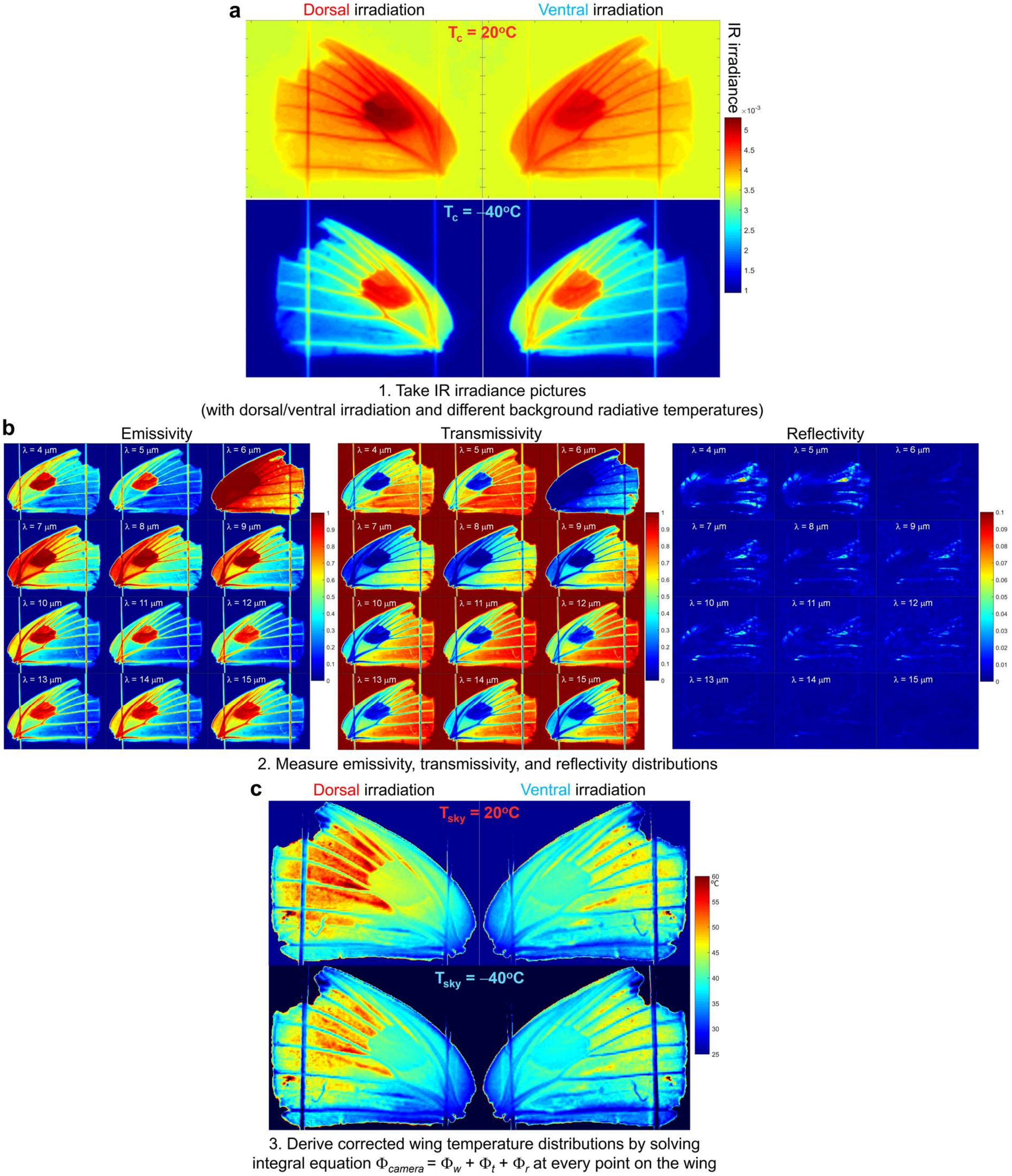

Supplementary Figure 36 | Determining temperature distributions over butterfly wings using infrared hyperspectral imaging. (a) Infrared irradiance pictures of the *Bistonina biston* forewing recorded by the thermal camera under different experimental conditions. The unit for infrared irradiance is W/cm^2/μm/sr (power per unit area, per unit wavelength, and per solid angle). The two straight vertical features in each picture are thin threads that brace the wing during thermodynamic experiments. The tip of the wing was damaged. (b) Left panel: Emissivity distributions on the forewing at 12 different mid-infrared wavelengths. Particularly strong emissivity at λ = 6 μm corresponds to the amide I vibrational band of the protein backbone for insect chitin. Central panel: Transmissivity distributions on the forewing. Regions with low transmissivity correspond to regions of high emissivity. Right panel: Reflectivity distributions on the forewing. The value of the reflectivity is no more than a few percent throughout the wing, and, in general, decreases as wavelength increases. (c) Derived wing temperature distributions under different experimental conditions.

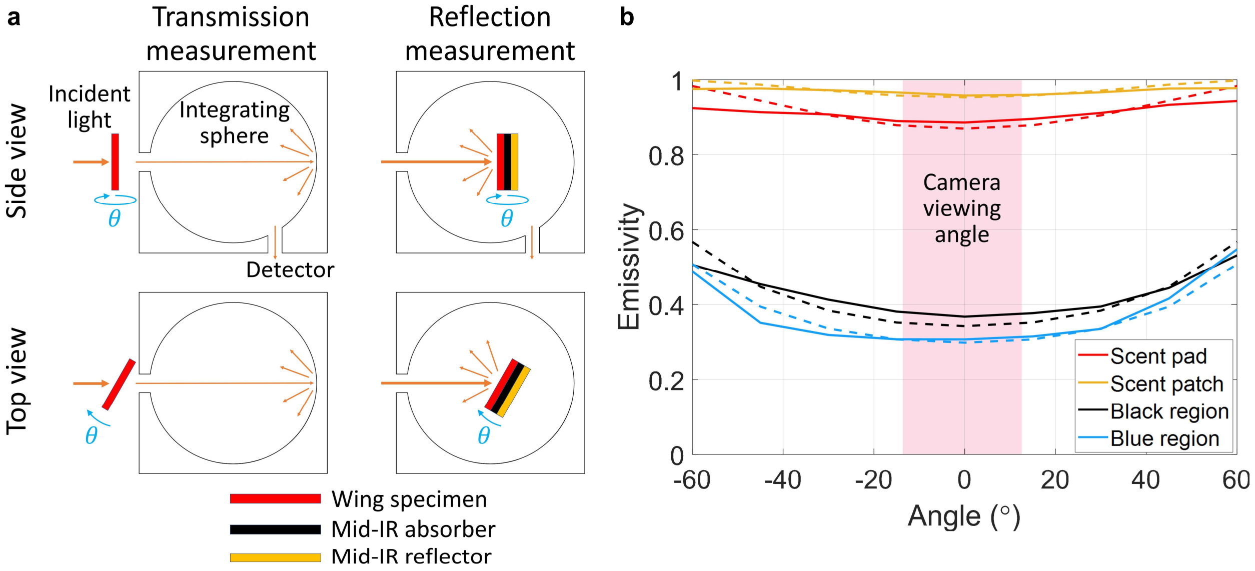

Supplementary Figure 37 | Wing emissivity, transmissivity, and reflectivity are weakly angle dependent. (a) Schematics showing measurements of angle dependence of transmissivity and reflectivity of butterfly wings using an integrating sphere. (b) Solid curves: Measured angle dependent thermal emissivities of the scent pad, scent patch, blue, and black regions on the *Bistonina biston* forewing. The red-shaded area stands for the viewing angle of the thermal camera lens. Dashed curves: Fits to the measured data using the relation between transmissivity and incident angle, which follows Beer's law $log(t_w(\theta)) \propto OPL(\theta) \propto 1/cos\theta$ for a flat slab (*OPL* stands for optical path length).

Supplementary Table 3 Comparison of calculated wing temperatures using angle dependent and angle independent ε_w, t_w, and r_w of four regions on the *Bistonina biston* forewing*

Region	Temperature readings from camera T_{cam} (°C)	T_w calculated using angle dependent ε_w, t_w, r_w (°C)	T_w calculated using angle independent ε_w, t_w, r_w (°C)	Error (°C)
Full sun, T_c = 20°C, dorsal irradiation				
Scent pad	44.31	46.97	47.14	-0.17

Scent patch	46.49	47.55	47.57	-0.02
Black region	33.40	56.01	56.59	-0.59
Blue region	28.26	46.17	46.56	-0.40
Full sun, T_c = –40ºC, dorsal irradiation				
Scent pad	34.76	41.11	41.51	-0.43
Scent patch	41.38	43.93	43.98	-0.05
Black region	4.29	56.55	57.72	-1.17
Blue region	-5.64	45.74	46.70	-0.96

*The angle dependent ε_w, t_w, and r_w were measured using an integrating sphere, and the angle independent ε_w, t_w, and r_w were measured with the incident light normal to the wing. The camera readings, T_{cam}, were taken with the wing under irradiation on the dorsal side by a xenon lamp simulating full sun. This table shows the calculated wing temperatures, T_w, under two extreme conditions when the cryostat temperatures, T_c, were set to be 20ºC and –40ºC. Area of measurement is ~50 pixels in the thermal camera.

Supplementary Note 14: Thermodynamic studies of butterfly wings

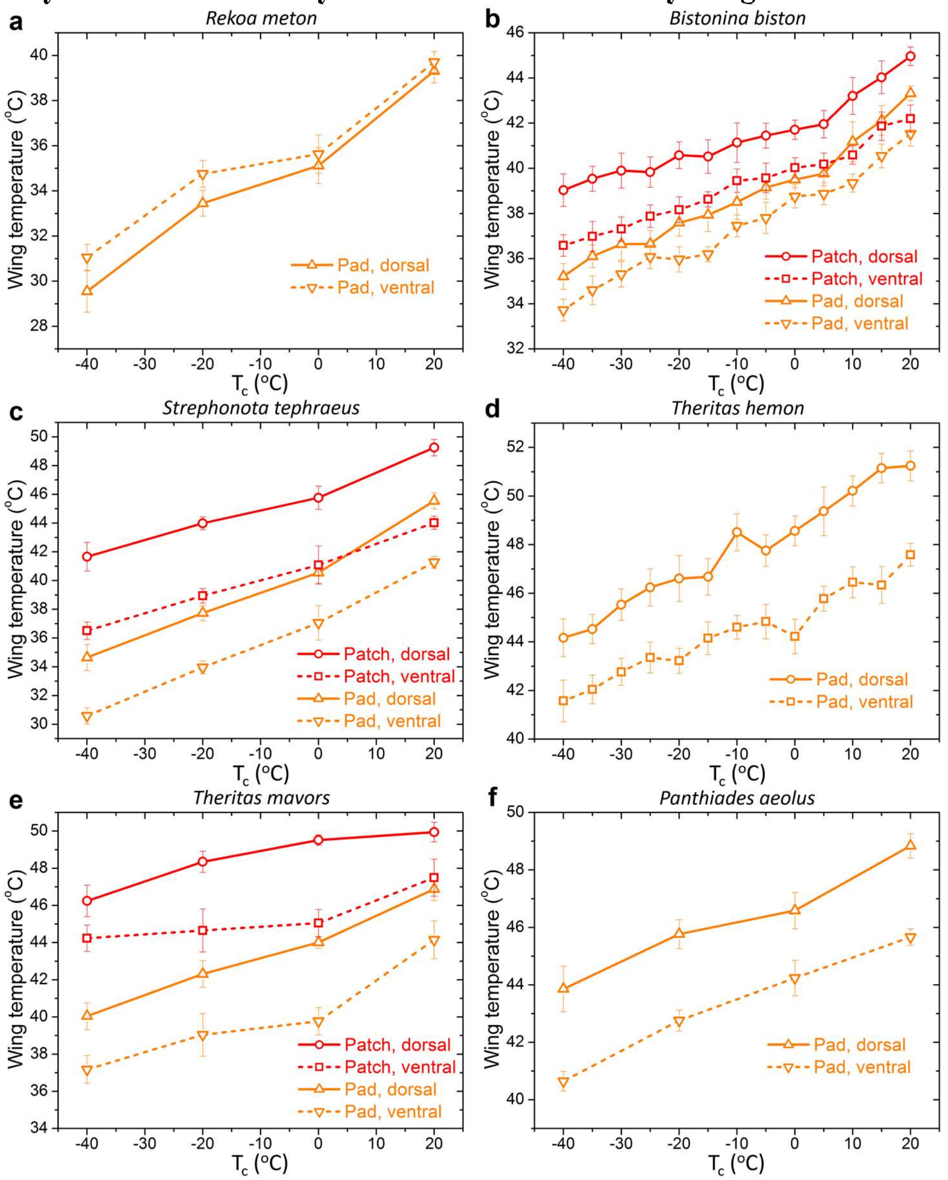

Supplementary Figure 38 | Temperatures of scent patches and pads of six Eumaeini butterflies as a function of the cryostat temperature T_c. The experimental results show that a reduction of T_c from +20ºC to −40ºC leads to a substantial decrease in temperature of 5-10ºC for the scent patches and pads. This illustrates that the sky serving as a cold, radiative heat sink plays an important role in reducing wing temperatures: under full sun, the scent patches and pads are much cooler if the butterfly is fully exposed to the sky and if the humidity of the atmosphere is low. The results also show that ventral irradiation with the xenon lamp (mimicking the sun) leads to cooler wing temperatures than dorsal irradiation due to differences in the wing solar reflectivities of these two surfaces (i.e., dashed curves below corresponding solid curves), the exception being *Rekoa meton,* whose scent pads are bright white viewed from the dorsal side, but brown viewed from the ventral side.

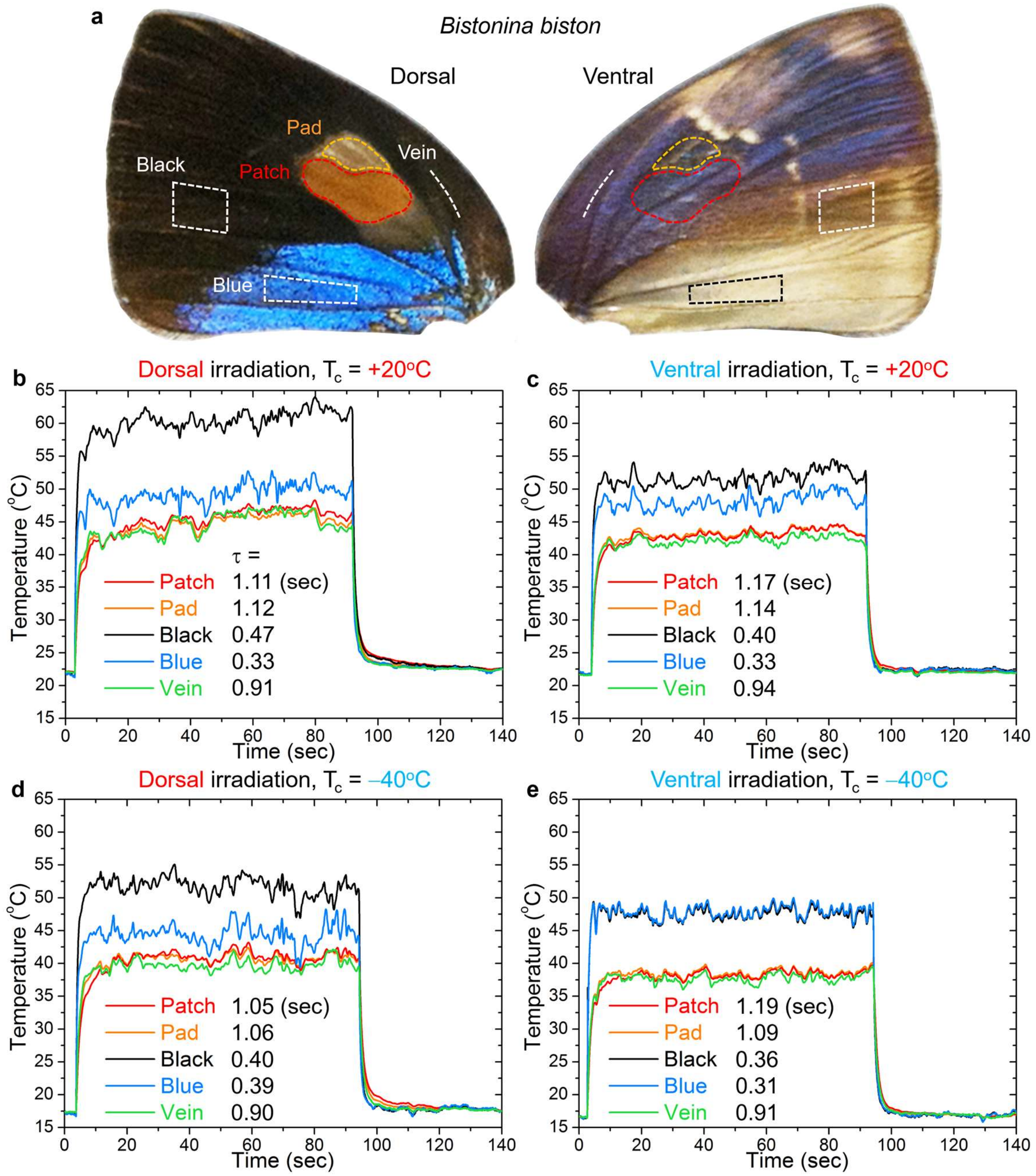

Supplementary Figure 39 | Results of thermodynamic experiments on *Bistonina biston* forewing. (a) Photos of the dorsal and ventral *B. biston* forewing, showing the five regions under study. (b)-(e) Temperature traces for the five regions under different experimental conditions. The results show that different regions of the wing have different time constants of temperature changes (Scent patch: 1.13 ± 0.06 seconds, Scent pad: 1.10 ± 0.03 seconds, black region: 0.41 ± 0.05 seconds, blue region: 0.34 ± 0.03 seconds, and wing vein: 0.91 ± 0.02 seconds). Overall, it does not take more than a few seconds for the entire wing to reach the thermodynamic steady state. The black and blue regions have particularly quick temperature changes (time constants <0.5 seconds) as they are physically thin, consisting of fused membranes and typically only two to three layers of wing scales on each surface. The time constants of the bulkier wing veins, scent patches, and pads are three to four times larger than those of the black and blue regions. The experiments were

conducted with dried wing specimens. In live butterflies, the scent pads are filled with pheromones and/or hemolymph, and wing veins are partially filled with hemolymph; therefore, the mass and time constants of temperature changes of these wing parts could be considerably larger than their dried counterparts. For example, the time constants extracted from basking experiments with live hickory hairstreaks, *Satyrium caryaevorus*, which have a comparable wing span to *B. biston*, are 3.52 ± 0.47 seconds and 2.40 ± 0.72 seconds, respectively, for the scent pad and wing veins.

Supplementary Note 15: Theoretical model for calculating steady-state butterfly wing temperatures

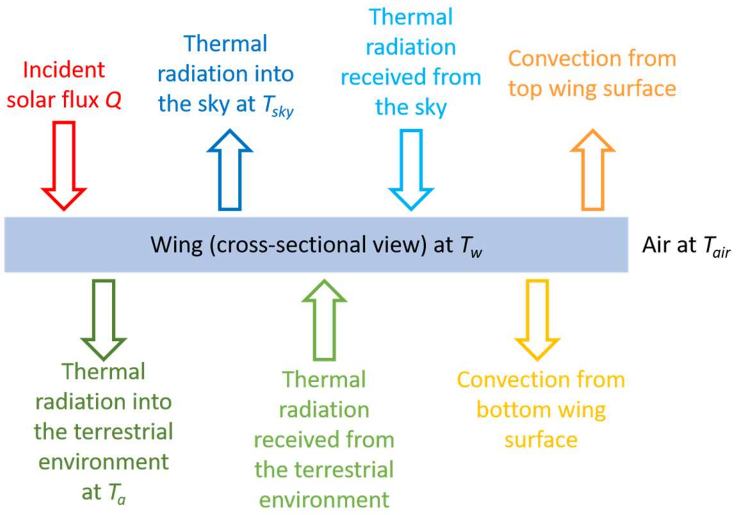

Supplementary Figure 40 | Theoretical model used to calculate steady-state temperatures of a horizontal butterfly wing suspended in still air.

The conservation of energy of a butterfly wing in thermodynamic steady state while suspended in air and in the horizontal direction can be written as:

$$\begin{aligned} &QS\left(1-T_{sun}-R_{sun}\right)+\sigma A_{T_{sky}}^{wing}T_{sky}^{4}S+\sigma A_{T_a}^{wing}T_{a}^{4}S \\ &=2\sigma\varepsilon_{T_w}^{wing}T_{w}^{4}S+\left(h_{top}+h_{bn}\right)\left(T_{w}-T_{air}\right)S \end{aligned} \quad \text{(Eq. 3)}$$

The physical meaning of each term in the above equation is explained below:

(1) $QS\left(1-T_{sun}-R_{sun}\right)$ is the absorbed solar power. T_{sun} and R_{sun} are, respectively, the transmissivity and reflectivity of the wing integrated over the solar spectrum; $1 - T_{sun} - R_{sun}$ is solar absorptivity; Q is the light intensity (power per unit area) incident onto the wing; S is the area of the wing.

(2) $\sigma A_{T_{sky}}^{wing}T_{sky}^{4}S$ is the absorbed thermal radiative power from the sky. σ is the Stefan-Boltzmann constant 5.67×10^{-8} $Wm^{-2}K^{-4}$; $A_{T_{sky}}^{wing}$ is the absorptivity of the wing integrated over the spectrum of a blackbody at the radiative temperature of the sky, T_{sky}.

(3) $\sigma A_{T_a}^{wing}T_{a}^{4}S$ is the absorbed thermal radiative power from the ambient environment. $A_{T_a}^{wing}$ is the absorptivity of the wing integrated over the spectrum of a blackbody at the radiative temperature of the terrestrial environment, T_a.

(4) $2\sigma\varepsilon_{T_w}^{wing}T_w^4 S$ is the thermal radiation from the wing, including both of its surfaces, to the surrounding environment. $\varepsilon_{T_w}^{wing}$ is the emissivity of the wing and T_w is the wing temperature.

(5) $\left(h_{top}+h_{bn}\right)\left(T_w-T_{air}\right)S$ is the power transferred through thermal convection from the wing to the surrounding air. h_{top} and h_{bn} are, respectively, heat transfer coefficients of the top and bottom surfaces of the wing; T_{air} is the temperature of air surrounding the wing.

Note that we have treated the wing as a homogeneous object with uniformly distributed optical properties (absorptivities and emissivity). The wing is sufficiently thin so that the top and bottom surfaces have the same temperature, T_w.

The heat transfer coefficient from the top surface of the wing, h_{top}, can be calculated in the following procedure:

(1) Calculate the Rayleigh number of characteristic length *L*:

$$Ra_L = \frac{g\beta\left(T_w - T_a\right)L^3}{\nu^2} Pr \qquad \text{(Eq. 4)}$$

Here g = 9.81 m/s^2 is the acceleration of gravity; $\beta = 2/(T_{air} + T_w)$ 1/K is the thermal expansion coefficient; *L* is the characteristic length of the wing and can be calculated by *A*/*P*, where *A* and *P* are, respectively, the area and perimeter of the wing; ν is the kinematic viscosity of air and is 1.807×10^{-5} m^2/s at 325 K; and *Pr* is the Prandtl number of air, which is weakly dependent on T_{air} and is 0.701 at 325 K.

(2) Calculate the average Nusselt number of natural or free convection from the top surface of a heated horizontal plate, using correlations developed in supplementary references 8 and 9:

$$\overline{Nu}_L = \begin{cases} 0.96Ra_L^{1/6} & \text{for } 1<Ra_L<200 \\ 0.59Ra_L^{1/4} & \text{for } 200<Ra_L<10^4 \\ 0.54Ra_L^{1/4} & \text{for } 2.2\times10^4<Ra_L<8\times10^6 \\ 0.15Ra_L^{1/3} & \text{for } 8\times10^6<Ra_L<1.5\times10^9 \end{cases} \qquad \text{(Eq. 5)}$$

(3) Calculate the thermal convection coefficient for the top surface of the wing:

$$h_{top} = \frac{\overline{Nu}_L k}{L} \qquad \text{(Eq. 6)}$$

where *k* is the thermal conductivity of air and is 2.816×10^{-2} W/m/K at 325 K.

The heat transfer coefficient for the bottom surface of the wing, h_{bn}, can be calculated by following the same procedure, except that the correlation used for calculating the average Nusselt number should be the following for natural convection from the bottom surface of a heated horizontal plate:

$$\overline{Nu}_L = 0.27Ra_L^{1/4} \qquad \text{(Eq. 7)}$$

In the case of small lycaenid butterflies, the Rayleigh number (which is proportional to L^3, where *L* characterizes the dimension of the butterfly wings) is between 1 and 200, so we used the first formula in **Eq. 5**. Numerical calculations show that h_{top} is typically a few times larger than h_{bn}, which makes physical sense as natural convection from the top surface of a heated horizontal thin plate (i.e., a butterfly wing) is stronger than that from the bottom surface of the plate: heated air rises up from the top surface without interruption, while heated air underneath the plate has to flow to the edge of the plate to rise up.

It is important to point out the limitations of the above thermodynamic model: (a) It treats the entire wing as a homogeneous object (e.g., the *Bistonina biston* forewing consists entirely of scent patch, scent pad, blue, *or* black region) and cannot model wings with heterogeneous distributions of solar absorptivity and thermal emissivity; (b) It considers the effect of thermal convection averaged over the entire wing surface (i.e., average Nusselt numbers used in **Eqs. 5** and **7**) and cannot model the complex effects as a result of a spatial variation of thermal convection (e.g., peripheral regions close to the wing margins have stronger thermal convection compared to the central region). Nevertheless, the thermodynamic model still has the predictive power of determining the relative temperatures of different wing regions based on their solar absorptivity and thermal emissivity. The model is reasonably accurate in determining the temperatures of scent patches and pads, probably because they are large features on the butterfly wings (compared to, for example, thin wing veins) and are typically located far from the wing margins where thermal convection could be considerably stronger.

Supplementary Equation 3 enables us to solve for steady-state temperatures of butterfly wings under different environmental conditions, including the intensity of sunlight, radiative temperatures of the sky and of the terrestrial environment, and air temperature. Calculated steady-state temperatures as a function of solar absorptivity and thermal emissivity under four conditions are shown in **Supplementary Fig. 41**, from which we observe that:

(1) Higher thermal emissivity and lower solar absorptivity lead to lower steady-state wing temperatures.

(2) The solar absorptivity of the black region is only moderately larger (by ~0.1) than that of the scent patch/pad for dorsal sunlight irradiation, and comparable to that of the scent patch/pad for ventral sunlight irradiation, but the thermal emissivity of the scent patch/pad is substantially larger (by ~0.6) than that of the black region. As a result, the scent patch and pad are always cooler than the black region (**Supplementary Fig. 41b–e**), in agreement with experimentally determined wing temperature distributions (**Fig. 5b** of the main text).

(3) The solar absorptivity of the blue region is comparable to that of the scent patch/pad for dorsal sunlight irradiation, and moderately smaller (by ~0.15) than that of the scent patch/pad for ventral sunlight irradiation. The thermal emissivity of the scent patch/pad is however substantially larger (by ~0.6) than that of the blue region. As a result, the calculated temperature of the blue region is slighter higher than or comparable to that of the scent patch and pad. We observed that under all experimental conditions (**Fig. 5b** of the main text) the temperature of the blue region is comparable to that of the scent patch and pad. The blue region is not warmer, probably because it is close to the inner wing margin and thus experiences larger convective cooling than do the scent patch and pad.

(4) The contribution of solar absorptivity to heating up butterfly wings increases when solar intensity increases (e.g., from half sun to full sun: **Supplementary Fig. 41b→d** and **c→e**); the contribution of thermal emissivity to cooling down butterfly wings increases when the radiative temperature of the sky reduces (e.g., from 20°C to −40°C: **Supplementary Fig. 41c→b** and **e→d**). The relative contribution of thermal emissivity in cooling down butterfly wings and solar absorptivity in heating them up can be represented as the slope of the equal temperature curves (e.g., dashed curves) in the 2D temperature maps. It is seen that under half sun and a sky temperature of −40°C (**Supplementary Fig. 41b**), solar absorptivity and thermal emissivity have equivalent thermodynamic effects on butterfly wings: per-unit increase of solar absorptivity in increasing wing temperatures is canceled by per-unit increase of thermal emissivity in decreasing wing temperatures. However, under full sun and a much higher sky

temperature of 20°C (**Supplementary Fig. 41e**), the effect of solar absorptivity is enhanced and that of thermal emissivity is reduced so that the dashed curve is much steeper: per-unit increase of solar absorptivity can only be canceled by a ~4.5-unit increase of thermal emissivity.

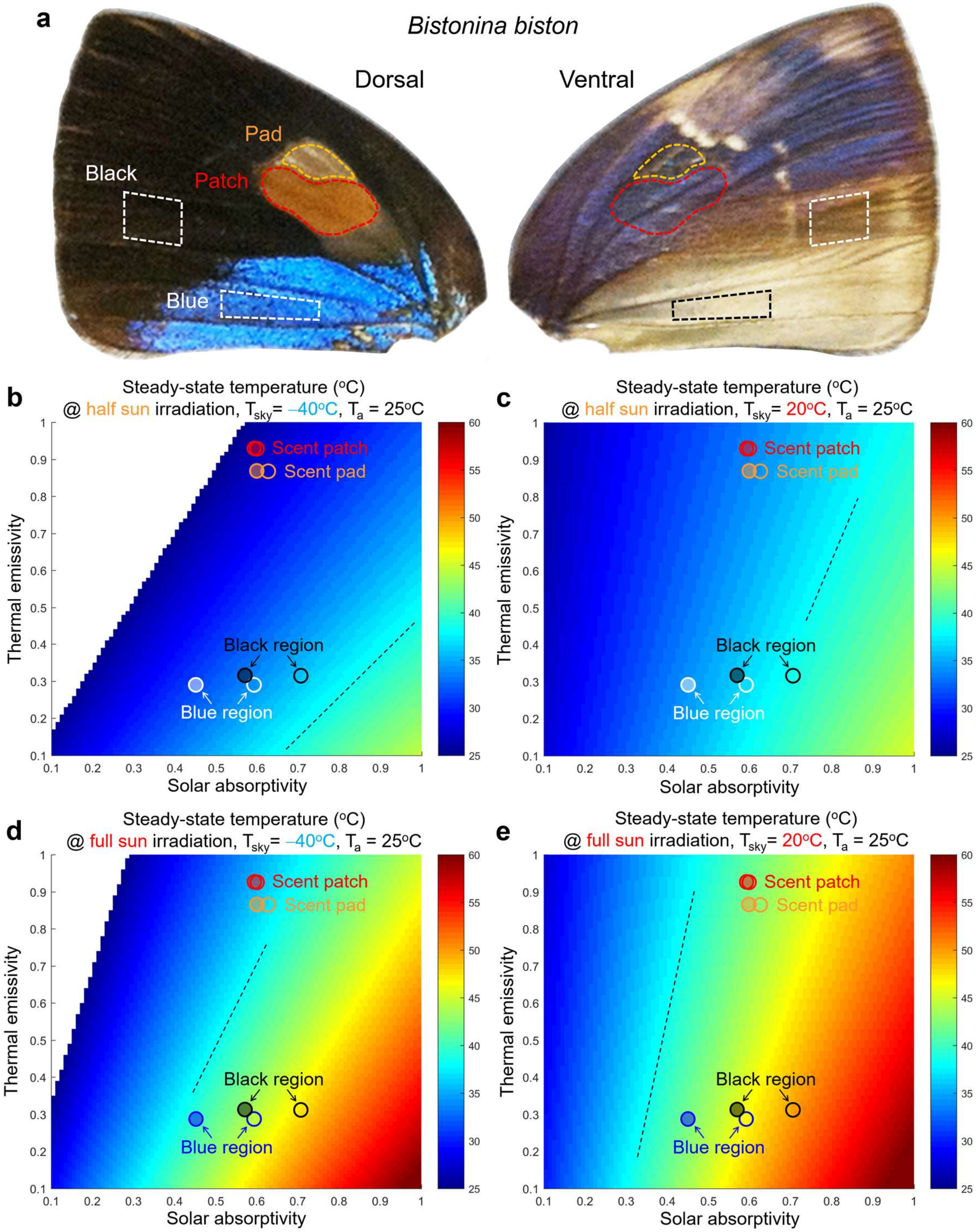

Supplementary Figure 41 | Results of theoretical calculations of steady-state butterfly wing temperatures. (a) Photos of the dorsal and ventral *Bistonina biston* forewing. The four regions under study (i.e., scent patch, scent pad, specific areas of black and blue) are marked. (b)-(e)

Calculated steady-state wing temperatures (by solving **Eq. 3**) as a function of solar absorptivity (horizontal axis) and thermal emissivity (vertical axis) under different sunlight intensities and radiative temperatures of the sky. Points representing the four regions are marked in the temperature maps according to measured solar absorptivity and thermal emissivity of the four regions. Hollow circles are for the cases of dorsal solar irradiation, and filled circles are for the cases of ventral solar irradiation. Corresponding hollow and filled circles are offset in the horizontal direction because solar absorptivities measured from the dorsal and ventral wing surfaces at the same location are different, but thermal emissivities measured from the two surfaces are the same (**Supplementary Fig. 33g**, **Supplementary Table 4**). Dashed lines indicate constant temperatures as a result of the counterbalance between solar absorptivity and thermal emissivity, and the slope of the dashed lines indicate the relative efficiency of solar absorptivity in heating up the wing versus thermal emissivity in cooling down the wing. The thermodynamic model is developed for the situations in which wings are warmer than the air (**Eqs. 5** and **7** are only valid for hot plates in cool air); therefore, the situations in which the wings are colder than the air are left as white in (b) and (d).

Supplementary Table 4 Solar absorptivity & thermal emissivity of the *Bistonina biston* forewing*

	Solar absorptivity (dorsal irradiation)	Solar absorptivity (ventral irradiation)	Thermal emissivity
Scent pad	0.63	0.60	0.87
Scent patch	0.60	0.59	0.93
Black	0.71	0.57	0.32
Blue	0.59	0.45	0.29

*Absorptivity values are calculated by integrating measured absorption spectra and the solar spectrum; emissivity values are calculated by integrating measured thermal emissivity spectra and the thermal radiation spectrum of a blackbody at 30°C.

Supplementary Equation 3 also enables us to calculate the steady-state wing temperatures as a function of the radiative temperature of the sky, T_{sky}, while other environmental conditions remain unchanged. The results for the four regions of *B. biston* forewing are shown in **Supplementary Fig. 42**. As T_{sky} drops from +20°C to −40°C, the wing temperatures decrease because of enhanced heat dissipation via thermal radiation from the wing to the sky. Such radiative cooling effects are large for the scent patch and pad compared with the black and blue regions, because the scent patch and pad have much higher thermal emissivity and thus the flux of thermal radiation (which is proportional to thermal emissivity) from them to the cold sky is greater. **Supplementary Fig. 42a** shows that with dorsal irradiation when T_{sky} decreased from +20°C to −40°C, the temperature of the scent patch and pad of *B. bison* decreases by 6.2°C and 5.8°C, respectively, which agrees reasonably well with the experimental results (**Fig. 5b** of the main text) showing that the temperature of the scent patch and pad decreased by 5.8°C and 7.9°C, respectively. The decrease of scent pad temperature in experiments is larger than the theoretical value probably because the scent pad borders the thick and cool leading edge (costa) of the forewing (**Supplementary Fig. 41a**) and, as a result, there is substantial conductive heat transfer from the scent pad to the costa, which is not considered in the theoretical model.

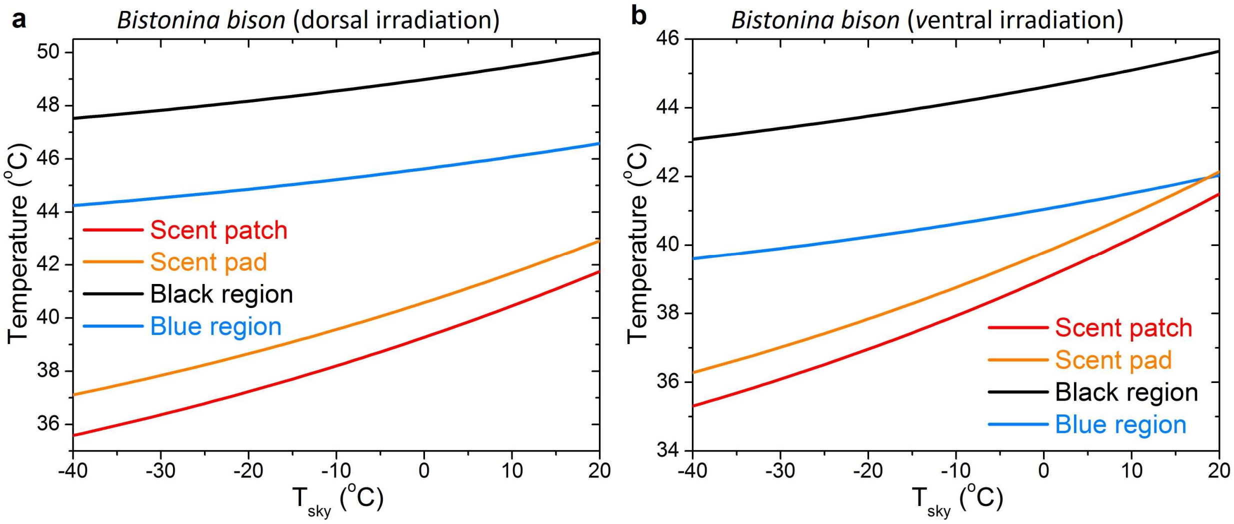

Supplementary Figure 42 | Effects of T_{sky} on butterfly wing temperatures. (a) Calculated steady-state wing temperatures (by solving **Eq. 3**) as a function of the radiative temperature of the sky, T_{sky}, under dorsal irradiation of full sun. (b) Calculated steady-state wing temperatures as a function of T_{sky} under ventral irradiation of full sun.

Supplementary Note 16: Laser-induced local heating experiments and controls

We conducted additional experiments on adult *Vanessa cardui* to determine whether the butterflies react to overheating on the wing veins and to rule out (to the best of our ability) other factors of stimulation. The study was carried out under three different conditions:

(1) The laser spot was positioned at the first junction of the radial veins on the forewing (red circle in **Supplementary Fig. 43a**);

(2) The laser spot ~1 mm in diameter was positioned at the center of the forewing discal cell, away from surrounding wing veins (blue circle in **Supplementary Fig. 43a**);

(3) The laser beam was directed at the substrate ~1 cm in front of the butterfly's head (**Supplementary Fig. 43b**).

In conditions #1 and #2, the laser power was adjusted to heat up the illuminated wing regions so that the equilibrium temperature would reach >50°C provided that the butterfly did not move. In condition #3, movements in response to the laser being turned on or off were recorded; the butterflies were either dark-adapted for 3 minutes before turning on the laser, or light-adapted for the same duration of time before turning off the laser; a laser intensity of 50 mW/mm^2 was used.

In order to assess the possible visual stimulation created by the laser spot (conditions #1 and #2), we used the same laser spot treatment but applied it to the substrate ~1 cm in front of the stationary butterflies, thereby creating a visual-only stimulus. The relative number of times that the butterflies responded to the laser beam under the three conditions described above was recorded (**Supplementary Fig. 43c**). When the beam was pointed at the wing vein junction (i.e., condition #1), the reaction of butterflies (n = 27) was triggered by the temperature change of the illuminated area and the proportion of response was 100%; the average threshold temperature was 42.395°C. When the beam was pointed at the area between the veins (i.e., condition #2), the proportion of response was 0% (n = 10), suggesting an absence of thermal sensors in the membranous region of the discal cell between the wing veins. This agrees with studies of the morphology of butterfly wings carried out by us and others[10-12] indicating that fused membranes between wing veins do not

show features of living cells. The results of these experiments support the hypothesis that the behavior of displacing the stimuli applied to the wings is a response to the increase in temperature on their wing veins generated by the laser. The relative number of times that a butterfly responded to the laser beam when it was positioned on the substrate in front of the butterfly was also recorded (i.e., condition #3) (**Supplementary Fig. 43c**). The reaction was triggered by visual stimuli due to the abrupt luminance change when the laser was switched on or off. The large variance in likelihood of response (n = 10) show that at least some individuals were responding to the visual treatment. Importantly, since the distance of the laser spot to the eyes in all three conditions was comparable, the results support the hypothesis that the butterflies tested in conditions #1 and #2 may not be able to see the laser spot on their wings and were responding primarily to the thermal rather than the visual stimulus on the wing vein.

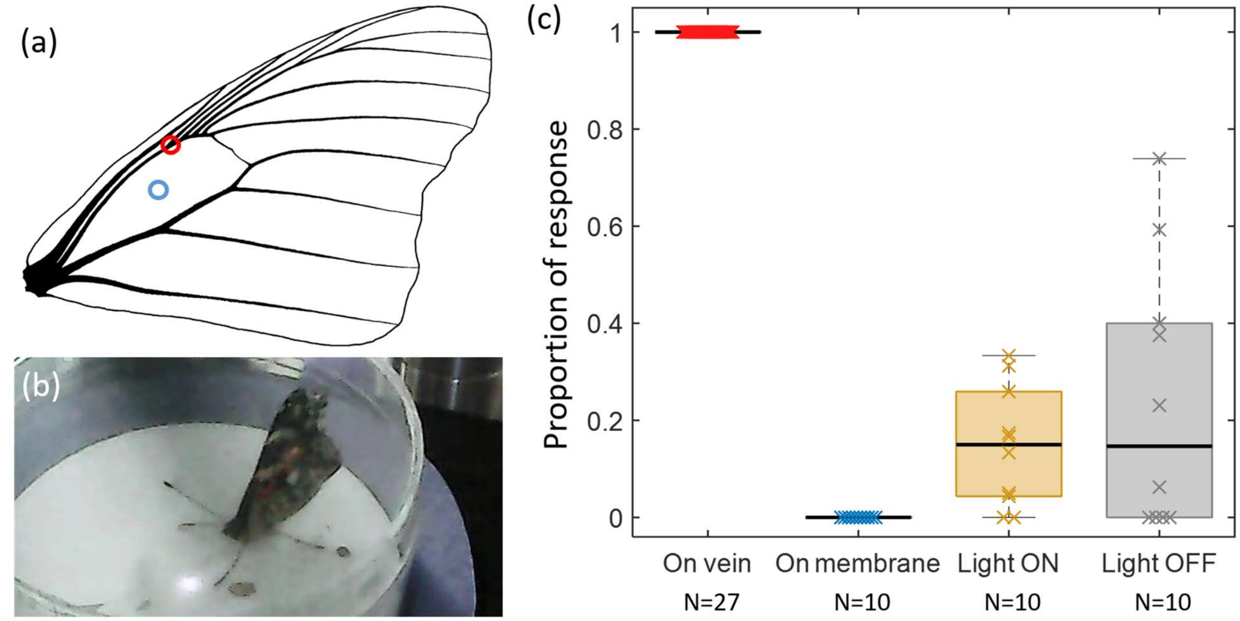

Supplementary Figure 43 | Laser-induced local heating experiments and controls comparing the proportion of responses of adult *Vanessa cardui* to a laser beam positioned on different locations. (a) Schematic showing the venation of the *V. cardui* forewing. The red circle indicates the location and size of the laser spot in the experiments, and the blue circle the location and size of the laser spot in one of the controls (i.e., "On membrane"). (b) Photo showing settings of another control experiment where a laser spot positioned in front of the butterfly's head was turned on or off. (c) Data showing the proportion of responses: (1) when the laser beam was pointed to the first junction of the forewing radial veins, corresponding to the red circle in (a) ("On vein"); (2) when the laser beam was pointed to the center of the discal cell between the veins, corresponding to the blue circle in (a) ("On membrane"); (3) when the laser beam positioned on the substrate in front of the butterfly's head and initially blocked was switched on ("Off→On"); (4) when the laser beam positioned on the substrate in front of the butterfly's head and initially switched on was abruptly blocked ("On→Off").

Supplementary Note 17: Displacement response induced by local heating on different wing regions

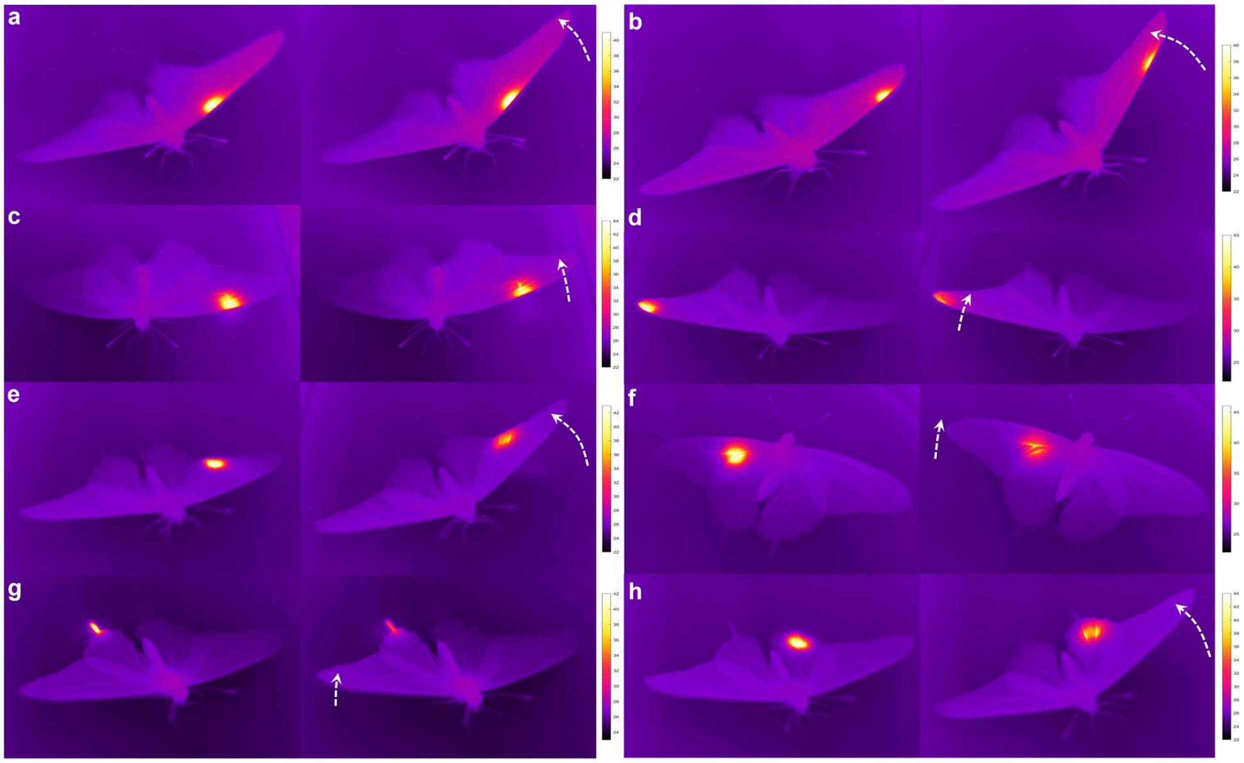

Supplementary Figure 44 | Displacement response induced by local heating on different wing regions of the black swallowtail, *Papilio polyxenes*. (a)-(h) Pairs of photos taken by the thermal camera showing that responses can be elicited by shining a laser spot on various locations on the wing of *P. polyxenes*, as long as the laser spot is positioned on a wing vein to induce local temperatures to reach 40-45°C. The dashed arrow in the second photo of each subfigure indicates the direction and magnitude of wing movement. Notice that in (g) the response can be induced when the laser spot is positioned on the “tail” of the hindwing, which contains a wing vein.

Supplementary Note 18: Heat avoidance and basking behaviors of *Satyrium caryaevorus* in the field

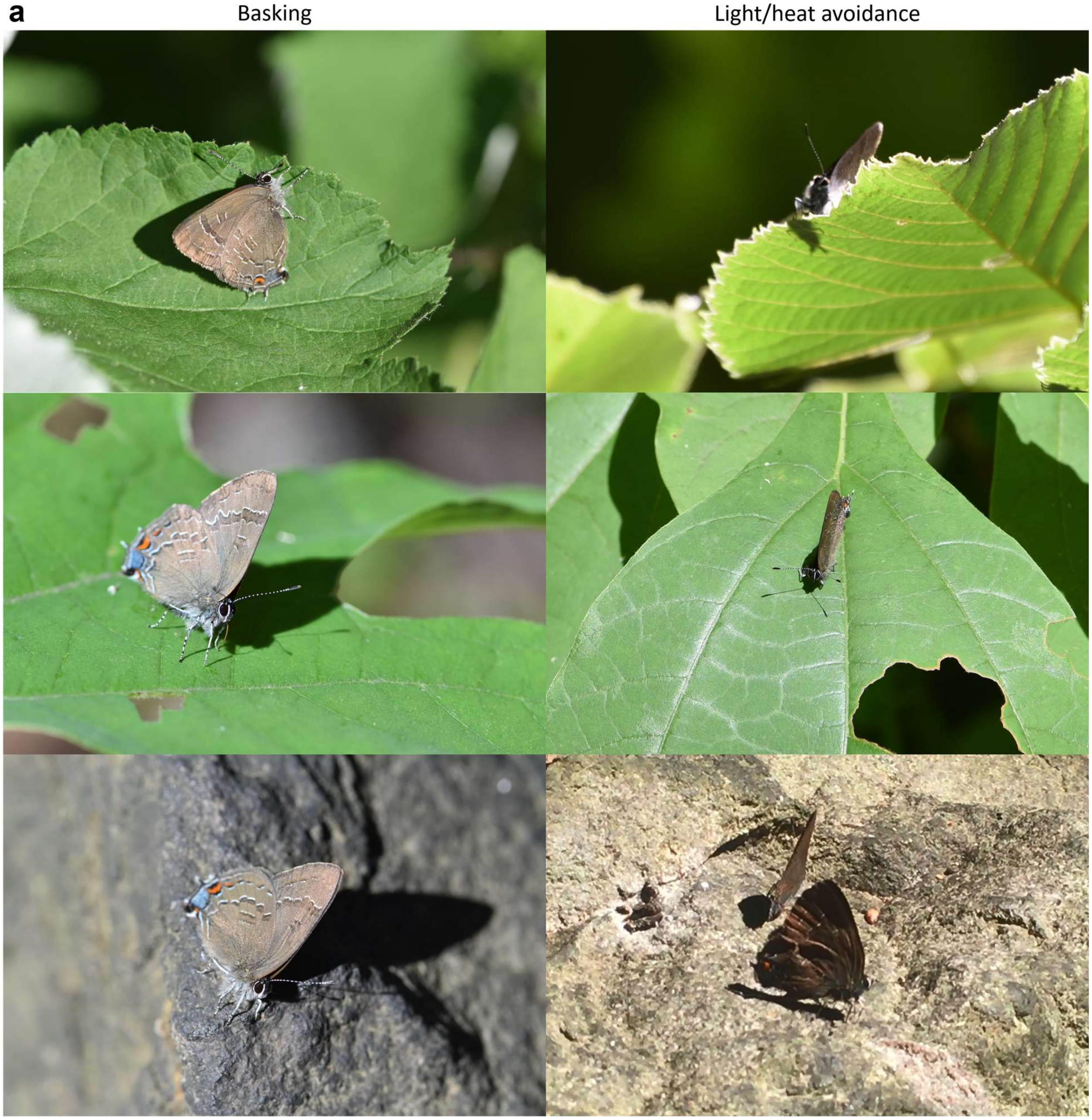

b

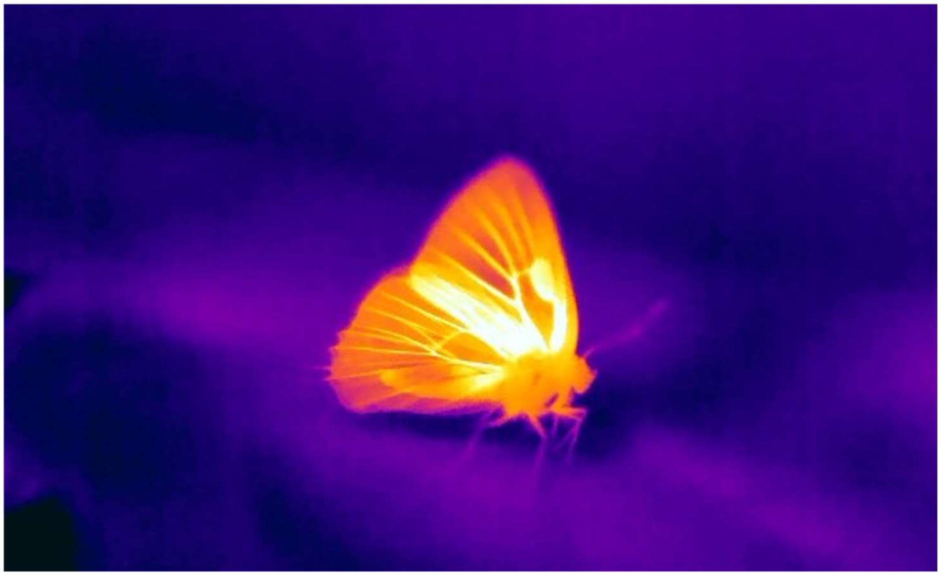

Supplementary Figure 45 | Thermoregulatory behaviors of *S. caryaevorus* in the field. (a) Left: Photos of basking *S. caryaevorus*. Individuals almost always bask in the sun within a few seconds after landing (**Supplementary Movie 12**). Right: Photos of *S. caryaevorus* adopting a heat avoidance posture with shadow areas minimized. Heat avoidance behavior is often observed in the middle of the day. (b) Thermal camera image showing a male *S. caryaevorus* basking in the sun. Because its wings have small thermal capacity compared with the thorax, the instantaneous temperatures of the wing veins and the scent pad are much higher than that of the thorax.

Supplementary Note 19: Butterfly specimens used in this study

Both museum specimens and live butterflies were analyzed in this study, and vouchers have been deposited in the DNA and Tissues Collection of the Museum of Comparative Zoology (MCZ), Harvard University (**Supplementary Tables 5–7**).

Supplementary Table 5 Butterflies used in this study

Type of experiments		Species	Number of individuals	Figures & Tables
**Observation of living butterfly wings**	Morphological study of internal structures in wings	*Vanessa cardui*	22	Fig. 1, Suppl. Fig. 4
		Parrhasius m-album	1	Fig. 2, Suppl. Fig. 5
		Satyrium caryaevorus	7	Fig. 2
	Microscopy of living cells in wings stained with methylene blue	*Vanessa cardui*	12	Fig. 1, Suppl. Fig. 1
		Satyrium caryaevorus	5	Suppl. Fig. 2
	Fluorescence microscopy of living cells in wings stained with fluorescent dye calcein-AM	*Vanessa cardui*	2	Suppl. Fig. 3
	Study of hemolymph flow in wings	*Vanessa cardui*	22	Fig. 1, Suppl. Figs. 4, 6
		Parrhasius m-album	1	Fig. 2
		Satyrium caryaevorus	15	Fig. 2, Suppl. Fig. 5
**Thermodynamics of butterfly wings**	Morphological study of wing scale nanostructures	*Bistonina biston, Rekoa meton, Strephonota tephraeus, Theritas hemon, Theritas mavors, Panthiades aeolus, Satyrium caryaevorus, Parrhasius m-album*	One individual per species	Fig. 4, Suppl. Fig. 9
	Spectroscopic study of dried wing specimens in the solar and thermal radiation spectra	8 species, one individual per species<br>See **Supplementary Table 6**		Fig. 4, Suppl. Figs. 18–31, 36, 37
	Temperature of butterfly wings	*Bistonina biston, Rekoa meton, Strephonota tephraeus, Theritas*	One individual per species	Fig. 5, Suppl. Figs. 36, 38, 39

		hemon, Theritas mavors, Panthiades aeolus		
**Displacement response induced by local heating on butterfly wings**	Displacement response induced by local heating on wings of field-collected butterflies	861 individuals from 50 species<br>See **Supplementary Table 7**		Fig. 6, Suppl. Fig. 44
	Displacement response induced by local heating on butterfly wings over lifespan	*Vanessa cardui*	27	Suppl. Fig. 6
	Impact of overheating on sensory function of butterfly wings	*Vanessa cardui*	18	Suppl. Fig. 7
	Laser-induced local heating and controls (shining laser light on non-living membranous region of wings, on substrate)	*Vanessa cardui*	27	Suppl. Fig. 43
**Basking behavior of butterflies**		*Parrhasius m-album*	10	Fig. 7
		Satyrium caryaevorus	6	Fig. 7, Suppl. Fig. 45
		Satyrium favonius	4	Fig. 7

Supplementary Table 6 Dried wing specimens used in spectroscopic studies

**Genius, Species**	**Specimen ID**	**Collection location**	**G. I. S.**
Bistonina biston	TQ-05-F324	Road Iquitos-Nauta, Loreto, Peru	-4.130, -73.467
Rekoa meton	MCZ-ENT 00069664 (A. G. Weeks Collection)	Suapure, Venezuela	7.233, -65.167
Strephonota tephraeus	MCZ-ENT 00069791	Brazil	NA
Theritas hemon	MCZ-ENT 00069265 (A. G. Weeks Collection)	NA	NA
Theritas mavors	MCZ-ENT 00069207 (A. G. Weeks Collection)	Suapure, Venezuela	7.233, -65.167
Panthiades aeolus	MCZ-ENT 00070155 (A. G. Weeks Collection)	Suapure, Venezuela	7.233, -65.167
Parrhasius m-album	NY-17-PM001	Englewood Cliffs, NJ	40.892, -73.943
Satyrium caryaevorus	NY-17-SC001	Englewood Cliffs, NJ	40.892, -73.943

Supplementary Table 7 Field-collected live butterflies used in laser-induced local heating experiments

	**Genus, Species**	**Specimen ID**	**Collection location**	**G. I. S.**	**Date**
**Hesperiidae**	*Atalopedes campestris*	JT-17-AC001–015	Fort Lee, NJ	40.848, -73.974	9/11/2017
		NY-17-AC001–008	Englewood Cliffs, NJ	40.892, -73.943	7/26-10/6/2017
		XY-17-AC001–014	Fort Lee, NJ	40.848, -73.974	9/26-10/2/2017
	Epargyreus clarus	NY-17-EC001–026	Englewood Cliffs, NJ	40.892, -73.943	7/10-9/11/2017
	Erynnis juvenalis	NY-17-EJ001	Englewood Cliffs, NJ	40.892, -73.943	9/26/2017
	Euphyes vestris	NY-17-EV001–020	Englewood Cliffs, NJ	40.892, -73.943	7/11-9/11/2017
	Hylephila phyleus	NY-17-HP001–007	Englewood Cliffs, NJ	40.892, -73.943	9/26-10/19/2017
	Lerodea eufala	DM-17-LE001–004	Tucson, AZ	32.204, -110.955	10/19-11/17/2017
	Panoquina ocola	NY-17-PO001–002	Englewood Cliffs, NJ	40.892, -73.943	10/2-10/6/2017
	Pholisora catullus	NY-17-PC001–003	Englewood Cliffs, NJ	40.892, -73.943	7/25-8/20/2017
	Poanes viator	NY-17-PV001–003	Englewood Cliffs, NJ	40.892, -73.943	8/17-8/20/2017
	Poanes zabulon	NY-17-PZ001–050	Englewood Cliffs, NJ	40.892, -73.943	8/8-8/20/2017
	Polites peckius	NY-17-PP005–012	Englewood Cliffs, NJ	40.892, -73.943	8/14-9/28/2017
		NY-18-PP001–002	Englewood Cliffs, NJ	40.892, -73.943	5/1/2018
	Wallengrenia egeremet	NY-17-WE001–002	Englewood Cliffs, NJ	40.892, -73.943	7/10/2017
**Lycaenidae**	*Arhopala japonica*	MH-17-AJ001–004	Hyogo, Japan	34.768, 135.348	10/23/2017
	Calycopis cecrops	NY-17-CC001–082	Englewood Cliffs, NJ	40.892, -73.943	8/7-10/6/2017
	Celastrina ladon	NY-17-CL001–005	Englewood Cliffs, NJ	40.892, -73.943	7/5-8/20/2017
	Celastrina neglecta	NY-17-CN001–012	Englewood Cliffs, NJ	40.892, -73.943	7/11-9/11/2017
	Cupido comyntas	NY-17-CC083–090	Englewood Cliffs, NJ	40.892, -73.943	7/11-8/22/2017
	Eumaeus atala	NY-17-EA002–017	Miami, FL	25.662, -80.281	3/17/2017
		NY-18-EA001–019	Miami, FL	25.662, -80.281	5/1/2018
	Parrhasius m-album	NY-17-PM001–014	Englewood Cliffs, NJ	40.892, -73.943	8/9-9/25/2016
	Satyrium caryaevorus	NY-17-SC001–063<br>NY-19-SC001	Englewood Cliffs, NJ	40.892, -73.943	7/5-7/11/2017<br>6/20/2019
	Satyrium favonius	JCD-17-SF001–004	Gainesville, FL	29.634, -82.368	4/20/2017
	Strymon melinus	NY-17-SM001–014	Englewood Cliffs, NJ	40.892, -73.943	7/11-10/6/2017

**Nymphalidae**	*Agraulis vanillae*	DM-17-AV001–013	Tucson, AZ	32.204, -110.936	9/7-11/27/2017
	Asterocampa celtis	NY-17-AC009	Englewood Cliffs, NJ	40.892, -73.943	8/13/2017
	Coenonympha tullia	NY-17-CT001	Englewood Cliffs, NJ	40.892, -73.943	8/30/2017
	Danaus gilippus	DM-17-DG001–005	Tucson, AZ	32.204, -110.949	11/16-11/17/2017
	Danaus plexippus	NY-17-DP001–095	Englewood Cliffs, NJ	40.892, -73.943	7/26-10/16/2017
	Euptoieta claudia	NY-17-EC001	Englewood Cliffs, NJ	40.892, -73.943	6/18/2017
	Junonia coenia	NY-17-JC001–013	Englewood Cliffs, NJ	40.892, -73.943	7/7-9/5/2017
	Limenitis arthemis	NY-17-LA001–006	Englewood Cliffs, NJ	40.892, -73.943	8/13-8/24/2017
	Nymphalis antiopa	NY-18-NA001–002	Englewood Cliffs, NJ	40.892, -73.943	4/1/2018
	Phyciodes tharos	NY-17-PT020–025	Englewood Cliffs, NJ	40.892, -73.943	7/7-7/8/2017
	Polygonia comma	NY-18-PC001	Englewood Cliffs, NJ	40.892, -73.943	6/11/2018
	Polygonia interrogationis	NY-18-PI001<br>NY-19-PI001	Englewood Cliffs, NJ	40.892, -73.943	5/20/2018<br>6/17/2019
	Speyeria cybele	NY-17-SC010–012	Englewood Cliffs, NJ	40.892, -73.943	7/12-8/21/2017
	Vanessa atalanta	CCT-17-VA001–007	Central Park, New York, NY	40.806, -73.959	7/12-7/18/2017
		NY-17-VA001–004	Englewood Cliffs, NJ	40.892, -73.943	7/25-10/17/2017
	Vanessa cardui	NY-17-VC001–050	Englewood Cliffs, NJ	40.892, -73.943	7/25-10/17/2017
	Vanessa virginiensis	NY-17-VV001–008<br>NY-19-VV001	Englewood Cliffs, NJ	40.892, -73.943	7/12-10/30/2017<br>4/21/2019
**Papilionidae**	*Battus philenor*	NY-17-BP001–025	Fort Lee, NJ	40.848, -73.974	4/8-9/2/2017
		NY-18-BP001–105	Fort Lee, NJ	40.848, -73.974	3/1-5/30/2018
	Papilio glaucus	NY-17-PG001–015	Englewood Cliffs, NJ	40.892, -73.943	7/13-9/5/2017
	Papilio polyxenes	NY-17-PP001–004	Englewood Cliffs, NJ	40.892, -73.943	7/16-9/9/2017
	Papilio rumiko	DM-17-PR001–003	Tucson, AZ	32.204, -110.959	10/19-11/16/2017
	Papilio troilus	NY-17-PT001–019	Englewood Cliffs, NJ	40.892, -73.943	7/16-9/5/2017
**Pieridae**	*Colias eurytheme*	CCT-17-CE001–003	Central Park, New York, NY	40.782, -73.970	7/6/2017
		NY-17-CE001–024	Englewood Cliffs, NJ	40.892, -73.943	7/12-10/30/2017
	Colias philodice	CCT-17-CP001	Central Park, New York, NY	40.782, -73.970	7/6/2017
		NY-17-CP001–002	Englewood Cliffs, NJ	40.892, -73.943	9/5-10/30/2017
	Eurema mexicana	DM-17-EM001	Tucson, AZ	32.204, -110.954	9/15/2017
	Phoebis sennae	DM-17-PS001–003	Tucson, AZ	32.204, -110.962	9/15/2017
	Pieris rapae	CCT-17-PR001–007	Central Park, New York, NY	40.806, -73.959	7/6-7/7/2017
		NY-17-PR001–005	Englewood Cliffs, NJ	40.892, -73.943	7/13-9/11/2017
		NY-18-PR001–003	Englewood Cliffs, NJ	40.892, -73.943	4/1/2018
**Riodinidae**	*Apodemia mormo*	DM-18-AM001–011	Oracle, AZ	32.607, -110.734	5/8/2018

Supplementary References

1. Sanes, J. R. & Hildebrand, J. G. Nerves in the antennae of pupal *Manduca sexta* Johanssen (Lepidoptera: Sphingidae). *Wilhelm Roux' Archiv.* **178**, 71–78 (1975).

2. Benjamini, Y. & Hochberg, Y. Controlling the False discovery rate: A practical and powerful approach to multiple testing. *Journal of the Royal Statistical Society. Series B* **57**, 289–300 (1995).

3. Cerdá, X., Retana, J. & Cros, S. Critical thermal limits in Mediterranean ant species: Trade-off between mortality risk and foraging performance. *Functional Ecology* **12**, 45–55 (1998).

4. Leuenberger, P., Ganscha, S., Kahraman, A., Cappelletti, V., Boersema, P. J., von Mering, C., Claassen, M. & Picotti, P. Cell-wide analysis of protein thermal unfolding reveals determinants of thermostability. *Science* **355**, eaai7825 (2017)]

5. Goforth, M. A., Gilchrist, G. W. & Sirianni, J. D. Cloud effects on thermal downwelling sky radiance. *Proc. SPIE* **4710**, 203–213 (2002)]

6. Swinbank, W. C. Long-wave radiation from clear skies. *Q. J. Royal Meteorol. Soc.* **89**, 339–348 (1963)]

7. http://climatemodels.uchicago.edu/rrtm/index.html, Atmospheric and Environmental Research.

8. McAdams, W. *Heat Transmission* (Mc-Graw-Hill, New York, 1954).

9. Lloyd, J. R. & Moran, W. R. Natural convection adjacent to horizontal surface of various planforms. *J. Heat Transfer* **96**, 443–447 (1974).

10. Ai, H., Yoshida, A. & Yokohari, F. Vibration receptive sensilla on the wing margins of the silkworm moth, Bombyx mori. *J. Insect Physiol.* **56**, 236–246 (2010).

11. Yoshida, A. & Emoto, J. Variations in the arrangement of sensory bristles along butterfly wing margins. *Zoolog. Sci.* **28**, 430–437 (2011).

12. Dickerson, B. H., Aldworth, Z. N. & Daniel, T. L. Control of moth flight posture is mediated by wing mechanosensory feedback. *J. Exp. Biol.* **217**, 2301–2308 (2014).
