# Supplementary material for: Physical and behavioral adaptations to prevent overheating of the living wings of butterflies
Source: Nat Commun. 2020 Jan 28;11:551. doi: 10.1038/s41467-020-14408-8 (PMC6987309; doi:10.1038/s41467-020-14408-8)
Supplement: Supplementary file 2 — Reporting Summary [file 41467_2020_14408_MOESM2_ESM.pdf]

nature research

Corresponding author(s): Nanfang Yu, Naomi Pierce

Last updated by author(s): Nov 7, 2019

Reporting Summary

Nature Research wishes to improve the reproducibility of the work that we publish. This form provides structure for consistency and transparency in reporting. For further information on Nature Research policies, see Authors & Referees and the Editorial Policy Checklist.

Statistics

For all statistical analyses, confirm that the following items are present in the figure legend, table legend, main text, or Methods section.

n/a	Confirmed	
☐	☒	The exact sample size (*n*) for each experimental group/condition, given as a discrete number and unit of measurement
☐	☒	A statement on whether measurements were taken from distinct samples or whether the same sample was measured repeatedly
☒	☐	The statistical test(s) used AND whether they are one- or two-sided<br>*Only common tests should be described solely by name; describe more complex techniques in the Methods section.*
☒	☐	A description of all covariates tested
☒	☐	A description of any assumptions or corrections, such as tests of normality and adjustment for multiple comparisons
☐	☒	A full description of the statistical parameters including central tendency (e.g. means) or other basic estimates (e.g. regression coefficient) AND variation (e.g. standard deviation) or associated estimates of uncertainty (e.g. confidence intervals)
☒	☐	For null hypothesis testing, the test statistic (e.g. *F*, *t*, *r*) with confidence intervals, effect sizes, degrees of freedom and *P* value noted<br>*Give P values as exact values whenever suitable.*
☒	☐	For Bayesian analysis, information on the choice of priors and Markov chain Monte Carlo settings
☐	☒	For hierarchical and complex designs, identification of the appropriate level for tests and full reporting of outcomes
☐	☒	Estimates of effect sizes (e.g. Cohen's *d*, Pearson's *r*), indicating how they were calculated

Our web collection on statistics for biologists contains articles on many of the points above.

Software and code

Policy information about availability of computer code

Data collection	FLIR tools (software for running thermal camera and recording videos)
Data analysis	Origin Pro 8, packages “effects”, “pbkrtest”, and “lmer” in R (version 3.4.1)

For manuscripts utilizing custom algorithms or software that are central to the research but not yet described in published literature, software must be made available to editors/reviewers. We strongly encourage code deposition in a community repository (e.g. GitHub). See the Nature Research guidelines for submitting code & software for further information.

Data

Policy information about availability of data

All manuscripts must include a data availability statement. This statement should provide the following information, where applicable:

- Accession codes, unique identifiers, or web links for publicly available datasets
- A list of figures that have associated raw data
- A description of any restrictions on data availability

The source data underlying Figs. 1, 2, 4, 6, and 7 and Supplementary Figs. 4, 6-9, 32, 33, 35, 37-39, 42, and 43 are provided as a Source Data file.

Field-specific reporting

Please select the one below that is the best fit for your research. If you are not sure, read the appropriate sections before making your selection.

☐ Life sciences ☐ Behavioural & social sciences ☒ Ecological, evolutionary & environmental sciences

For a reference copy of the document with all sections, see nature.com/documents/nr-reporting-summary-flat.pdf

Ecological, evolutionary & environmental sciences study design

All studies must disclose on these points even when the disclosure is negative.

Study description	The work involves studing optical properties of dried butterfli wing specimens from he Museum of Comparative Zoology (MCZ), Harvard University, and behaviorial studies of live butterflies collected from the field. Genus, Species, Specimen ID, Collection location, G. I. S., and Date of dried wing specimens and live butterflies are provided in Supplementary Table 6. Number of live specimens for each species used in the study is indicated in Fig. 6 of the main text and Supplementary Table 7.
Research sample	Live butterfly specimens have been used in this study. These include 862 individuals representing 50 species in six of the seven known families of butterflies (Papilionidae, Hesperiidae, Pieridae, Nymphalidae, Lycaenidae, and Riodinidae). This choice is to provide a wide coverage of butterfly species in order to assess the similarities in their response to light stimulation. In addition, dried butterfly wing specimens have been analyzed in this study, and these are from the Pierce DNA and Tissues Collection of the Museum of Comparative Zoology (MCZ), Harvard University.
Sampling strategy	No specific selection regarding species or sex was made during field collection of butterflies. All live specimens that have wings largely intact were used in behaviorial studies.
Data collection	Cheng-Chia Tsai and Norman Nan Shi took optical and infrared spectra of butterfly wings. Cheng-Chia Tsai and Nanfang Yu recorded behavior of live butterflies using an infrared camera and a visible photo camera.
Timing and spatial scale	Butterfly collection in the field were conducted between April 8, 2017 and June 17, 2019, mostly during the weekends from local gardens and parks of New Jersey and New York near Columbia University. Experiments and data collection on live insects were conducted within one week of the collection of the live specimens.
Data exclusions	No data were excluded from analysis.
Reproducibility	When butterfly specimens are available, behavior studies were conducted on at least 6 female and 6 male butterflies of the same species.
Randomization	Individuals of the same species were allocated into the same group. If total individuals of a species is larger than 20, male and female specimens were analyzed separately.
Blinding	During behavior studies of live butterflies, the sex of some species were not identified during data collection. Sexual dimorphism in the response of butterflies to light stimulation was investigated later during the data analysis period.

Did the study involve field work? ☒ Yes ☐ No

Field work, collection and transport

Field conditions	Live butterflies used in this study were collected during the later spring and summer of 2017, 2018, and 2019.
Location	Gardens and parks of New Jersey and New York near Columbia University
Access and import/export	NA
Disturbance	No endangered butterfly species were collected from the field. All live butterflies studied are common species.

Reporting for specific materials, systems and methods

We require information from authors about some types of materials, experimental systems and methods used in many studies. Here, indicate whether each material, system or method listed is relevant to your study. If you are not sure if a list item applies to your research, read the appropriate section before selecting a response.

Materials & experimental systems

n/a	Involved in the study	
☒	☐	Antibodies
☒	☐	Eukaryotic cell lines
☒	☐	Palaeontology
☐	☒	Animals and other organisms
☒	☐	Human research participants
☒	☐	Clinical data

Methods

n/a	Involved in the study	
☒	☐	ChIP-seq
☒	☐	Flow cytometry
☒	☐	MRI-based neuroimaging

Animals and other organisms

Policy information about studies involving animals; ARRIVE guidelines recommended for reporting animal research

Laboratory animals	NA
Wild animals	Butterfly species and quantities of male and female specimens for each species are provided in Fig. 6 of the main text. The butterflies were collected from the field using inset nets. They were killed after the study by freezing in a fridge and have been deposited in the Pierce DNA and Tissues Collection of the Museum of Comparative Zoology (MCZ), Harvard University
Field-collected samples	Live butterflies were collected from the field for this study. These are common, non-endangered species from New Jersey and New York. Butterflies are most abundant during sunny and warm morning hours.
Ethics oversight	NA

Note that full information on the approval of the study protocol must also be provided in the manuscript.
