# Supplementary material for: Physical and behavioral adaptations to prevent overheating of the living wings of butterflies
Source: Nat Commun. 2020 Jan 28;11:551. doi: 10.1038/s41467-020-14408-8 (PMC6987309; doi:10.1038/s41467-020-14408-8)
Supplement: Supplementary file 3 — Description of Additional Supplementary Files [file 41467_2020_14408_MOESM3_ESM.pdf]

Description of Additional Supplementary Files

File Name: Supplementary Movie 1
Description: Distribution of methylene-stained sensory neurons in the hindwing veins of Vanessa cardui. Note that in these images, the membranes of the wing are white saturated.

File Name: Supplementary Movie 2
Description: Distribution of methylene-stained sensory neurons in the forewing veins of Satyrium caryaevorus.

File Name: Supplementary Movie 3
Description: Tidal flow of hemolymph in a radial vein of Vanessa cardui.

File Name: Supplementary Movie 4
Description: Hemolymph flow (with hemocytes highlighted) within the scent pad of Parrhasius malbum, and the beating of wing heart.

File Name: Supplementary Movie 5
Description: Hemolymph flow within the scent pad of Parrhasius m-album, and the beating of wing heart.

File Name: Supplementary Movie 6
Description: Hemolymph flow (with hemocytes highlighted) within the scent pad of Satyrium caryaevorus.

File Name: Supplementary Movie 7
Description: Hemolymph flow within the scent pad of Satyrium caryaevorus.

File Name: Supplementary Movie 8
Description: Thermal camera video of a number of species of butterflies during laser-induced local heating experiments.

File Name: Supplementary Movie 9
Description: Visible and thermal camera videos taken during laser-induced local heating experiments with Parrhasius m-album. The laser spot focused on the butterfly was produced by a telecom laser at λ=1.55 μm

File Name: Supplementary Movie 10
Description: Visible camera videos taken during basking experiments with Satyrium caryaevorus at low and high ambient temperatures.

File Name: Supplementary Movie 11
Description: Visible camera videos taken during basking experiments with Parrhasius m-album at low and high ambient temperatures.

File Name: Supplementary Movie 12
Description: Visible camera video taken in the field to show that adults of Satyrium caryaevorus almost always bask in the sun within a few seconds after landing.
